# The decrease in histone methyltransferase EZH2 in response to fluid shear stress alters endothelial gene expression and promotes quiescence

**DOI:** 10.1007/s10456-015-9485-2

**Published:** 2015-09-28

**Authors:** Monika Maleszewska, Byambasuren Vanchin, Martin C. Harmsen, Guido Krenning

**Affiliations:** Cardiovascular Regenerative Medicine Research Group, Department of Pathology and Medical Biology, University Medical Center Groningen, University of Groningen, Hanzeplein 1 (EA11), 9713 GZ Groningen, The Netherlands; Max Planck Research Group Chromatin and Ageing, Max Planck Institute for Biology of Ageing, Joseph-Stelzmann-Str. 9b, 50931 Cologne, Germany

**Keywords:** Endothelial cell, Enhancer of zeste homolog-2 (EZH2), Fluid shear stress (FSS), Mechanotransduction, Chromatin

## Abstract

High uniform fluid shear stress (FSS) is atheroprotective and preserves the endothelial phenotype and function through activation of downstream mediators such as MAPK7 (Erk5). Endothelial cells respond to FSS thanks to mechanotransduction. However, how the resulting signaling is integrated and resolved at the epigenetic level remains elusive. We hypothesized that Polycomb methyltransferase EZH2 is involved in the effects of FSS in human endothelial cells. We showed that FSS decreases the expression of the Polycomb methyltransferase EZH2. Despite simultaneous activation of MAPK7, MAPK7 pathway does not directly influence the transcription of EZH2. Interestingly though, the knockdown of EZH2 activates the protective MAPK7 signaling in endothelial cells, even in the absence of FSS. To understand the influence of the FSS-decreased expression of EZH2 on endothelial transcriptome, we performed RNA-seq and differential gene expression analysis. We identified candidate groups of genes dependent on both EZH2 and FSS. Among those, Gene Ontology overrepresentation analysis revealed highly significant enrichment of the cell cycle-related genes, suggesting changes in proliferation. Indeed, the depletion of EZH2 strongly inhibited endothelial proliferation, indicating cell cycle arrest. The concomitant decrease in CCNA expression suggests the transition of endothelial cells into a quiescent phenotype. Further bioinformatical analysis suggested TXNIP as a possible mediator between EZH2 and cell cycle-related gene network. Our data show that EZH2 is a FSS-responsive gene. Decreased EZH2 levels enhance the activation of the atheroprotective MAPK7 signaling. Decrease in EZH2 under FSS mediates the decrease in the expression of the network of cell cycle-related genes, which allows the cells to enter quiescence. EZH2 is therefore important for the protective effects of FSS in endothelium.

**Electronic supplementary material:**

The online version of this article (doi:10.1007/s10456-015-9485-2) contains supplementary material, which is available to authorized users.

## Introduction

Endothelial cells constitute the lining of all blood vessels and are therefore exposed to the fluid shear stress (FSS)—the frictional force exerted on the vessel wall by the flow of blood [1, 2]. Geometrical features of the arterial tree, such as the aortic curve and branches, cause alterations in the patterns of blood flow. At these so-called atheroprone sites, FSS is low or even absent, which correlates with the increased susceptibility of these sites to endothelial dysfunction and atherosclerosis [3–5].

Endothelial cells sense FSS through mechanotransduction. The FSS-induced activation of the MAP2K5–MAPK7 (MEK5–Erk5) signaling pathway, which is sustained under prolonged exposure to FSS [6], exerts protective effects on the endothelium [7–9]. MEK5 activates MAPK7 through phosphorylation [8, 10], which results in expression of Kruppel-like factor-2 and 4 (KLF2 and KLF4), transcription factors that drive the expression of atheroprotective genes [8, 11].

Gene expression is regulated at the chromatin level through the deposition or removal of epigenetic modifications by specialized enzymes. These modifications, to histone proteins or to the DNA itself, shape the accessibility of gene promoters to the transcriptional machinery. In particular, Polycomb repressive complexes are crucial regulators of gene expression, with well-established roles during development and carcinogenesis [12]. Enhancer of zeste homolog-2 (EZH2) is the main methyltransferase in the Polycomb repressive complex-2 (PRC2). EZH2 methylates histone-3 at lysine-27 (H3K27me3 mark), which maintains the repression of gene expression [13].

The epigenetic events that mediate cellular responses to mechanical forces, such as the endothelial response to FSS, are still poorly understood. EZH2 regulates the differentiation of mechanosensing Merkel cells in the skin [14]. EZH2 was also shown to regulate endothelial gene expression and function [15–17]. However, the link between EZH2 and endothelial mechanotransduction in response to FSS has not been reported.

We hypothesized that EZH2, through epigenetic regulation of gene expression, mediates the response of endothelial cells to the mechanical force of FSS.

## Materials and methods

### Cell culture and fluid shear stress experiments

Human umbilical vein endothelial cells (HUVEC; Lonza, Basel, Switzerland) were used between passages 5 and 8, cultured in endothelial cell medium (ECM) as described before [18], but with 5.5 mM glucose and 10 % heat-inactivated fetal calf serum (FCS; Lonza, Basel, Switzerland), in gelatin-coated dishes. For the FSS experiments, µ-Slides I 0.4 Luer (Ibidi, Planegg/Martinsried, Germany) were coated with gelatin, and HUVEC were seeded at full confluency (approximately 60,000 cells/cm^2^) and incubated overnight under standard static cell culture conditions. Slides with confluent cell monolayers were attached to a fluidic unit (Ibidi, Planegg/Martinsried, Germany), connected to the pump (Ibidi, Planegg/Martinsried, Germany), and incubated under standard cell culture conditions in 5 % FCS ECM. Inverted pressure was used to ensure the gas exchange in the culture medium. Fluid shear stress (FSS) of 20 dyne/cm^2^ was applied to the monolayers in the slides, for 72 h. Static controls were cultured in the same incubator and the same medium, refreshed daily. In the stop-flow experiments, after FSS was ceased, cells were incubated for an additional 1 h in static conditions before they were lysed. MAP2K5–MAPK7 (MEK5–Erk5) pathway inhibitor BIX02189 was used at the concentration of 5 µM. Simvastatin (Sigma-Aldrich, St. Louis, MO, USA) was used at the concentration of 1 µM, for 24 h. Appropriate volumes of DMSO were used in controls.

Human embryonic kidney (HEK) cells and Phoenix-Ampho cells were cultured in 10 % FCS DMEM (Lonza, Basel, Switzerland), 2 mM l-glutamine (Lonza, Basel, Switzerland), and 1 % penicillin/streptomycin (Gibco/Thermo Fisher Scientific, Wiltham, MA, USA).

### Viral transduction

In MEK5D expression experiments, Phoenix-Ampho cell line stably expressing and producing retroviral particles with empty vector (pBABE-puro-EV) or constitutively active MEK5 (MAP2K5; pBABE-puro-MEK5D) was used. Cells were cultured at subconfluent densities. The collection of the viral particles was done in 10 % FCS ECM medium, starting 24 h after the last preceding passage. The supernatants were collected two times at 24 h intervals, filtered through 0.45-µm filters and applied to 30 % confluent HUVEC cultures. Twenty-four hours after the last transduction medium was refreshed, and cells were cultured until confluent. Upon selection with 2 µg/ml of puromycin (Invitrogen, Carlsbad, CA, USA), cells were allowed to proliferate and then were lysed for further analysis.

For lentiviral transductions to obtain the EZH2 knockdown, HEK cells were transfected using Endofectin™-Lenti (Gene Copoeia, Rockville, MD, USA, EFL-1001-01) with the following plasmids: pLKO.1-shEZH2 or pLKO.1-SCR, pVSV-G (envelope plasmid) and pCMVΔR8.91 (*gag*–*pol* second generation packaging plasmid). Virus collection was started the day after, in 10 % FCS ECM medium. At 24-h intervals, 30 % confluent HUVEC were transduced twice. Every first transduction was done with 4 µg/ml polybrene. After the last transduction, cells were allowed to proliferate for another 3 days and were then selected with 2 µg/ml of puromycin. Surviving cells were allowed to proliferate for another 24 h. At this point, 7 days post-first transduction, cells were used for downstream experiments or analyses. The whole procedure was repeated for each replicate. A complete knockout of EZH2 (no protein present in western blotting analyzes) was confirmed in all EZH2 knockdown cells used in the experiments in this study.

### siRNA transfection

HUVEC were seeded subconfluent and transfected at 80–90 % confluency, in 12-well plates. Cells were washed with PBS and pre-incubated with 400 µl of OptiMEM (Invitrogen, Carlsbad, CA, USA) per well at 37 °C. Transfection mixes were prepared with lipofectamine (Invitrogen, Carlsbad, CA, USA) and siRNA against EZH2 (Hs_EZH2_4 FlexiTube siRNA, cat. no. SI00063973) or AllStars Negative Control siRNA (cat. no. 1027280, QIAGEN, Venlo, The Netherlands), and a 100 µl of an appropriate mix containing 30 pmol of siRNA was added per a well. Cells were incubated at 37 °C for 6 h, then washed two times with PBS, and cultured further in regular culture medium. Medium was refreshed once more 48 h post-transfection. Cells were lysed 72 h post-transfection.

### RNA isolation and real-time PCR

Cells were lysed with either RNA-Bee (TEL-TEST, Inc., Friendswood, TX, USA) or TriZOL (Invitrogen, Carlsbad, CA, USA). To isolate RNA, standard phenol/chloroform extraction was performed in accordance with the manufacturer’s guidelines, followed by isopropanol precipitation. RNA pellets were washed twice with ice-cold 75 % ethanol, dried, and resuspended in RNAse-free water. Concentrations were measured by spectrophotometry (NanoDrop/Thermo Fisher Scientific, Waltham, MA, USA). cDNA was synthesized with the RevertAid™ First Strand cDNA Synthesis Kit (Thermo Fisher Scientific, Wiltham, MA, USA). Real-time PCR (ViiA7 Real-Time PCR system, Applied Biosystems, Foster City, CA, USA) was performed with 150 nmol of primers and 10 ng of cDNA input per reaction, using SYBR Green chemistry (BioRad, Hercules, CA, USA, or Roche, Basel, Switzerland). Data were analyzed with the ViiA7 software (Applied Biosystems, Foster City, CA, USA) and further processed in Excel. Geometrical mean of *ACTB* and *GAPDH* Ct values, or only *GAPDH* Ct values (consistent within an experimental set), was used for the ΔC_t_ normalization as follows: $\Delta {\text{C}}_{\text{t}} = {\text{C}}_{{{\text{t}}_{{{\text{Gene}}\;{\text{of}}\;{\text{interest}}}} }} - {\text{C}}_{{{\text{t}}_{{{\text{Housekeeping}}\;{\text{genes}}}} }}$. Fold change over control samples was calculated using ΔΔC_t_ method, as $2^{{ - {\Delta \Delta }{\text{C}}_{\text{t}} }}$, where ${\Delta \Delta }{\text{C}}_{\text{t}} =\Delta {\text{C}}_{{{\text{t}}_{\text{control}} }} -\Delta {\text{C}}_{{{\text{t}}_{\text{treatment}} }}$.

Primers used in this study are shown in Table 1.

**Table 1:** Primer sequences used in the study

Gene symbol	Primer sequence 5′–3′
*ACTB*	Forward: CCAACCGCGAGAAGATGA
	Reverse: CCAGAGGCGTACAGGGATAG
*CCNA1*	Forward: GGGGCTCCCAGATTTCGTCT
	Reverse: CAGCACAACTCCACTCTTGG
*CCNA2*	Forward: GAGGCCGAAGACGAGACG
	Reverse: CTTTCCAAGGAGGAACGGTGA
*CCNB1*	Forward: CGGCCTCTACCTTTGCACTT
	Reverse: GGCCAAAGTATGTTGCTCGAC
*CCNB2*	Forward: TGCGTTGGCATTATGGATCG
	Reverse: AAGCCAAGAGCAGAGCAGTA
*CDC20*	Forward: ATTCGCATCTGGAATGTGTGC
	Reverse: TGTAATGGGGAGACCAGAGGA
*DSCC1*	Forward: CCGGACCAGTTGAAGAAGGAA
	Reverse: GGGTCTACGTCTTCTTAATTCCC
*KIF20A*	Forward: ACTGCTCTGTCGTCTCTACCT
	Reverse: GGTAACAAGGGCCTAACCCTC
*NCAPG*	Forward: CACCAGAACCAGGCGAAG
	Reverse: GAAAAACTGTCTTATCATCCATCG
*NOS3*	Forward: CACATGGCCTTGGACTGAA
	Reverse: CAGAGCCCTGGCCTTTTC
*MAPK7*	Forward: CCTGATGTCAACCTTGTGACC
	Reverse: CCTTTGGTGTGCCTGAGAAC
*EZH2*	Forward: GCGAAGGATACAGCCTGTGCACA
	Reverse: AATCCAAGTCACTGGTCACCGAAC
*GAPDH*	Forward: AGCCACATCGCTCAGACAC
	Reverse: GCCCAATACGACCAAATCC
*KLF2*	Forward: CATCTGAAGGCGCATCTG
	Reverse: CGTGTGCTTTCGGTAGTGG
*KLF4*	Forward: GGGAGAAGACACTGCGTCA
	Reverse: GGAAGCACTGGGGGAAGT

### Western blotting

Cells were lysed with RIPA buffer (Thermo Fisher Scientific, Wiltham, MA, USA), freshly supplemented with proteinase inhibitor cocktail and phosphatase inhibitor cocktails-2 and 3 (all from Sigma-Aldrich, St. Louis, MO, USA). Electrophoresis was performed in 10 % polyacrylamide gels, followed by electrotransfer onto nitrocellulose membranes. Membranes were blocked with Odyssey Blocking Buffer (Li-COR Biosciences, Lincoln, NE, USA) 1:1 in Tris-buffered Saline (TBS) for 1 h at room temperature (RT). Blots were then incubated with primary antibodies at 4 °C, overnight, and afterwards with secondary antibodies for 1 h at RT. The membranes were washed three times with TBS with 0.1 % Tween in between incubations and additionally with TBS before the scanning. Odyssey scanner (Li-COR Biosciences, Lincoln, NE, USA) was used to retrieve the digital images of the membranes. These were analyzed with Odyssey software (Li-COR Biosciences, Lincoln, NE, USA), and densitometry was performed with TotalLab 120 software (Nonlinear Dynamics, Newcastle, UK). Images depicted in figures were processed in Adobe Photoshop and Illustrator, and if necessary, brightness of a whole image was adjusted in linear fashion.

The following antibodies were used: NOS3/eNOS (1:1000, BD Biosciences, San Jose, CA, USA, 610299), MAPK7/Erk5 (1:500, Upstate/Merck Millipore, Billerica, MA, USA, 07-039), EZH2 (1:1000, Cell Signaling, Danvers, MA, USA, 5246), GAPDH (1:1000, Abcam, Cambridge, UK, ab9485 or ab9484), KLF2 (1:250, Santa Cruz Biotechnology, Dallas, TX, USA, sc-28675), KLF4 (1:250, Santa Cruz Biotechnology, Dallas, TX, USA, sc-20691), cyclin A (1:500, Santa Cruz Biotechnology, Dallas, TX, USA, sc-751), cyclin B1 (1:500, Santa Cruz Biotechnology, Dallas, TX, USA, sc-s45), cyclin E (1:500, Santa Cruz Biotechnology, Dallas, TX, USA, sc-247), anti-rabbit IgG IRDye-680LT (1:10 000, Li-COR Biosciences, Lincoln, NE, USA, 926-68021), and anti-mouse IgG IRDye-800CW (1:10 000, Li-COR Biosciences, Lincoln, NE, USA, 926-32210).

### RNA-seq

Puromycin-selected HUVEC cells, expressing either scrambled control (SCR) or anti-EZH2 short-hairpin (shEZH2) constructs (at total 7 days after the first viral transduction), were used in FSS experiments (72 h of control static culture or FSS exposure). Each replicate experiment consisted of viral transductions (described above) and selection of a separate HUVEC batch, followed by the FSS experiment. Two FSS experimental sets of the same HUVEC batch were run every time in parallel and lysed at the same end time point, one in RNAse-free conditions with RNA-Easy Mini Plus kit RLT Plus lysis buffer (QIAGEN, Venlo, The Netherlands) and one with RIPA buffer. The RIPA lysates were analyzed with western blotting and confirmed the complete (no protein present) knockdown of EZH2.

From the RNA lysates, RNA was isolated using the RNA-Easy Mini Plus kit (QIAGEN, Venlo, the Netherlands). High-quality RNA samples (pre-assessed by Nanodrop measurements) were further processed in the Genome Analysis Facility of the University Medical Center Groningen. The RNA quality and integrity were verified using PerkinElmer Labchip GX with a cutoff value of 9 (scale 1–10, where 9 is very high-quality RNA). RNA library was created in accordance with the TruSeq™ RNA Sample Preparation v2 Guide (Illumina, San Diego, CA, USA), using the PerkinElmer Sciclone liquid handler, resulting in 330-bp cDNA fragments. The paired-end sequencing (100-bp reads) was performed using the Illumina HiSeq™ 2500.

Sequencing data were analyzed using the Tuxedo pipeline [19], with TopHat2 (v. 0.6), Cufflinks (v. 0.0.6), Cuffmerge (v. 0.0.6), CuffDiff (v. 0.0.7), as available at the public Galaxy platform usegalaxy.org as of August 2014 [20–22]. Prior to the alignment, FASTQ Groomer (v. 1.0.4) was used to groom the .fq files, and FastQC (v. 0.52) was used to assess the quality of the reads. Trim sequences tool (v. 1.0.0) was used to trim the reads. Picard Insertion size metrics tool (v. 1.56.0) was used to estimate the distance between mate pairs (paired-end reads). Reads were aligned to the hg_19, and iGenomes hg_19 (v. 1.1.3) was used for annotation.

Differential expression analysis was performed with CuffDiff, with FPKM (Fragments Per Kilobase of exon per Million fragments mapped) normalization method and false discovery rate (FDR) correction, where corrected *p* values (*q* values) <0.05 were considered to indicate significant changes.

The CuffDiff output was explored using CummeRbund (v. 0.1.3) in R-Studio 0.98. For the comparisons of interest, the gene sets of significantly differentially expressed genes were extracted at *α* = 0.05.

For a scheme of the subsequent analysis flow, please refer to the Online Figure 5. Gene Ontology (GO) [23] enrichment analysis was performed using the PANTHER database, at the www.PANTHERdb.org Web site (PANTHER 9.0), as of August 2014 [24]. Gene lists were analyzed with the overrepresentation tool. The Bonferroni correction for multiple testing was applied, and the corrected *p* value (*q* value) of 0.05 was considered the cutoff for significantly overrepresented terms.

The intersection of the lists of genes was performed with the BioVenn tool [25]. The Venn diagrams were plotted using the R package VennDiagram.

Pathway enrichment analysis was performed using KEGG database using the Enrichr tools available at the Enrichr Web site (with the combined ranking method) [26].

REVIGO online tool was used to organize and visualize the enriched GO terms obtained from the PANTHER 9.0; *q* values obtained in PANTHER GO enrichment analysis were used as the rating parameter in REVIGO (only the terms with *q* < 0.05 were used) [27].

STRING 9.1 tool was used to explore the mutual relationships between the products of the genes [28, 29].

Additional information on the genes of interest, that can be found in the online Tables, was retrieved from Ensembl [30], using the BioMart tool [31].

The data discussed in this publication have been deposited in NCBI’s Gene Expression Omnibus [32] and are accessible through GEO Series accession number GSE71164 (http://www.ncbi.nlm.nih.gov/geo/query/acc.cgi?acc=GSE71164).

### Ki67 immunofluorescent staining

HUVEC expressing either scrambled control (SCR) or SH-EZH2 constructs were seeded at density of 25,000 cells per well in 24-well plates in 2 % FCS ECM and incubated for 24 h. After that cells were washed with PBS, fixed with 2 % paraformaldehyde for 30 min, washed with PBS, permeabilized with 0.5 % Triton X in PBS for 10 min, washed with PBS, and blocked with 10 % donkey serum in PBS. Cells were then incubated with primary antibodies, rabbit-anti-human Ki67 (1:500, Monosan, PSX1028) in 10 % donkey serum in PBS, while negative controls were incubated with the 10 % donkey serum in PBS, at 4 °C overnight. Cells were then washed with PBS with 0.05 % Tween-20 and incubated with secondary antibodies, donkey-anti-rabbit IgG Alexa Fluor-555 (1:500, Life Technologies, Carlsbad, CA, USA, A31572) in 10 % human serum in PBS with DAPI (1:5000), for 40 min at RT. Cells were next washed with PBS with 0.05 % Tween and with PBS, the plates were scanned, and images were taken in an automated manner with the Tissue FAXS microscope (TissueGnostics, Vienna, Austria). Exposure of the images was optimized and regulated by the software, and unprocessed images were used in the quantitative analysis, performed with the Tissue Quest 4.0.1.0127 software (TissueGnostics, Vienna, Austria), which counts the positive cells and measures the fluorescence intensity. The data were normalized by dividing the Ki67-positive cell numbers by all DAPI-positive cell numbers. The brightness of the representative images depicted in Fig. 7 was adjusted in a linear manner and to the same extent in each image, to better visualize the stained cells.

### Statistical analysis

Statistical analysis was performed in GraphPad Prism 4 or 5 (La Jolla, CA, USA), with *t* test, or one-way ANOVA followed by post hoc tests with corrections specified in figure legends. Graphs depict mean and standard deviation or SE of the mean (specified in figure legends), and the number of independent experiments is indicated in the dot plots and in figure legends. *p* values <0.05 were considered to indicate a significant difference between means.

## Results

### Fluid shear stress regulates EZH2 expression in endothelial cells

**Fig. 1:**
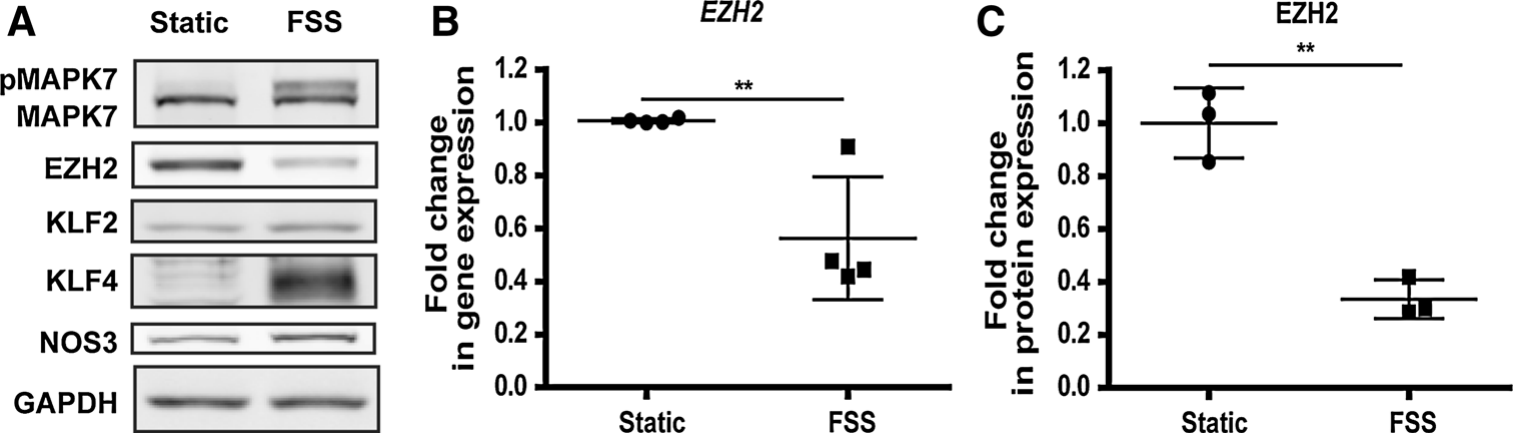
FSS causes a decrease in EZH2 gene and protein expression. HUVEC were cultured for 72 h under 20 dyne/cm^2^ FSS. **a** Representative western blotting images. **b** Gene expression of EZH2 under FSS, *n* = 4, **p < 0.01, *t* test and **c** protein expression of EZH2 under FSS, obtained through the densitometry of the western blotting data, *n* = 3, **p < 0.01, *t* test

FSS of 20 dyne/cm^2^ decreased the expression of EZH2 in HUVEC (Fig. 1a–c). As expected, it also activated MAPK7 signaling (Fig. 1a) and increased expression of KLF2, KLF4, and endothelial nitric oxide synthase (NOS3/eNOS) (Online Fig. 1 A–F).

**Fig. 2:**
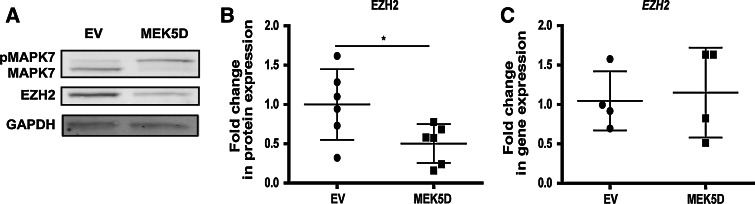
Protein expression of EZH2 is decreased along MAPK7 activation. The constitutively active MEK5 mutein (MEK5D) was expressed in HUVEC. **a** Representative western blotting images. **b** Protein expression of EZH2 in cells expressing MEK5D, obtained through the densitometry of the western blotting data, *n* = 6, *p < 0.05, *t* test and **c** gene expression of EZH2 in cells expressing MEK5D, *n* = 4

To evaluate whether the decrease in EZH2 expression under FSS is a result of MAPK7 activation, we expressed MEK5D, a constitutively active mutant of MEK5/MAP2K5 [8], in endothelial cells. MEK5D expression resulted in activation of MAPK7 (Fig. 2a) and increased the expression of *KLF2* and *KLF4* (Online Fig. 2A and B), confirming that the model worked properly. MAPK7 activation coincided with decreased expression of EZH2 at the protein level, but not at the mRNA level (Fig. 2a–c). Pharmacological inhibition of MAPK7 activation by the small molecule inhibitor BIX02189 did not rescue the expression of EZH2 decreased by FSS (Online Fig. 3A) or by treatment with simvastatin (Online Fig. 3B). These data suggested that while FSS decreases the expression of EZH2 in HUVEC, MAPK7 is not involved in mediating this effect.

### Depletion of EZH2 enhances MAPK7 activation

**Fig. 3:**
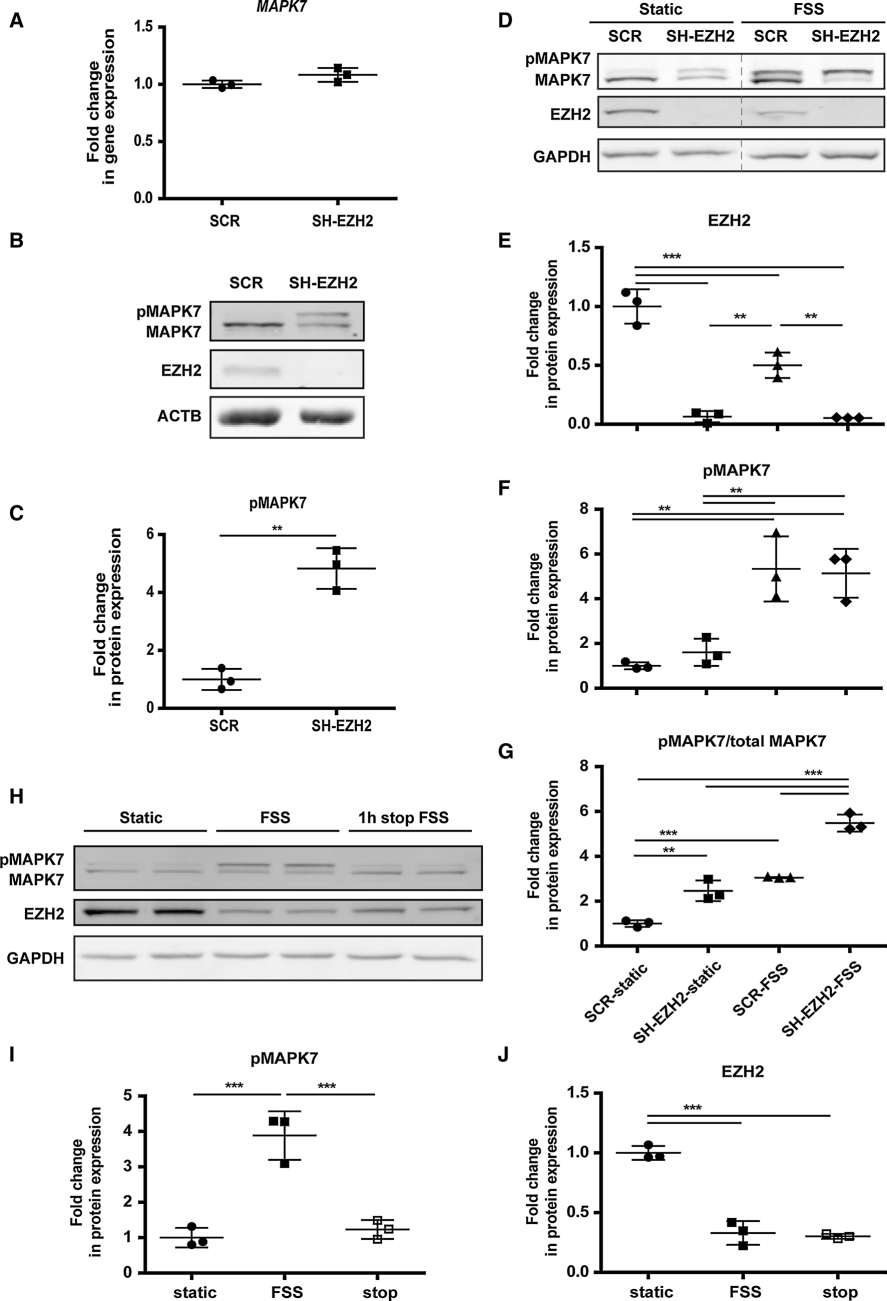
EZH2 levels determine the activation capacity of MAPK7. **a** Gene expression of MAPK7 in cells depleted of EZH2. Anti-EZH2 shRNA was expressed in HUVEC for 7 days by means of lentiviral delivery. Scrambled shRNA was used as control, *n* = 3. **b** Representative images of western blotting showing the enhanced activation of MAPK7 in static conditions upon 7-day knockdown of EZH2 in HUVEC. **c** Densitometry results showing the enhanced activation of MAPK7 in static conditions upon the knockdown of EZH2, derived from the western blotting data, normalized to β-actin (ACTB), *n* = 3, **p < 0.01, *t* test. **d** Representative western blotting images, showing enhanced activation of MAPK7 in EZH2-depleted cells compared to control, both in static and in FSS-exposed cultures. *Dashed line* indicates where the images were artificially connected: they are parts of the same membrane (one image) and were only moved to depict the lanes in the order which is easier for interpretation. Control and EZH2-depleted HUVEC were cultured under FSS for 3 days. **e** Densitometry results of the western blotting data showing the protein expression levels of EZH2, *n* = 3, **p < 0.01, ***p < 0.001, one-way ANOVA with Tukey’s post hoc comparisons between all pairs of means. **f**, **g** Densitometry results of the western blotting data showing the total phosphorylation levels of MAPK7 (normalized to GAPDH) and the ratio of phosphorylated MAPK7 to total expressed MAPK7 (both normalized to GAPDH), respectively, *n* = 3, **p < 0.01, ***p < 0.001, one-way ANOVA with Tukey’s post hoc comparisons between all pairs of means. **h** Representative western blotting images showing rapid dephosphorylation of MAPK7 upon the cessation of the flow. Cells were cultured for 72 h in static conditions or under FSS; afterwards one group was kept for an additional 1 h in static conditions before cells lysis (“1-h stop FSS”) and **i**, **j** Densitometry results showing the levels of MAPK7 phosphorylation and EZH2 protein expression, respectively, normalized to GAPDH. The “stop” caption refers to the 1-h stop FSS condition (see **h**), *n* = 3, ***p < 0.001, one-way ANOVA with Tukey’s post hoc comparisons between all pairs of means

Although it did not directly regulate the expression of EZH2, MAPK7 is an important mediator of FSS in endothelial cells. We therefore investigated how, on the other hand, the FSS-induced decrease in EZH2 affects the expression and activity of MAPK7. Knockdown of EZH2 by either shRNA or siRNA did not alter the gene expression levels of *MAPK7* in endothelial cells (Fig. 3a and Online Fig. 4A and B). However, knockdown of EZH2 did increase the basal phosphorylation levels of MAPK7 under static conditions (Fig. 3b, c) as well as enhanced the activation of MAPK7 in the cells exposed to FSS (Fig. 3d–g). These data imply that the levels of EZH2 determine the activation capacity of MAPK7 in endothelial cells. As MAPK7 activation is maintained upon prolonged exposure to FSS [6], we checked whether the decrease in EZH2 expression under FSS would modulate the deactivation (dephosphorylation) of MAPK7 after FSS was stopped. The decrease in EZH2 under FSS (Fig. 3h, i) did not affect the dephosphorylation of MAPK7 within 1 h after the FSS exposure was stopped, as the MAPK7 phosphorylation levels were diminished to a level comparable with static control samples. These data suggest that MAPK7 activation, rather than deactivation, is affected by the decrease in EZH2 (Fig. 3h, j, “1-h stop FSS”/“stop”).

### EZH2 regulates genes involved in cell adhesion and cell cycle in endothelial cells

To understand the role of EZH2 in the regulation of transcription in endothelial cells in response to FSS, we employed a transcriptomic approach (Online Fig. 5). First, the genes regulated by the knockdown of EZH2 or by FSS were explored separately, to gain insight into the groups of genes affected by either condition. Then, the groups of genes regulated by both EZH2 depletion and FSS exposure were identified. In both cases, Gene Ontology overrepresentation analysis was performed to classify the differentially expressed genes and to identify the most significantly enriched groups of genes (which are likely to be highly biologically relevant in the conditions tested). Overview of the analysis is presented in Online Fig. 5.

**Fig. 4:**
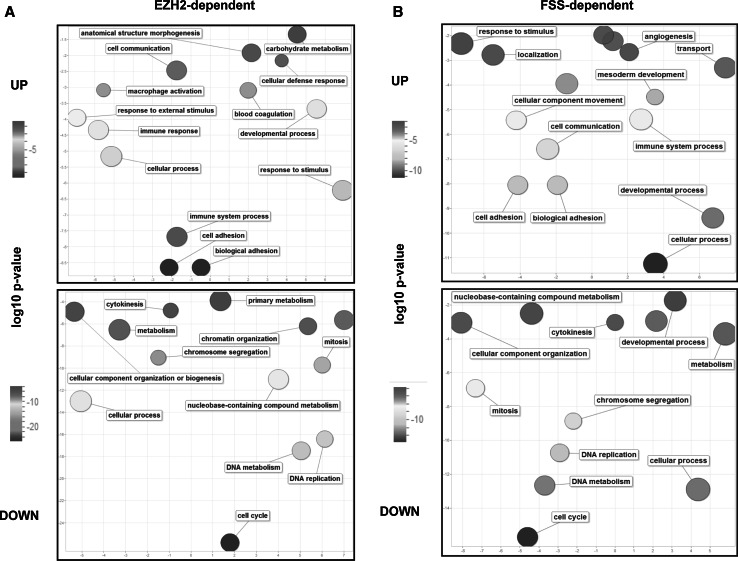
Biological process Gene Ontology terms which were significantly overrepresented among genes regulated by EZH2 (*panel a*) or by FSS (*panel b*). The figure shows the REVIGO representation of GO BP terms enriched in the lists of genes regulated twofold or more upon EZH2 depletion (**a**) or FSS exposure (**b**). The lists of GO significantly enriched terms (cutoff *q* < 0.05) were obtained through the overrepresentation analysis using PANTHER 9.0. The size of a bubble corresponds to the size of the group of genes in the analysis, belonging to the specific GO term. *UP* upregulated genes, *DOWN* downregulated genes. For the exact (corrected) *p* values, please compare Supplementary Tables 2, 3, 5 and 6

RNA-seq analysis of control (SCR) and EZH2-depleted (SH-EZH2) cells showed that the depletion of EZH2 in endothelial cells increased the expression of 2042 genes (*q* < 0.05), of which 550 were increased ≥twofold (Online Table 1). Overrepresentation analysis of these genes using PANTHER database revealed that the most significantly overrepresented biological process (BP) Gene Ontology (GO) term was cell adhesion (Fig. 4a upper panel and Online Table 2), with 58 genes (Online Fig. 6A). Of 2654 genes whose expression was decreased in cells depleted of EZH2 (*q* < 0.05), 760 genes were ≥twofold decreased (Online Table 1). The most overrepresented group within these 760 genes was the genes associated with the BP GO term cell cycle (Fig. 4a lower panel, and Online Table 3), including 136 genes (Online Fig. 6B).

### FSS-regulated genes in endothelial cells

Next, we analyzed the transcriptomic effects of FSS in endothelial cells. Exposure of endothelial cells to FSS increased the expression of 2142 genes (*q* < 0.05) of which 615 genes were increased ≥twofold (Online Table 4), with the most significantly overrepresented groups within the BP GO terms cellular process, developmental process, and cell adhesion (Fig. 4b upper panel, Online Fig. 7A and Online Table 5). FSS decreased the expression of 3035 genes (*q* < 0.05), of which 835 genes were ≥twofold decreased (Online Table 4). The most enriched group was associated with the BP GO term cell cycle (Fig. 4b lower panel, Online Fig. 7B and Online Table 6).

### Identification of candidate genes regulated by EZH2 in response to FSS in endothelial cells

**Fig. 5:**
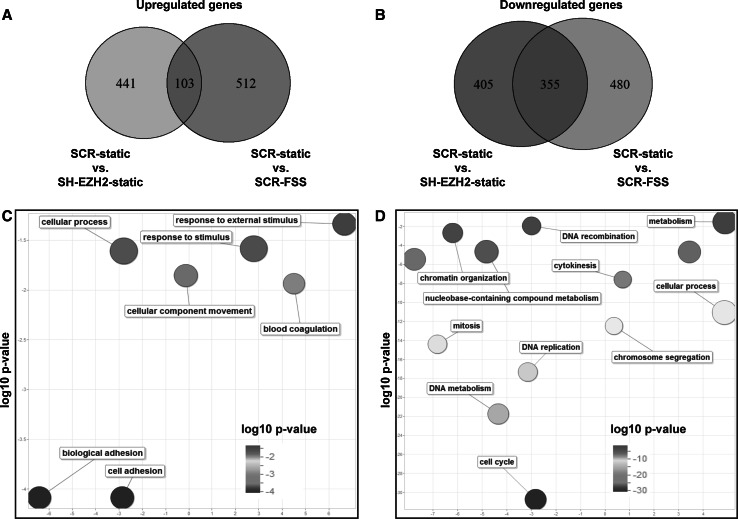
Genes regulated by both EZH2 and FSS are the most significantly enriched within GO terms cell adhesion and cell cycle. **a** Area-proportional Venn diagram, depicting the intersection of the lists of genes upregulated by EZH2 depletion (SCR-static vs. SH-EZH2-static) and genes upregulated by exposure to FSS (SCR-static vs. SCR-FSS). **b** Area-proportional Venn diagram, depicting the intersection of the lists of genes downregulated by EZH2 depletion (SCR-static vs. SH-EZH2-static) and genes downregulated by exposure to FSS (SCR-static vs. SCR-FSS). **c** REVIGO-derived representation of the most significantly overrepresented GO BP terms in the list of 103 genes upregulated by both EZH2 depletion and FSS exposure and **d** REVIGO-derived representation of the most significantly overrepresented GO BP terms in the list of 355 genes downregulated by both EZH2 depletion and FSS exposure. The significantly enriched GO terms (*q* < 0.05) were derived from the PANTHER 9.0 overrepresentation analysis. The GO enrichment *q* values are available in Supplementary Tables 9 and 10

We next set out to identify the genes that are affected by both EZH2 and FSS, which are the candidate genes regulated by the decrease in EZH2 under FSS. The expression of 103 genes increased, and the expression of 355 genes decreased upon both the depletion of EZH2 and the exposure to FSS (Fig. 5a, b, and Online Tables 7 and 8).

The group of 103 genes with increased expression was the most significantly enriched in genes belonging to the BP GO term cell adhesion (Fig. 5c and Online Table 9). The group of 355 genes with decreased expression was the most significantly enriched in genes associated with the BP GO term cell cycle (Fig. 5d, right panel and Online Table 10).

Additional pathway enrichment analysis with Enrichr using KEGG database showed significant enrichment of terms cell adhesion molecules (genes with increased expression, Online Fig. 8A) and cell cycle (genes with decreased expression, Online Fig. 8B).

### The FSS-exerted decrease in EZH2 inhibits endothelial proliferation through downregulation of cell cycle-associated network of genes

The expression of genes associated with the term cell adhesion, increased by the depletion of EZH2 and exposure to FSS, was in most cases also increased in the EZH2-depleted cells under FSS (Online Fig. 9A). However, most of these genes have not been reported to interact with each other (Online Fig. 9B), as they did not seem to form a functional network based on String 9.1 analysis. We therefore continued with the analysis of the Cell cycle-associated genes.

**Fig. 6:**
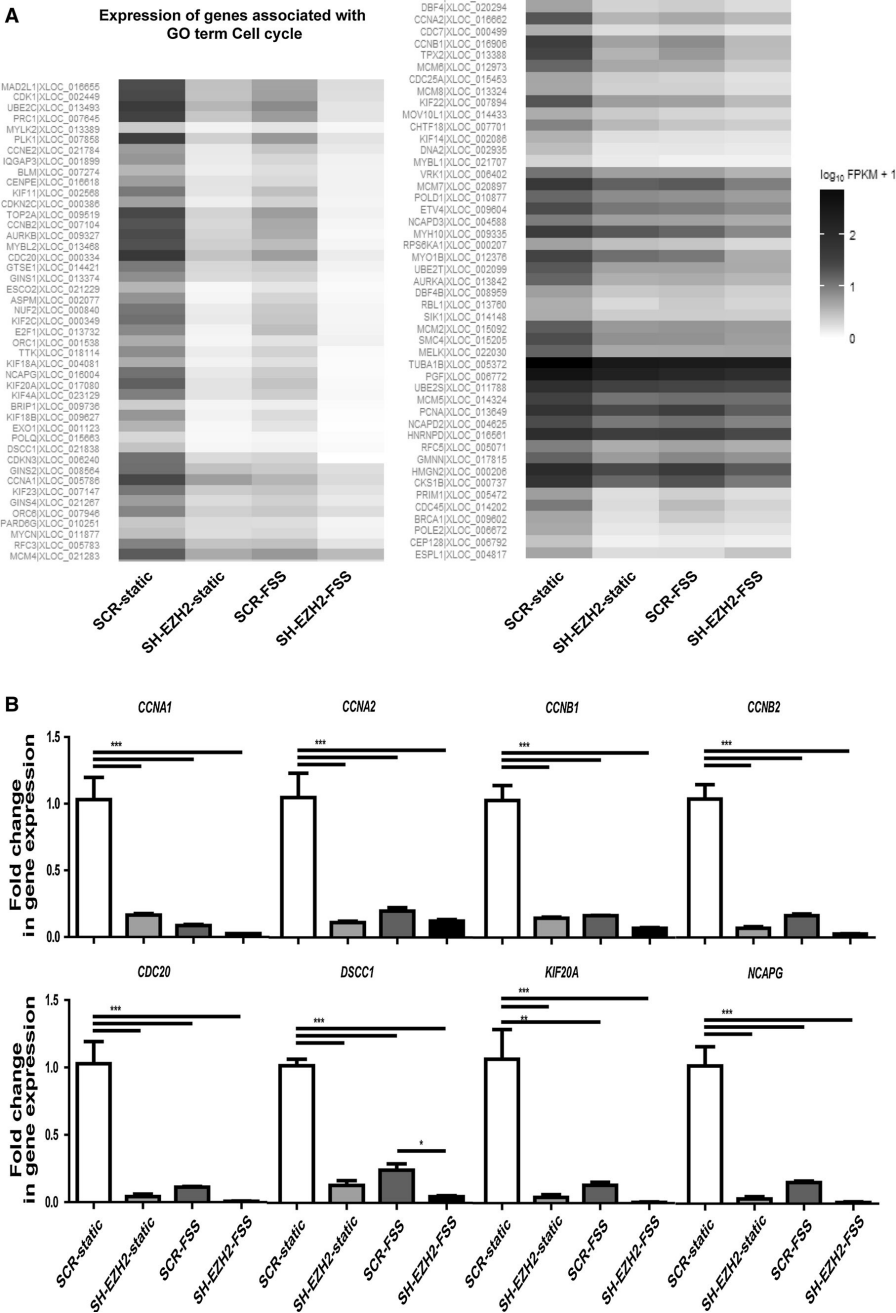
Cell cycle-associated genes are a candidate group of genes regulated by the decrease in EZH2 upon FSS. **a** Heatmap representation of relative expression levels of genes associated with the BP GO term cell cycle, which were regulated by both EZH2 depletion and FSS exposure and **b** Real-time PCR validation of the RNA-seq results for a subgroup of the cell cycle-associated genes, *n* = 3, *error bars* depict SE of the mean, **p < 0.01, ***p < 0.001 one-way ANOVA with Tukey’s post hoc comparisons between all pairs of means

The expression of genes associated with the GO term cell cycle was decreased/suppressed by EZH2 depletion, by FSS exposure, and in EZH2-depleted cells under FSS (Fig. 6a). Real-time PCR validation of a subset of these genes confirmed this expression pattern (Fig. 6b). It further confirmed that the expression of master regulators of cell cycle progression such as *CCNA2*, *CCNB1*, or *CCNB2* [33] was decreased by both FSS and the depletion of EZH2 in endothelial cells (Fig. 6b).

**Fig. 7:**
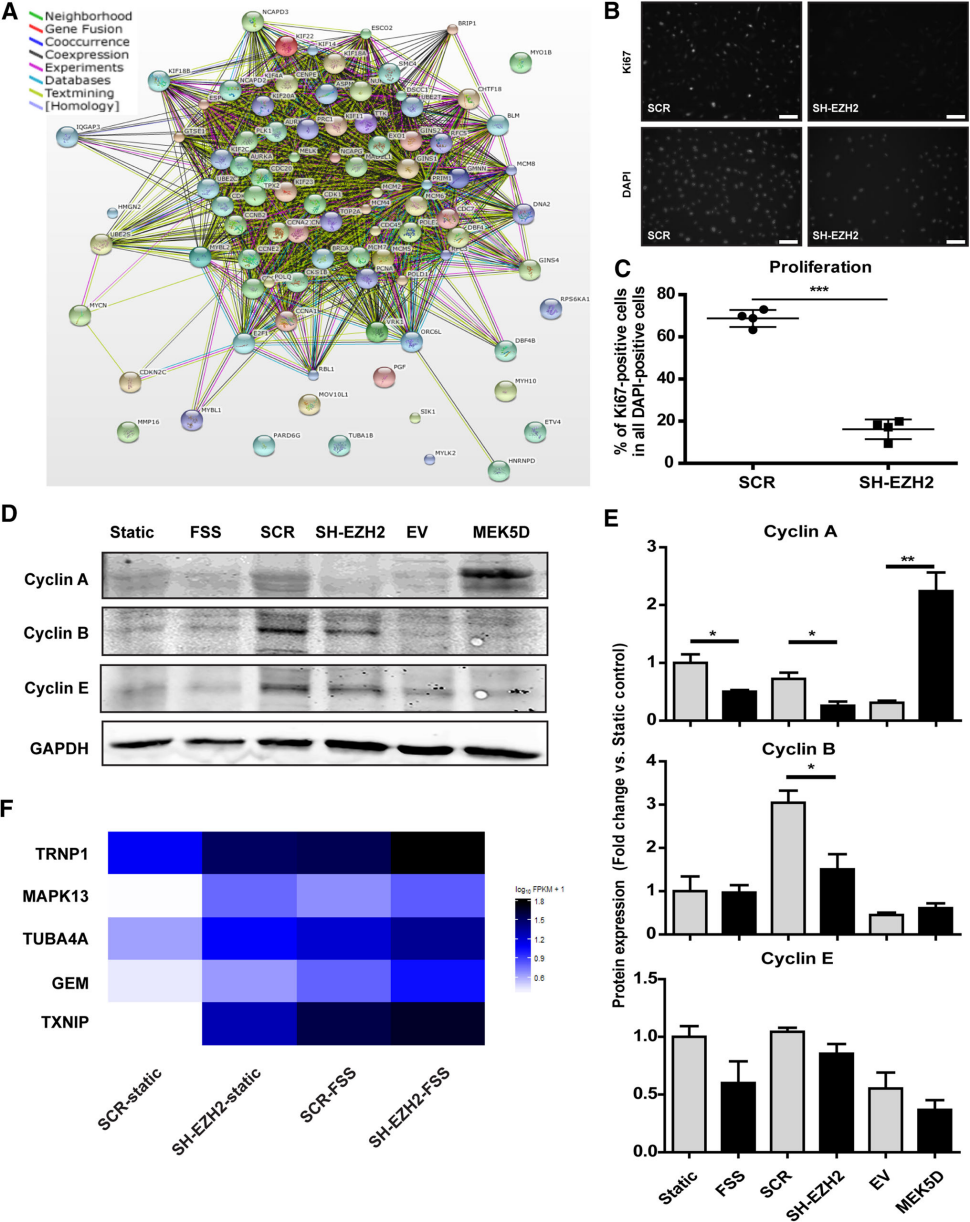
Downregulation of the network of cell cycle-associated genes leads to the decrease in proliferation of endothelial cells. **a** Products of the genes associated with the GO term cell cycle, which are regulated by both EZH2 and FSS, form a network of interdependencies. The list of genes regulated by both EZH2 and FSS belonging to the GO term cell cycle (most significantly enriched group) was analyzed using String 9.1. Depicted is the evidence view of interactions between the gene products. **b** Representative images of the immunofluorescent staining detecting the Ki67 protein expression in scrambled control (SCR) and EZH-depleted (SH-EZH2) endothelial cells (*upper panel*). *Lower panel* depicts DAPI signal, indicating nuclear staining. The *white bars* indicate 100 µm. **c** The average percentage of proliferating cells, derived as the percentage of Ki67-positive cells among all the DAPI-positive cells [i.e., Ki67-positive cell numbers are normalized to the number of all cells (DAPI) in the quantified region], showing the decrease in proliferation capacity of the EZH2-depleted cells. These results were obtained through the analysis with the TissueFAXS TissueQuest software, *n* = 4, ***p < 0.001, *t* test. **d** Representative western blotting results of protein expression of cyclins A, B and E. **e** Densitometric quantification of the protein expression of cyclins A, B and E, normalized to GAPDH. *n* = 4, *p < 0.05, **p < 0.01, Student *t* test, *error bars* depict SE of the mean and **f** Heatmap representation of the relative expression of MAPK13, TRNP1, TUBA4A, GEM, and TXNIP, the cell cycle-related genes whose expression was increased by both EZH2 depletion and FSS exposure in HUVEC

Most of the products of the cell cycle-related genes we identified (Fig. 6a) were interconnected by mutual relationships (Fig. 7a). These results suggested that the decrease in expression of these cell cycle-related genes could be a part of an orchestrated response, regulated by FSS through the decrease in EZH2, and aimed at the inhibition of cell cycle progression and proliferation.

To confirm that low levels of EZH2 functionally inhibit the cell cycle, we demonstrated that the depletion of EZH2 indeed decreased the proliferation rates of endothelial cells (Fig. 7b, c). These data suggested that the decrease in EZH2 (under FSS) could lead to the cell cycle arrest.

To further validate this notion, we analyzed the protein expression of chosen cyclins whose expression was decreased in our transcriptomic data. While cyclin B (CCNB) and cyclin E (CCNE) did not show consistent changes in protein expression, cyclin A (CCNA) protein levels were decreased both by the depletion of EZH2 and by the exposure to FSS (Fig. 7d, e). These patterns of expression, in particular the decrease in cyclin A levels, seemed to be specifically dependent on EZH2 and independent from the MEK5/MAPK7 pathway, as they were not observed in MEK5D-expressing cells (Fig. 7d, e).

As EZH2 is an epigenetic repressor, there could be other gene products, likely repressors, which are upregulated and mediate between the decrease in EZH2 availability and the decrease in cell cycle-related gene expression. To explore this possibility, we performed an additional GO overrepresentation analysis of all the genes regulated by EZH2 depletion and FSS exposure (up- and downregulated genes together). As could be expected, the GO term cell cycle was once more the most significantly overrepresented. In addition to the downregulated genes identified before (Fig. 6a), this analysis revealed five cell cycle-related genes, *MAPK13*, *TRNP1*, *TUBA4A*, *GEM*, and *TXNIP*, whose expression was increased in EZH2-depleted and in FSS-exposed cells (Fig. 7f). This small group of genes provides a set of potential mediators between EZH2 and the downregulated cell cycle-regulating genes.

## Discussion

We demonstrated that EZH2 is a fluid shear stress (FSS)-responsive gene. EZH2 levels influence the activation levels of MAPK7. EZH2 regulates the expression of multiple groups of genes in endothelial cells. In particular, it regulates the genes associated with cell adhesion and cell cycle. The FSS-induced decrease in EZH2 levels elicits an orchestrated response of cell cycle-regulating genes, which leads to inhibition of endothelial cell proliferation and likely to quiescence.

Our data altogether suggest that high FSS might keep the EZH2 expression levels low, which preserves the protected, quiescent state of endothelium. On the other hand, in case of low or absent FSS, e.g., in atheroprone arterial regions, high expression of EZH2 could contribute to endothelial dysfunction, e.g., by releasing endothelial cells from quiescence and promoting their (excessive) proliferation.

We demonstrated that high FSS is able to decrease the expression of the global epigenetic regulator, EZH2, at both mRNA and protein level. This decrease in expression of EZH2 seems to mediate some of the beneficial effects of FSS. Our RNA-seq analysis identified groups of genes dependent both on the EZH2 levels and on the presence of FSS. It is not surprising that there are several groups of genes (as classified by Gene Ontology terms) that are affected: on one hand, FSS is an important factor regulating many aspects of endothelial cell biology, and on the other hand EZH2 is a global epigenetic regulator, acting on multiple genomic *loci*. In the current study, we focused on the most significantly enriched group of cell cycle-related genes. However, exploration of the other groups of genes identified in this study could provide further examples of singular pathways regulated by FSS through decrease in EZH2.

The mechanism of the regulation of EZH2 expression by FSS remains to be fully elucidated. However, we succeeded in demonstrating that the major known FSS-induced pathway, MEK5/MAPK7 pathway, is of minor importance for regulation of EZH2 expression. Other pathways should be assessed in future studies. Furthermore, we established an exciting novel feedback link between EZH2 and MAPK7 pathway, by showing that MAPK7 activation capacity is increased when EZH2 levels decrease. This means that the protective, long-term activation of MAPK7 by high FSS could be mediated by the FSS-induced decrease in EZH2 levels.

A few other studies that so far reported on the role of EZH2 in endothelial cells confirm that EZH2 is involved in the regulation of endothelial gene expression and endothelial function [15, 16]. In particular, EZH2 regulates angiogenesis in the tumor microenvironment, where it is itself regulated by VEGF-miRNA-101 axis [16, 17, 34]. One study has so far addressed the role of EZH2 in endothelial cells with a global approach, similar to ours, but in static conditions only. Dreger et al. [15] studied the short-term effects of a transient (siRNA-mediated) knockdown of EZH2 in HUVEC. They reported enrichment of cell communication and cell adhesion-related genes among the genes regulated by EZH2, which corroborates our finding that the cell adhesion genes are the most enriched group among the genes upregulated by the knockdown of EZH2. The main difference between the studies is that we used a stable and long-term knockdown of EZH2 (total knockdown time of 10 days). Our approach allowed us to study more downstream (and secondary) effects of EZH2 depletion, which correspond well to the effects of the continuously low EZH2 levels under prolonged exposure to FSS. These long-standing effects are more similar, and likely more relevant, to the conditions of continuous blood flow and FSS in the blood vessels.

Therefore, our results extend the current knowledge on the role of EZH2 in endothelial cells, investigated so far only in static conditions, by providing insights into the role of EZH2 under mechanical force of FSS.

FSS also affects some of the other epigenetic regulators, such as histone deacetylases (HDACs) [35–37] and miRNAs [37, 38]. Moreover, recent studies demonstrated the role of DNA methylation in mediating the effects of FSS in endothelium, further substantiating the importance of epigenetic mechanisms in mediating the mechanosignaling [39, 40]. Our study is the first to add Polycomb and the histone methyltransferase EZH2 to the group of epigenetic-level regulators of endothelial response to FSS.

The genes related to GO term cell cycle were the most significantly enriched group regulated by the decrease in EZH2 and by FSS in our study. These genes form a dense network of interactions, suggesting that their products function together to regulate cell cycle progression. Indeed, for example, CDK1 is a major cell cycle regulator, which at different stages binds CCNA1 [41], CCNA2 [42], CCNB1 [43], and CCNB2 [44], and all of these genes were downregulated by EZH2 depletion and by FSS in our experiments. CDK1 also links directly to EZH2, as it can bind and phosphorylate EZH2 to change its epigenetic activity [45, 46]. The presence of these and many other concomitant interactions between the members of this group suggests that it is indeed a functional network, whose orchestrated downregulation serves to inhibit the cell cycle progression in endothelial cells. Indeed, our results show that depletion of EZH2 caused decrease in proliferation of endothelial cell, while others observed that high expression of EZH2 promotes the proliferation of many types of cancer cells [47–50]. These data imply that the decrease in EZH2 under FSS likely serves as the mechanism to downregulate the network of cell cycle regulators, therefore inhibiting the proliferation of endothelial cells.

EZH2 is an epigenetic repressor, which suggests that the decrease in EZH2 expression is more likely to induce expression of genes, rather than to decrease it. However, other groups investigating the transcriptomic effects of EZH2 also found that its inhibition or knockdown results in both increase and decrease in expression of genes, which is consistent with our findings [51, 52]. Nevertheless, we attempted to identify a possible link between the cell cycle-related genes and EZH2 in our study, by looking for an upregulated EZH2-dependent and cell cycle-related gene. The additional GO overrepresentation analysis revealed five cell cycle-related genes, MAPK13, TRNP1, TUBA4A, GEM, and TXNIP, which were upregulated by both EZH2 depletion and FSS exposure.

Of those candidate genes, TXNIP is the only one so far reported to be affected by EZH2. In the study by Zhou et al. [51], TXNIP expression was increased by EZH2 inhibition, which resulted in suppression of cell growth. These data are therefore consistent with our findings of TXNIP expression being upregulated and endothelial proliferation being inhibited in EZH2-depleted cells.

TXNIP (thioredoxin interacting protein, also known as VDUP1) is primarily related to oxidative stress regulation. However, it has been recognized as a tumor suppressor gene whose upregulation inhibits the growth of cancer cells [51, 53, 54]. This inhibitory effect of TXNIP has been linked to the cell cycle arrest in G1/G0 phase [53, 55]. Therefore, TXNIP is a likely mediator of the cell cycle arrest occurring after the decrease in EZH2 under FSS.

This could happen through the known TXNIP-dependent stabilization of p27 (CDKN1B) protein, which is a negative regulator of cell cycle [53, 54]. CDKN1B expression was indeed shown to be reversely correlated with EZH2 levels [56, 57]. Our results reproduced such increase in CDKN1B levels upon EZH2 depletion (Online Table 1). However, the CDKN1B expression was not affected by FSS in our dataset, suggesting that CDKN1B is not involved in FSS-induced inhibition of cell cycle.

Another potential target gene downstream of TXNIP is CCNA (cyclin A). The study by Han et al. [55] showed that TXNIP can act as a transcriptional repressor and is able to repress the promoter activity of CCNA2. CCNA expression was decreased in our experiments both at gene and protein level. Therefore, the EZH2-TXNIP-CCNA2 axis provides an interesting example of a link between EZH2 and cell cycle regulation. Nevertheless, it might be one of multiple connections feeding into the reported network of genes, while the whole network is important for the net effect of cell cycle inhibition.

The decrease in expression of EZH2 under FSS, along with the decrease in expression of cell cycle-regulating machinery, results in the decrease in proliferation, suggesting that the endothelial cells enter quiescence—the arrest of the cell cycle in G1/G0 phase. Endothelial cells are known to acquire a quiescent phenotype under high FSS [1, 11, 58]. Quiescence was also observed upon inhibition of EZH2 in multiple cell lines [59, 60]. In B lymphocytes, the decrease in EZH2 levels was necessary for entering the quiescent state [61]. Interestingly, both the increase in TXNIP and the decrease in cyclin A levels, consistently with our findings, have also been associated with G1/G0 arrest, and hence the quiescent phenotype [53, 55, 60, 62].

The quiescent state of endothelium under high FSS is deemed beneficial and protective for endothelium. Endothelial cells in the regions of disturbed flow proliferate intensively, which might result in their early senescence and contribute to the susceptibility of such vascular *foci* to atherosclerotic remodeling [1, 58]. We showed that the decrease in EZH2 levels also enhances the activation of MAPK7, a major FSS-responsive MAP kinase, which promotes atheroprotection through increased expression of KLF2, KLF4, and NOS3 [7–9, 63, 64]. Altogether, our results indicate that the suppression of EZH2 expression by high FSS is one of the mechanisms mediating the beneficial effects of high FSS in endothelial cells.

**Fig. 8:**
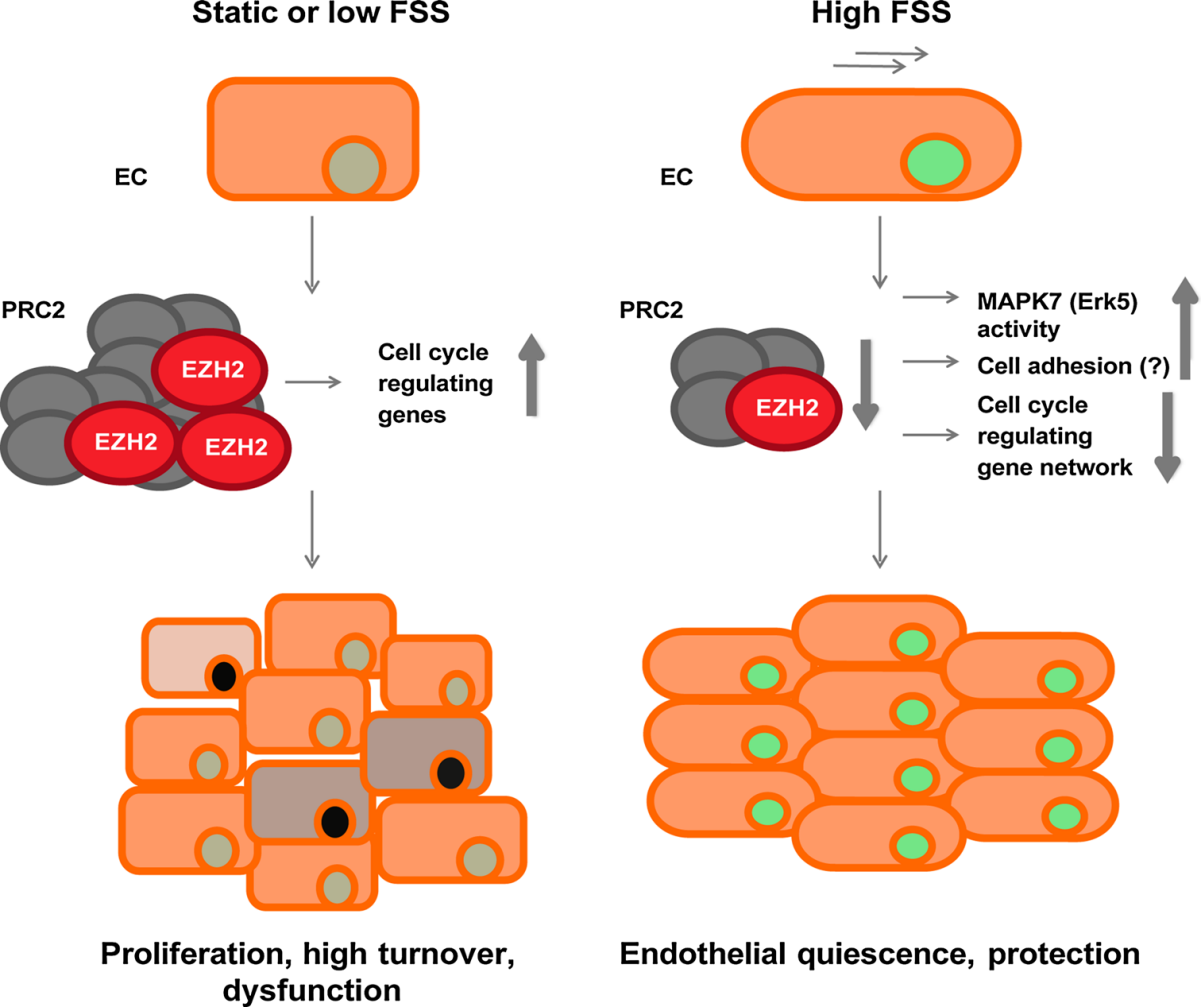
Graphical abstract showing the proposed mechanism of action of EZH2 under FSS

Our data establish EZH2 as a regulator of endothelial gene expression, involved in the endothelial response to FSS. In particular, we propose that the suppression of EZH2 expression by high FSS restricts the expression of a whole network of cell cycle-regulating genes, which results in the protected quiescent endothelial phenotype (Fig. 8). Given the atheroprotective role of high FSS and the availability of several EZH2 inhibitors, our results further suggest that EZH2 might become a promising pharmacological target to treat or prevent vascular disease.

## Electronic supplementary material

Supplementary material 1 (PDF 649 kb)

Supplementary material 2 (DOCX 4374 kb)
