# Supplementary material for: The decrease in histone methyltransferase EZH2 in response to fluid shear stress alters endothelial gene expression and promotes quiescence
Source: Angiogenesis. 2015 Sep 28;19:9–24. doi: 10.1007/s10456-015-9485-2 (PMC4700080; doi:10.1007/s10456-015-9485-2)
Supplement: Supplementary file 1 — Supplementary material 1 (PDF 649 kb) [file 10456_2015_9485_MOESM1_ESM.pdf]

Supplementary Table 1. Genes up- or down-regulated 2 times or more by the depletion of EZH2 in HUVEC.

Gene name	Log2 fold change	Fold change	p-value	q-value
LCP1	4.87	29.27	5e-05	0.000277
CXCL1	4.79	27.58	5e-05	0.000277
MYL9	4.58	23.84	5e-05	0.000277
HDAC9	4.40	21.04	5e-05	0.000277
MYPN	4.32	19.97	5e-05	0.000277
TAGLN	4.07	16.84	5e-05	0.000277
TXNIP	3.88	14.72	5e-05	0.000277
MFI2	3.76	13.56	5e-05	0.000277
KCNMB1	3.69	12.91	1e-04	0.000521
LUM	3.64	12.49	5e-05	0.000277
SERPINB2	3.62	12.30	5e-05	0.000277
CSF3	3.39	10.45	5e-05	0.000277
ATP6V0A4	3.37	10.34	0.001	0.003916
MAL2	3.33	10.05	0.006	0.017694
CLDN1	3.25	9.48	0.008	0.022585
SELP	3.23	9.37	5e-05	0.000277
ARRDC4	3.22	9.30	9e-04	0.003578
CXCL2	3.19	9.14	0.001	0.003402
TMEM38A	3.08	8.45	0.001	0.004437
TSPAN7	3.04	8.21	5e-05	0.000277
COL25A1	3.02	8.09	5e-05	0.000277
POU2F2	3.01	8.07	5e-05	0.000277
HLA-B	3.00	8.00	5e-05	0.000277
IL1A	2.97	7.85	5e-05	0.000277
PPP1R3C	2.87	7.33	5e-05	0.000277
GLT8D2	2.87	7.31	5e-05	0.000277
ST8SIA6	2.87	7.30	5e-05	0.000277
IL1R1	2.83	7.13	5e-05	0.000277
ART4	2.78	6.89	5e-05	0.000277
ANKRD29	2.78	6.88	0.002	0.008261
KLRG1	2.78	6.85	5e-05	0.000277
IL8	2.71	6.56	5e-05	0.000277
GVINP1	2.68	6.43	5e-05	0.000277
KCNK3	2.68	6.42	5e-05	0.000277
CXCL11	2.63	6.17	5e-05	0.000277
ULBP1	2.62	6.16	5e-05	0.000277
DOCK3	2.62	6.15	5e-05	0.000277
DYNC1I1	2.57	5.93	5e-05	0.000277
APBA1	2.56	5.89	5e-05	0.000277
CADM1	2.56	5.88	5e-05	0.000277
NT5E	2.55	5.86	5e-05	0.000277
AOX1	2.55	5.86	5e-05	0.000277
CERKL	2.55	5.85	0.000	0.001183
LINC00520	2.52	5.73	5e-05	0.000277
TSPAN8	2.49	5.60	4e-04	0.001790
C2orf66	2.47	5.56	0.004	0.012894
CCND2	2.46	5.52	5e-05	0.000277
DCBLD2	2.46	5.51	5e-05	0.000277
QPCT	2.45	5.48	5e-05	0.000277
HLA-B	2.45	5.45	5e-05	0.000277
LYZ	2.44	5.44	0.015	0.038347
VGLL3	2.38	5.20	5e-05	0.000277
FAS	2.37	5.15	5e-05	0.000277
TNFSF15	2.36	5.13	5e-05	0.000277
HLA-B	2.34	5.08	9e-04	0.003578
NCAM2	2.34	5.08	5e-05	0.000277
JAM2	2.31	4.97	1e-04	0.000521
SH3GL2	2.28	4.86	3e-04	0.001388
PLA2G4C	2.26	4.81	5e-05	0.000277
RECK	2.25	4.76	5e-05	0.000277
RGS11	2.25	4.75	5e-05	0.000277
FLRT3	2.25	4.74	5e-05	0.000277
DCN	2.24	4.71	0.000	0.001587
SERPINI1	2.23	4.70	0.000	0.001183
IL33	2.23	4.69	5e-05	0.000277
KIAA1024	2.23	4.69	5e-05	0.000277
BST1	2.22	4.65	5e-05	0.000277
CDK6	2.21	4.62	5e-05	0.000277
CPA3	2.19	4.56	5e-05	0.000277
XIRP2	2.18	4.52	5e-05	0.000277
C10orf128	2.17	4.51	4e-04	0.001790
MGAT4B	2.16	4.46	5e-05	0.000277
SLC16A6	2.16	4.46	0.002	0.005705
EDA	2.15	4.43	1e-04	0.000521
ANO4	2.14	4.40	5e-05	0.000277
AOC2	2.12	4.33	1e-04	0.000521
ICAM1	2.11	4.31	5e-05	0.000277
P2RX7	2.11	4.31	1e-04	0.000521
C5orf62	2.10	4.28	5e-05	0.000277
ACTA2	2.10	4.28	5e-05	0.000277
CYTL1	2.10	4.28	5e-05	0.000277
HPSE	2.09	4.25	5e-05	0.000277
COX7A1	2.08	4.24	5e-04	0.002168
OSTM1	2.08	4.24	5e-05	0.000277
COPZ2	2.08	4.23	0.000	0.000750
LOC201651	2.08	4.23	5e-05	0.000277
IL6	2.08	4.22	5e-05	0.000277
MYLK4	2.06	4.16	5e-05	0.000277
ENKUR	2.04	4.10	0.003	0.010988
TMEM163	2.04	4.10	5e-05	0.000277
GPX3	2.03	4.09	5e-05	0.000277

Supplementary Table 1 - continued

Gene name	Log2 fold change	Fold change	p-value	q-value
ENTPD3	2.03	4.09	5e-05	0.000277
VAT1L	2.03	4.08	5e-05	0.000277
DOK5	2.03	4.07	5e-05	0.000277
C14orf45	2.01	4.03	2e-04	0.000971
WDR66	2.01	4.02	1e-04	0.000521
NRK	2.00	4.00	5e-05	0.000277
SYNGR3	1.98	3.95	5e-05	0.000277
MAPK13	1.98	3.95	5e-05	0.000277
ITGA4	1.98	3.93	5e-05	0.000277
LOC100126784	1.96	3.88	0.002	0.006005
RAB6B	1.96	3.88	5e-05	0.000277
CPNE7	1.95	3.87	0.001	0.002718
ACP5	1.95	3.86	5e-05	0.000277
KBTBD8	1.95	3.86	5e-05	0.000277
NAALAD2	1.95	3.86	1e-04	0.000521
IRF6	1.95	3.86	5e-05	0.000277
SLIT3	1.94	3.84	5e-05	0.000277
PAPPA	1.92	3.80	5e-05	0.000277
CDKN2A	1.92	3.77	5e-05	0.000277
GGT7	1.91	3.76	5e-05	0.000277
TNFRSF10D	1.91	3.76	5e-05	0.000277
FOXP2	1.91	3.75	5e-05	0.000277
MAOA	1.91	3.75	4e-04	0.001790
GBP1	1.90	3.74	5e-05	0.000277
LOC729178	1.90	3.74	0.004	0.012182
LOC646999	1.88	3.69	5e-05	0.000277
PRNP	1.88	3.69	5e-05	0.000277
EPHA5	1.88	3.67	5e-05	0.000277
GLIPR1	1.87	3.66	5e-05	0.000277
LAMC2	1.87	3.65	5e-05	0.000277
NHLRC3	1.87	3.64	5e-05	0.000277
CXorf69	1.86	3.64	0.012	0.031547
ACOT1	1.86	3.63	0.014	0.035847
CD274	1.86	3.63	5e-05	0.000277
CYB5R2	1.85	3.60	0.006	0.017938
GLT25D1	1.85	3.60	5e-05	0.000277
ATF3	1.84	3.59	5e-05	0.000277
GALNT3	1.84	3.57	6e-04	0.002551
SPOCD1	1.83	3.57	5e-05	0.000277
PTGS1	1.82	3.54	5e-05	0.000277
MAGI2	1.82	3.53	0.010	0.026431
ITGA8	1.82	3.53	0.001	0.004601
MAP1A	1.82	3.52	5e-05	0.000277
TUBA4A	1.81	3.52	5e-05	0.000277
HMOX1	1.81	3.51	5e-05	0.000277
NEK11	1.81	3.50	5e-05	0.000277
MARVELD2	1.81	3.49	0.000	0.000750
ADPRH	1.80	3.48	1e-04	0.000521
ATP13A3	1.80	3.48	5e-05	0.000277
CPE	1.80	3.48	5e-05	0.000277
SLC44A5	1.79	3.47	5e-05	0.000277
ADAMTS13	1.79	3.46	0.008	0.022497
SGPP1	1.77	3.41	5e-05	0.000277
ERV3-1,ZNF117	1.77	3.41	5e-05	0.000277
SLC41A2	1.77	3.40	5e-05	0.000277
MAP2	1.76	3.39	5e-05	0.000277
MATN2	1.76	3.38	3e-04	0.001388
HS6ST3	1.76	3.38	5e-05	0.000277
CSMD2	1.76	3.38	5e-05	0.000277
PRKAB2	1.75	3.37	5e-05	0.000277
GARNL3	1.75	3.37	0.001	0.003747
ACVR2B	1.74	3.35	5e-05	0.000277
ITGB3	1.73	3.33	5e-05	0.000277
FBN1	1.73	3.33	5e-05	0.000277
RTN1	1.73	3.33	0.006	0.017938
SYT9	1.72	3.29	0.000	0.001981
ADAM23	1.72	3.28	5e-05	0.000277
ZC3H12A	1.71	3.28	5e-05	0.000277
TTC29	1.70	3.26	5e-05	0.000277
RENBP	1.70	3.25	5e-05	0.000277
FUCA1	1.69	3.23	5e-05	0.000277
GNS	1.69	3.22	5e-05	0.000277
LOC283070	1.68	3.21	0.002	0.006005
BVES	1.68	3.20	5e-05	0.000277
PXMP4	1.67	3.19	5e-05	0.000277
DACT1	1.66	3.16	0.000	0.001587
HTATIP2	1.66	3.15	5e-05	0.000277
TGFBR3	1.64	3.12	5e-05	0.000277
BAALC	1.64	3.12	6e-04	0.002551
ARSB	1.64	3.12	5e-05	0.000277
LOC100506730	1.64	3.12	2e-04	0.000971
PRRT3	1.64	3.11	5e-05	0.000277
TMEM217	1.64	3.11	5e-05	0.000277
PRUNE2	1.64	3.11	5e-05	0.000277
LOC399744	1.64	3.11	1e-04	0.000521
REEP2	1.63	3.10	0.000	0.001183
LIPA	1.63	3.09	5e-05	0.000277
SLC33A1	1.61	3.06	5e-05	0.000277
ADAMTS3	1.61	3.06	5e-05	0.000277
ACSF2	1.61	3.05	5e-05	0.000277
CA12	1.61	3.05	0.001	0.002358

Supplementary Table 1 - continued

Gene name	Log2 fold change	Fold change	p-value	q-value
TFEC	1.60	3.04	5e-05	0.000277
LAMA3	1.60	3.04	5e-05	0.000277
SLC45A1	1.60	3.03	2e-04	0.000971
ENPP4	1.60	3.03	5e-05	0.000277
DISP2	1.60	3.03	5e-05	0.000277
SYNE1	1.60	3.02	5e-05	0.000277
RCAN2	1.59	3.01	5e-05	0.000277
KIAA1467	1.58	3.00	5e-05	0.000277
IL11RA	1.58	2.99	3e-04	0.001388
CLIP4	1.58	2.99	5e-05	0.000277
ITGA5	1.58	2.99	5e-05	0.000277
DKK3	1.58	2.98	5e-05	0.000277
SLC39A6	1.58	2.98	5e-05	0.000277
C3orf52	1.57	2.98	5e-05	0.000277
LOC100507254	1.57	2.97	5e-05	0.000277
CTSA	1.56	2.95	5e-05	0.000277
ZNF610	1.56	2.94	5e-05	0.000277
FNIP2	1.55	2.94	5e-05	0.000277
CDCP1	1.55	2.94	5e-05	0.000277
CCND1	1.55	2.93	5e-05	0.000277
CD47	1.54	2.91	5e-05	0.000277
OVGP1	1.54	2.91	0.003	0.010024
NAALADL2	1.54	2.91	5e-05	0.000277
MME	1.54	2.91	5e-05	0.000277
GAS2	1.54	2.90	0.003	0.009450
ASAH1	1.53	2.89	5e-05	0.000277
FLJ33630	1.53	2.89	5e-05	0.000277
PIGZ	1.53	2.89	5e-05	0.000277
GNPTG	1.53	2.89	5e-05	0.000277
MCOLN1	1.53	2.88	5e-05	0.000277
CEND1	1.53	2.88	5e-05	0.000277
CETP	1.53	2.88	5e-05	0.000277
ADAM9	1.52	2.88	5e-05	0.000277
ERO1L	1.52	2.87	5e-05	0.000277
PPFIBP2	1.52	2.87	0.001	0.005385
TIMP2	1.52	2.87	5e-05	0.000277
FAM55C,NFKBIZ	1.52	2.87	5e-05	0.000277
MANBA	1.52	2.86	5e-05	0.000277
TMEM79	1.52	2.86	5e-05	0.000277
CIDECP	1.51	2.86	5e-05	0.000277
TMED5	1.51	2.85	5e-05	0.000277
SPATA18	1.50	2.84	5e-05	0.000277
PAMR1	1.50	2.83	0.004	0.013036
F3	1.50	2.83	2e-04	0.000971
DTX3	1.50	2.82	5e-05	0.000277
NCSTN	1.50	2.82	5e-05	0.000277
ITGAV	1.50	2.82	5e-05	0.000277
CTHRC1	1.49	2.81	5e-05	0.000277
IFI27	1.49	2.80	5e-05	0.000277
*PCDHA1,PCDHA10,PCDHA11 (...)	1.49	2.80	5e-05	0.000277
HEXB	1.49	2.80	5e-05	0.000277
CNTNAP1	1.49	2.80	5e-05	0.000277
PION	1.48	2.80	0.001	0.005233
CD40	1.48	2.79	5e-05	0.000277
TTLL7	1.48	2.79	5e-05	0.000277
TRNP1	1.47	2.78	5e-05	0.000277
HS6ST2	1.47	2.78	5e-05	0.000277
PLOD1	1.47	2.77	5e-05	0.000277
ANKRD1	1.46	2.76	5e-05	0.000277
HLA-B	1.46	2.76	5e-05	0.000277
GPR68	1.46	2.76	0.004	0.013277
SEMA4B	1.46	2.76	5e-05	0.000277
DNER	1.46	2.75	5e-05	0.000277
CBLN3	1.46	2.75	0.000	0.001587
LIMA1	1.46	2.75	5e-05	0.000277
SLC2A13	1.46	2.75	5e-05	0.000277
MGMT	1.45	2.74	5e-05	0.000277
CERS4	1.45	2.73	0.002	0.006906
PIK3IP1	1.45	2.73	5e-05	0.000277
OPTN	1.44	2.72	5e-05	0.000277
CCL2	1.44	2.72	5e-05	0.000277
FAM131B	1.44	2.72	5e-05	0.000277
GALNT12	1.44	2.71	5e-05	0.000277
TGFBR2	1.44	2.71	5e-05	0.000277
KSR2	1.44	2.71	5e-05	0.000277
STAC	1.44	2.71	1e-04	0.000521
IGFBP7	1.43	2.70	5e-05	0.000277
OBFC2A	1.43	2.70	5e-05	0.000277
PTX3	1.43	2.70	5e-05	0.000277
C14orf28	1.42	2.68	0.000	0.000750
IFI27L2	1.42	2.68	5e-05	0.000277
KAL1	1.42	2.67	5e-05	0.000277
LRRC37A3	1.42	2.67	5e-05	0.000277
MAN2B2	1.42	2.67	5e-05	0.000277
COL8A1	1.42	2.67	0.001	0.003747
HLA-B	1.42	2.67	0.007	0.019849
FICD	1.41	2.66	5e-05	0.000277
FBXL13	1.41	2.66	0.005	0.014573
IL7R	1.41	2.66	5e-05	0.000277
MIR31HG	1.41	2.66	5e-05	0.000277
TMEM150C	1.40	2.65	5e-05	0.000277

Supplementary Table 1 - continued

Gene name	Log2 fold change	Fold change	p-value	q-value
PLBD2	1.40	2.64	5e-05	0.000277
PARD3B	1.40	2.64	5e-05	0.000277
IL1RAPL1	1.39	2.63	0.001	0.005233
ITGA3	1.39	2.62	5e-05	0.000277
CCDC169,CCDC169-SOHLH2,SOHLH2	1.39	2.62	0.001	0.004601
MMP10	1.38	2.61	5e-05	0.000277
HMCN1	1.38	2.61	0.003	0.010310
COPS7A	1.38	2.61	5e-05	0.000277
PLSCR4	1.38	2.60	5e-05	0.000277
C6orf174,KIAA0408	1.38	2.60	5e-05	0.000277
PLD6	1.38	2.59	0.007	0.020354
C8orf4	1.37	2.59	5e-05	0.000277
POPDC3	1.37	2.59	5e-05	0.000277
DNAH1	1.37	2.58	5e-05	0.000277
SIAE	1.37	2.58	5e-05	0.000277
SLC20A2	1.36	2.57	5e-05	0.000277
IL17D	1.36	2.56	5e-05	0.000277
B4GALT5	1.36	2.56	5e-05	0.000277
LOC728730	1.35	2.56	0.003	0.011111
RABL2A	1.35	2.55	0.004	0.013417
SLC17A5	1.35	2.55	5e-05	0.000277
MBNL3	1.35	2.54	5e-05	0.000277
CECR1	1.34	2.54	0.001	0.003747
LACC1	1.34	2.54	5e-05	0.000277
ABCA9	1.34	2.54	5e-05	0.000277
SCNN1D	1.34	2.53	0.005	0.015890
C7orf10	1.34	2.53	0.002	0.007234
TNFRSF11A	1.34	2.53	1e-04	0.000521
ZFYVE26	1.34	2.53	5e-05	0.000277
LPCAT1	1.34	2.52	5e-05	0.000277
ADAM12	1.33	2.52	5e-05	0.000277
CBX7	1.33	2.51	5e-05	0.000277
NR1I3	1.32	2.50	0.009	0.025105
TSPAN11	1.32	2.50	5e-05	0.000277
PARP15	1.32	2.49	0.017	0.043118
FMNL1	1.32	2.49	1e-04	0.000521
ITFG1	1.32	2.49	5e-05	0.000277
CLIC2	1.32	2.49	5e-05	0.000277
LOC646329	1.31	2.49	0.008	0.023870
ARNTL	1.31	2.48	5e-05	0.000277
LMLN	1.31	2.48	5e-05	0.000277
C1orf101	1.31	2.48	0.006	0.018863
PTPN21	1.30	2.46	5e-05	0.000277
IFIH1	1.29	2.45	1e-04	0.000521
FAM47E,FAM47E-STBD1,STBD1	1.29	2.45	2e-04	0.000971
PANX2	1.29	2.45	5e-05	0.000277
LOC650623	1.29	2.44	5e-05	0.000277
BEND6	1.29	2.44	0.006	0.016921
IRAK2	1.29	2.44	5e-05	0.000277
CPEB2	1.28	2.44	5e-05	0.000277
REEP6	1.28	2.43	2e-04	0.000971
DPH3	1.28	2.43	5e-05	0.000277
TNFSF18	1.28	2.43	5e-05	0.000277
SCPEP1	1.28	2.43	5e-05	0.000277
SDC3	1.27	2.41	5e-05	0.000277
RRM2B	1.27	2.40	5e-05	0.000277
MRAP2	1.26	2.40	0.000	0.001183
CSF1	1.26	2.40	5e-05	0.000277
CTNS	1.26	2.40	0.006	0.019097
NAPB	1.26	2.40	5e-05	0.000277
GBA	1.26	2.39	5e-05	0.000277
UGGT2	1.26	2.39	5e-05	0.000277
RRAS	1.26	2.39	5e-05	0.000277
CPXM2	1.25	2.38	1e-04	0.000521
TTC3P1	1.25	2.38	0.008	0.022585
GRN	1.25	2.37	5e-05	0.000277
C11orf63	1.24	2.37	0.000	0.001183
TMEM116	1.24	2.37	0.006	0.017694
SMPD1	1.24	2.36	5e-05	0.000277
TEK	1.24	2.36	5e-05	0.000277
TMOD2	1.24	2.36	5e-05	0.000277
GDPD1	1.24	2.36	0.000	0.000750
MMP1	1.23	2.35	5e-05	0.000277
FAM3C	1.23	2.35	5e-05	0.000277
CYP2S1	1.23	2.35	5e-05	0.000277
NCEH1	1.23	2.34	5e-05	0.000277
FAM117A	1.23	2.34	2e-04	0.000971
ATP6AP1	1.22	2.34	5e-05	0.000277
SORBS1	1.22	2.33	5e-05	0.000277
RIT1	1.22	2.33	5e-05	0.000277
CTSS	1.22	2.33	5e-05	0.000277
MXD1	1.22	2.33	5e-05	0.000277
STS	1.22	2.33	5e-05	0.000277
RAG1	1.22	2.32	5e-05	0.000277
ACCS	1.22	2.32	0.001	0.003402
MORN4	1.21	2.32	5e-05	0.000277
ARHGAP6	1.21	2.32	0.003	0.011111
AMPD3	1.21	2.32	8e-04	0.003240
CEACAM19	1.21	2.32	5e-05	0.000277
DGAT2	1.21	2.31	0.000	0.001981
ABLIM3	1.21	2.31	5e-05	0.000277

Supplementary Table 1 - continued

Gene name	Log2 fold change	Fold change	p-value	q-value
SLC40A1	1.21	2.31	5e-05	0.000277
NFE2L3	1.20	2.31	5e-05	0.000277
DMPK	1.20	2.30	5e-05	0.000277
ZEB2,ZEB2-AS1	1.20	2.30	5e-05	0.000277
ZNF497	1.20	2.30	0.011	0.029627
DUSP3	1.20	2.30	5e-05	0.000277
LARGE	1.20	2.30	5e-05	0.000277
ADRB2	1.19	2.28	0.001	0.002358
DPY19L1	1.19	2.28	5e-05	0.000277
NAAA	1.19	2.28	5e-05	0.000277
LIFR	1.19	2.28	5e-05	0.000277
CFH	1.18	2.27	5e-05	0.000277
SLC9A9	1.18	2.27	5e-05	0.000277
RAI2	1.18	2.27	5e-05	0.000277
KIAA1324L	1.18	2.27	5e-05	0.000277
FHIT	1.18	2.27	0.005	0.015412
QRICH2	1.18	2.26	5e-05	0.000277
C6orf72	1.18	2.26	5e-05	0.000277
LAMC1	1.18	2.26	5e-05	0.000277
FAT1	1.17	2.26	5e-05	0.000277
VPS41	1.17	2.25	5e-05	0.000277
PTPLAD2	1.17	2.25	0.001	0.004601
TOR2A	1.17	2.25	5e-05	0.000277
CPQ	1.17	2.25	5e-05	0.000277
SEPW1	1.17	2.24	5e-05	0.000277
C1orf85	1.17	2.24	5e-05	0.000277
RSPH3	1.16	2.24	0.001	0.002358
DNAJC6	1.16	2.24	5e-05	0.000277
MSRB3	1.16	2.24	5e-05	0.000277
IL13RA1	1.16	2.24	5e-05	0.000277
SAMSN1	1.16	2.24	5e-05	0.000277
BAI2	1.16	2.23	0.000	0.001587
TNFAIP3	1.16	2.23	5e-05	0.000277
PKI55	1.15	2.23	5e-05	0.000277
TMEM59L	1.15	2.22	0.001	0.005385
HFE	1.15	2.22	0.001	0.003747
NIPAL1	1.15	2.22	0.005	0.014050
SLC9A6	1.15	2.22	5e-05	0.000277
CACNG7	1.15	2.22	5e-05	0.000277
C21orf7	1.15	2.22	5e-05	0.000277
HLA-C	1.15	2.22	5e-05	0.000277
TUSC3	1.15	2.21	5e-05	0.000277
CHAC1	1.15	2.21	7e-04	0.002898
ACPL2	1.15	2.21	0.003	0.010600
OSMR	1.14	2.21	5e-05	0.000277
B3GNT9	1.14	2.21	0.016	0.040274
PAM	1.14	2.21	5e-05	0.000277
HLA-C	1.14	2.20	5e-05	0.000277
FGF2	1.14	2.20	5e-05	0.000277
HENMT1	1.14	2.20	0.005	0.016393
ANTXR1	1.14	2.20	5e-05	0.000277
CLSTN1	1.14	2.20	5e-05	0.000277
PIK3R2	1.14	2.20	5e-05	0.000277
OSGIN1	1.14	2.20	5e-05	0.000277
CFP	1.14	2.20	0.019	0.046901
OCLN	1.13	2.20	5e-05	0.000277
PCYOX1L	1.13	2.19	5e-05	0.000277
FAM13B	1.13	2.19	5e-05	0.000277
SORBS2	1.13	2.19	5e-05	0.000277
PTK2B	1.13	2.19	0.002	0.006906
ALDH6A1	1.13	2.19	5e-05	0.000277
MIR4639,MYLIP	1.12	2.18	3e-04	0.001388
IL1RL1	1.12	2.18	5e-05	0.000277
C16orf52	1.12	2.18	5e-05	0.000277
TMEM9B	1.12	2.18	5e-05	0.000277
TMEM2	1.12	2.18	5e-05	0.000277
B7H6	1.12	2.18	5e-05	0.000277
IGIP	1.12	2.18	5e-05	0.000277
PRTFDC1	1.12	2.17	5e-05	0.000277
STK40	1.12	2.17	5e-05	0.000277
SAMD4A	1.12	2.17	5e-05	0.000277
GSN	1.12	2.17	5e-05	0.000277
CMTM4	1.12	2.17	5e-05	0.000277
PLA2G12A	1.12	2.17	5e-05	0.000277
CEP19	1.12	2.17	0.004	0.012742
PKD2	1.11	2.16	5e-05	0.000277
PIGG	1.11	2.16	5e-05	0.000277
LOC374443	1.11	2.16	0.004	0.011785
SPTBN5	1.11	2.15	5e-05	0.000277
SLC25A20	1.11	2.15	5e-05	0.000277
ATL1	1.10	2.15	5e-05	0.000277
ANGPT1	1.10	2.14	5e-05	0.000277
SGCB	1.10	2.14	5e-05	0.000277
HLA-C	1.10	2.14	5e-05	0.000277
FAP	1.10	2.14	5e-05	0.000277
LOC550112	1.10	2.14	5e-05	0.000277
ARPC3	1.10	2.14	5e-05	0.000277
NAP1L5	1.10	2.14	0.006	0.016787
RASA4	1.10	2.14	5e-05	0.000277
TPM1	1.10	2.14	5e-05	0.000277
ZNF154	1.09	2.14	4e-04	0.001790

Supplementary Table 1 - continued

Gene name	Log2 fold change	Fold change	p-value	q-value
SLC4A8	1.09	2.13	5e-05	0.000277
PTGFRN	1.09	2.13	5e-05	0.000277
PGRMC2	1.09	2.13	5e-05	0.000277
DNAJC10	1.09	2.13	5e-05	0.000277
C17orf108	1.09	2.13	0.011	0.030654
FAM189A2	1.09	2.13	0.003	0.010839
MAPRE3	1.09	2.13	5e-05	0.000277
PAFAH2	1.09	2.12	5e-05	0.000277
ZNF23	1.09	2.12	5e-05	0.000277
ITM2B	1.08	2.12	5e-05	0.000277
AKAP12	1.08	2.12	5e-05	0.000277
DRP2	1.08	2.12	5e-05	0.000277
DDAH1	1.08	2.11	5e-05	0.000277
LDLRAP1	1.08	2.11	5e-05	0.000277
RELB	1.08	2.11	5e-05	0.000277
SEMA4F	1.08	2.11	5e-05	0.000277
ACOT2	1.08	2.11	7e-04	0.002898
MATN3	1.08	2.11	0.002	0.005860
MCFD2	1.08	2.11	5e-05	0.000277
ST3GAL5	1.08	2.11	5e-05	0.000277
PRCP	1.08	2.11	5e-05	0.000277
ARHGAP20	1.08	2.11	0.000	0.000750
C14orf79	1.07	2.10	1e-04	0.000521
LYST	1.07	2.10	5e-05	0.000277
COLEC12	1.07	2.10	5e-05	0.000277
OLFML3	1.07	2.10	0.006	0.016666
KIRREL	1.07	2.10	5e-05	0.000277
OPN3	1.07	2.10	5e-05	0.000277
FKTN	1.07	2.10	5e-05	0.000277
CCDC85A	1.07	2.10	5e-05	0.000277
CLEC1A	1.07	2.09	5e-05	0.000277
PTPRJ	1.07	2.09	5e-05	0.000277
ZNF385A	1.06	2.09	5e-05	0.000277
P4HA3	1.06	2.09	5e-05	0.000277
CTBS	1.06	2.09	5e-05	0.000277
EEF1E1,EEF1E1-MUTED,MUTED,MUTED-TXNDC5,TXNDC5	1.06	2.09	5e-05	0.000277
IKZF4	1.06	2.09	5e-05	0.000277
PFN1P2	1.06	2.09	0.001	0.003073
GEM	1.06	2.09	2e-04	0.000971
EDA2R	1.06	2.09	5e-05	0.000277
SDC4	1.06	2.08	5e-05	0.000277
ATP6V0A1	1.06	2.08	5e-05	0.000277
NELF	1.06	2.08	5e-05	0.000277
GNPTAB	1.06	2.08	5e-05	0.000277
FZD6	1.06	2.08	5e-05	0.000277
ATP6AP2	1.06	2.08	5e-05	0.000277
BST2	1.05	2.08	0.000	0.000750
ECE1	1.05	2.07	5e-05	0.000277
ANKRD5	1.05	2.07	5e-05	0.000277
AIG1	1.05	2.07	5e-05	0.000277
ADAMTSL1	1.05	2.07	5e-05	0.000277
SFXN5	1.04	2.06	5e-05	0.000277
FTL	1.04	2.06	5e-05	0.000277
TMEM88	1.04	2.06	0.002	0.006613
VCL	1.04	2.06	5e-05	0.000277
DNM3OS	1.04	2.05	0.005	0.015525
PFKFB2	1.04	2.05	0.001	0.002358
HKR1	1.03	2.05	5e-05	0.000277
LRRC37A4	1.03	2.05	5e-05	0.000277
SYT3	1.03	2.05	0.002	0.006298
C1orf151-NBL1,MINOS1,NBL1	1.03	2.04	5e-05	0.000277
CTSF	1.03	2.04	5e-05	0.000277
PDCD1LG2	1.03	2.04	5e-05	0.000277
LOC100128252	1.03	2.04	0.001	0.004769
C22orf23	1.03	2.04	0.001	0.003747
TM2D1	1.03	2.04	5e-05	0.000277
SUGT1P1	1.03	2.04	0.007	0.020846
ATP1B1	1.03	2.04	5e-05	0.000277
STC1	1.03	2.04	5e-05	0.000277
ESM1	1.03	2.04	5e-05	0.000277
LRRC16A	1.02	2.03	5e-05	0.000277
PTCHD4	1.02	2.03	5e-05	0.000277
SLC30A4	1.02	2.03	5e-05	0.000277
POFUT2	1.02	2.03	5e-05	0.000277
VCAN	1.02	2.03	5e-05	0.000277
C9orf86	1.02	2.03	5e-05	0.000277
SLC16A4	1.02	2.03	0.002	0.005530
VWCE	1.02	2.03	0.000	0.000750
NID2	1.02	2.03	5e-05	0.000277
CYBRD1	1.02	2.03	5e-05	0.000277
KIF16B	1.02	2.02	5e-05	0.000277
ANTXR2	1.02	2.02	5e-05	0.000277
TUSC2	1.01	2.02	5e-05	0.000277
TGFBR1	1.01	2.02	5e-05	0.000277
APLF	1.01	2.02	0.005	0.015638
STX3	1.01	2.02	5e-05	0.000277
CAMKMT	1.01	2.01	0.006	0.016666
CHCHD7	1.01	2.01	5e-05	0.000277
P4HTM	1.01	2.01	5e-05	0.000277
CTSO	1.01	2.01	5e-05	0.000277
SDC2	1.01	2.01	5e-05	0.000277

Supplementary Table 1 - continued

Gene name	Log2 fold change	Fold change	p-value	q-value
ENTPD5	1.01	2.01	5e-05	0.000277
NAP1L2	1.00	2.01	0.018	0.045510
HRASLS	1.00	2.00	0.016	0.041240
CPT1C	1.00	2.00	5e-05	0.000277
CSRNP1	-1.00	0.50	5e-05	0.000277
FBL	-1.00	0.50	5e-05	0.000277
RTKN	-1.01	0.50	0.000	0.001981
MRPL12	-1.01	0.50	5e-05	0.000277
C14orf49	-1.01	0.50	5e-05	0.000277
FAM216A	-1.01	0.50	0.007	0.019849
FAM161A	-1.01	0.50	0.005	0.016275
TMED10	-1.01	0.50	5e-05	0.000277
GLRX2	-1.01	0.50	2e-04	0.000971
DLL1	-1.01	0.50	5e-05	0.000277
PPP2R3A	-1.01	0.50	5e-05	0.000277
SETMAR	-1.01	0.50	5e-05	0.000277
H1F0	-1.01	0.50	5e-05	0.000277
TTC21B	-1.01	0.50	5e-05	0.000277
PARD6G	-1.01	0.50	0.003	0.010167
CENPL	-1.01	0.50	5e-05	0.000277
C11orf82	-1.01	0.50	2e-04	0.000971
PDLIM1	-1.02	0.49	5e-05	0.000277
FLJ41200	-1.02	0.49	5e-05	0.000277
RAC1	-1.02	0.49	5e-05	0.000277
IER2	-1.02	0.49	5e-05	0.000277
WDR18	-1.02	0.49	5e-05	0.000277
PRKCDBP	-1.02	0.49	5e-05	0.000277
PRIM2	-1.02	0.49	5e-05	0.000277
LCMT2	-1.02	0.49	1e-04	0.000521
ARMC5	-1.02	0.49	5e-05	0.000277
FAM78A	-1.03	0.49	5e-05	0.000277
LPAR6	-1.03	0.49	0.007	0.020117
CENPQ	-1.03	0.49	0.001	0.002358
FAM173A	-1.03	0.49	0.003	0.010600
AKR1C1	-1.03	0.49	0.009	0.025809
C12orf57	-1.03	0.49	5e-05	0.000277
C1orf52	-1.03	0.49	5e-05	0.000277
CCDC85C	-1.03	0.49	0.002	0.006005
SLC39A14	-1.03	0.49	5e-05	0.000277
RPS6KA1	-1.03	0.49	5e-05	0.000277
ENTPD1	-1.03	0.49	4e-04	0.001790
MPZL2	-1.03	0.49	5e-05	0.000277
POLE	-1.03	0.49	5e-05	0.000277
MED6	-1.03	0.49	5e-05	0.000277
RANBP1	-1.04	0.49	5e-05	0.000277
POLR3G	-1.04	0.49	5e-05	0.000277
TMEM117	-1.04	0.49	0.002	0.006906
HNRNPH3	-1.04	0.49	5e-05	0.000277
HMGB1	-1.04	0.49	5e-05	0.000277
EIF1AY	-1.04	0.49	5e-05	0.000277
TTF2	-1.04	0.48	5e-05	0.000277
VASH1	-1.04	0.48	5e-05	0.000277
NCAPD3	-1.05	0.48	5e-05	0.000277
ANP32B	-1.05	0.48	5e-05	0.000277
ARSE	-1.05	0.48	0.002	0.007974
AMPH	-1.05	0.48	5e-05	0.000277
OAS3	-1.05	0.48	5e-05	0.000277
DECR2	-1.05	0.48	5e-05	0.000277
PIM1	-1.05	0.48	0.001	0.005090
BMP4	-1.05	0.48	5e-05	0.000277
C6orf162	-1.05	0.48	0.002	0.006005
RBM42	-1.05	0.48	5e-05	0.000277
FAM117B	-1.05	0.48	5e-05	0.000277
HAUS1	-1.05	0.48	5e-05	0.000277
MIR3615,SLC9A3R1	-1.05	0.48	0.011	0.029402
C15orf23	-1.05	0.48	5e-05	0.000277
TMEM106C	-1.05	0.48	5e-05	0.000277
SLC25A30	-1.05	0.48	5e-05	0.000277
NOL11	-1.05	0.48	5e-05	0.000277
FRAT1	-1.05	0.48	0.005	0.015638
ZNF48	-1.05	0.48	5e-05	0.000277
MGP	-1.05	0.48	5e-05	0.000277
OAS1	-1.05	0.48	0.004	0.011656
GEN1	-1.05	0.48	5e-05	0.000277
CSE1L	-1.06	0.48	5e-05	0.000277
OBSL1	-1.06	0.48	5e-05	0.000277
FAM199X	-1.06	0.48	5e-05	0.000277
MARVELD1	-1.06	0.48	5e-05	0.000277
TGFB1	-1.06	0.48	5e-05	0.000277
SOX4	-1.06	0.48	5e-05	0.000277
MIS18A	-1.06	0.48	5e-05	0.000277
POLD1	-1.06	0.48	5e-05	0.000277
ADAL	-1.06	0.48	0.020	0.049948
NBAS	-1.06	0.48	5e-05	0.000277
DPAGT1	-1.06	0.48	5e-05	0.000277
NUP35	-1.06	0.48	5e-05	0.000277
RAB12	-1.06	0.48	5e-05	0.000277
CXADR	-1.06	0.48	0.004	0.013662
JUNB	-1.06	0.48	5e-05	0.000277
KLHL8	-1.07	0.48	5e-05	0.000277
POLR1B	-1.07	0.48	5e-05	0.000277

Supplementary Table 1 - continued

Gene name	Log2 fold change	Fold change	p-value	q-value
SEMA6C	-1.07	0.48	5e-05	0.000277
GTF2H3	-1.07	0.48	5e-05	0.000277
BTN3A1	-1.07	0.48	5e-05	0.000277
LTBP4	-1.07	0.48	5e-05	0.000277
SHROOM2	-1.07	0.48	5e-05	0.000277
GMPS	-1.07	0.48	5e-05	0.000277
CDK5R1	-1.07	0.48	5e-05	0.000277
MOV10L1	-1.07	0.47	4e-04	0.001790
RAN	-1.08	0.47	5e-05	0.000277
CPNE3	-1.08	0.47	5e-05	0.000277
SLC26A2	-1.08	0.47	5e-05	0.000277
TEX261	-1.08	0.47	5e-05	0.000277
USP28	-1.08	0.47	5e-05	0.000277
SLC2A1	-1.08	0.47	5e-05	0.000277
DAZAP1	-1.08	0.47	5e-05	0.000277
CTNNAL1	-1.08	0.47	5e-05	0.000277
PARVB	-1.08	0.47	5e-05	0.000277
PROX1	-1.08	0.47	5e-05	0.000277
SMO	-1.08	0.47	5e-05	0.000277
POLA1	-1.09	0.47	5e-05	0.000277
TCF7	-1.09	0.47	0.004	0.013036
CCDC61	-1.09	0.47	0.002	0.006005
RNF138	-1.09	0.47	5e-05	0.000277
ATF1	-1.09	0.47	5e-05	0.000277
B4GALNT4	-1.09	0.47	5e-05	0.000277
RYK	-1.09	0.47	5e-05	0.000277
ADAMTS18	-1.09	0.47	5e-05	0.000277
BAIAP2	-1.09	0.47	5e-05	0.000277
FITM2	-1.09	0.47	0.004	0.013520
SLC35E2	-1.10	0.47	5e-05	0.000277
TKT	-1.10	0.47	5e-05	0.000277
NACAD	-1.10	0.47	1e-04	0.000521
ANGPTL2	-1.10	0.47	5e-05	0.000277
ATP5D	-1.10	0.47	5e-05	0.000277
NEDD9	-1.10	0.47	5e-05	0.000277
G3BP1	-1.10	0.47	5e-05	0.000277
SUPT3H	-1.10	0.47	6e-04	0.002551
AZI1	-1.10	0.47	5e-05	0.000277
ECT2	-1.10	0.46	5e-05	0.000277
MSL3P1	-1.11	0.46	0.002	0.008411
TMEM171	-1.11	0.46	5e-05	0.000277
HEY1	-1.11	0.46	5e-05	0.000277
DIS3L	-1.11	0.46	5e-05	0.000277
NRN1	-1.11	0.46	5e-05	0.000277
ASGR1	-1.11	0.46	6e-04	0.002551
PRRC2C	-1.11	0.46	5e-05	0.000277
SPECC1	-1.11	0.46	5e-05	0.000277
BCL7A	-1.11	0.46	5e-05	0.000277
COL12A1	-1.12	0.46	5e-05	0.000277
GP1BB,SEPT5,SEPT5-GP1BB	-1.12	0.46	5e-05	0.000277
NASP	-1.12	0.46	5e-05	0.000277
CECR5	-1.12	0.46	5e-05	0.000277
BHLHE40	-1.13	0.46	5e-05	0.000277
GALK1	-1.13	0.46	5e-05	0.000277
ANKRD32	-1.13	0.46	0.000	0.000750
MTHFD1	-1.13	0.46	5e-05	0.000277
C2CD2	-1.13	0.46	5e-05	0.000277
LXN	-1.13	0.46	0.006	0.017174
IDH2	-1.13	0.46	5e-05	0.000277
MSL3	-1.13	0.46	5e-05	0.000277
DDX39A	-1.13	0.46	5e-05	0.000277
ZNF678	-1.13	0.46	5e-05	0.000277
TGFB2	-1.13	0.46	5e-05	0.000277
SAMD5	-1.13	0.46	5e-05	0.000277
PDCD7	-1.13	0.46	5e-05	0.000277
SEC23B	-1.13	0.46	5e-05	0.000277
COL6A1	-1.13	0.46	5e-05	0.000277
MIR1244-3,PTMA	-1.13	0.46	5e-05	0.000277
CCNG2	-1.13	0.46	5e-05	0.000277
HOMER3	-1.14	0.45	5e-05	0.000277
ZNF512B	-1.14	0.45	5e-05	0.000277
ROBO3	-1.14	0.45	5e-05	0.000277
TMEM106A	-1.14	0.45	8e-04	0.003240
GEMIN5	-1.14	0.45	5e-05	0.000277
TRPV4	-1.14	0.45	5e-05	0.000277
PSMC3IP	-1.14	0.45	0.004	0.013162
AUTS2	-1.14	0.45	5e-05	0.000277
ARRB1	-1.14	0.45	5e-05	0.000277
LAMP1	-1.14	0.45	5e-05	0.000277
GLDC	-1.15	0.45	8e-04	0.003240
PPP1CC	-1.15	0.45	5e-05	0.000277
PGAM1	-1.15	0.45	5e-05	0.000277
HDAC2	-1.15	0.45	5e-05	0.000277
HNRNPH1	-1.15	0.45	5e-05	0.000277
RECQL4	-1.15	0.45	5e-05	0.000277
MALT1	-1.15	0.45	1e-04	0.000521
PHYH	-1.15	0.45	5e-05	0.000277
CCT4	-1.16	0.45	5e-05	0.000277
RBMX	-1.16	0.45	5e-05	0.000277
ELP3	-1.16	0.45	5e-05	0.000277
PYGO1	-1.16	0.45	0.002	0.007530

Supplementary Table 1 - continued

Gene name	Log2 fold change	Fold change	p-value	q-value
EXOSC9	-1.16	0.45	5e-05	0.000277
HNRNPA2B1	-1.16	0.45	5e-05	0.000277
TYRO3	-1.16	0.45	5e-05	0.000277
BMX	-1.16	0.45	5e-05	0.000277
PRMT1	-1.16	0.45	5e-05	0.000277
MRPL3	-1.16	0.45	5e-05	0.000277
C11orf30	-1.16	0.45	5e-05	0.000277
MYPOP	-1.16	0.45	0.000	0.001587
TGFB3	-1.16	0.45	0.002	0.005530
CNTRL	-1.16	0.45	5e-05	0.000277
SNRPA	-1.17	0.45	5e-05	0.000277
FLNC	-1.17	0.45	5e-05	0.000277
HDHD1	-1.17	0.44	5e-05	0.000277
TSPAN13	-1.17	0.44	5e-05	0.000277
SIK1	-1.17	0.44	5e-05	0.000277
LOC338799	-1.17	0.44	0.005	0.015525
UNC5B	-1.17	0.44	3e-04	0.001388
ALMS1	-1.17	0.44	5e-05	0.000277
GAS2L3	-1.17	0.44	5e-05	0.000277
PRKAG2	-1.17	0.44	5e-05	0.000277
CHEK1	-1.17	0.44	5e-05	0.000277
NET1	-1.17	0.44	5e-05	0.000277
FAM102A	-1.18	0.44	5e-05	0.000277
BRD4	-1.18	0.44	5e-05	0.000277
CEP72	-1.18	0.44	0.006	0.019097
CLEC11A	-1.18	0.44	5e-05	0.000277
TMEM160	-1.18	0.44	0.019	0.047216
PDGFB	-1.18	0.44	5e-05	0.000277
MYH10	-1.18	0.44	5e-05	0.000277
ZBTB41	-1.18	0.44	5e-05	0.000277
UNG	-1.18	0.44	5e-05	0.000277
NRGN	-1.18	0.44	5e-05	0.000277
TMEM170A	-1.19	0.44	1e-04	0.000521
DCAF4	-1.19	0.44	0.000	0.000750
ITGB4	-1.19	0.44	7e-04	0.002898
RAP1B	-1.19	0.44	5e-05	0.000277
NUDT1	-1.19	0.44	0.000	0.001981
EEF1A2	-1.19	0.44	5e-05	0.000277
LYL1	-1.19	0.44	5e-05	0.000277
WDR92	-1.19	0.44	5e-05	0.000277
GLYCTK	-1.19	0.44	1e-04	0.000521
C14orf80	-1.19	0.44	5e-05	0.000277
IGFBP5	-1.19	0.44	7e-04	0.002898
TMEM115	-1.20	0.44	5e-05	0.000277
LBH	-1.20	0.44	5e-05	0.000277
PEX26	-1.20	0.44	5e-05	0.000277
LOC729683	-1.20	0.44	0.002	0.006005
LMO4	-1.20	0.43	5e-05	0.000277
FABP4	-1.20	0.43	5e-05	0.000277
NYNRIN	-1.21	0.43	5e-05	0.000277
AHI1	-1.21	0.43	5e-05	0.000277
DYSF	-1.21	0.43	5e-05	0.000277
LHPP	-1.21	0.43	0.004	0.012611
C18orf54	-1.21	0.43	5e-05	0.000277
MIR3917,STMN1	-1.21	0.43	5e-05	0.000277
CEP152	-1.22	0.43	5e-05	0.000277
GLUL	-1.22	0.43	5e-05	0.000277
DDX51	-1.22	0.43	5e-05	0.000277
PTCH1	-1.22	0.43	5e-05	0.000277
AGPAT4	-1.22	0.43	5e-05	0.000277
HAUS8	-1.22	0.43	5e-05	0.000277
H2AFY	-1.22	0.43	5e-05	0.000277
HTR1D	-1.22	0.43	5e-05	0.000277
CHST15	-1.22	0.43	5e-05	0.000277
GPC2	-1.23	0.43	5e-05	0.000277
FKBP11	-1.23	0.43	5e-05	0.000277
ASPHD2	-1.24	0.42	5e-05	0.000277
POLR2A	-1.24	0.42	5e-05	0.000277
TMEM204	-1.24	0.42	2e-04	0.000971
TRDMT1	-1.24	0.42	5e-05	0.000277
MYO1B	-1.24	0.42	5e-05	0.000277
ENOSF1	-1.24	0.42	5e-05	0.000277
SLC17A9	-1.24	0.42	5e-05	0.000277
SAP30	-1.25	0.42	5e-05	0.000277
PAICS	-1.25	0.42	5e-05	0.000277
ETV4	-1.25	0.42	5e-05	0.000277
C16orf59	-1.25	0.42	0.001	0.005385
PGF	-1.25	0.42	5e-05	0.000277
GPX7	-1.25	0.42	0.002	0.007234
NDUFA5	-1.25	0.42	5e-05	0.000277
TOR1A	-1.26	0.42	5e-05	0.000277
KANK3	-1.26	0.42	5e-05	0.000277
TFAM	-1.26	0.42	5e-05	0.000277
MCRS1	-1.26	0.42	5e-05	0.000277
PIM3	-1.26	0.42	5e-05	0.000277
CAMK4	-1.26	0.42	0.002	0.005530
CSRP2	-1.26	0.42	0.013	0.035300
TNFAIP8L1	-1.27	0.42	5e-05	0.000277
HNRPDL	-1.27	0.42	5e-05	0.000277
MIR4647,SLC35B2	-1.27	0.42	5e-05	0.000277
EMP2	-1.27	0.41	5e-05	0.000277

Supplementary Table 1 - continued

Gene name	Log2 fold change	Fold change	p-value	q-value
GBGT1	-1.27	0.41	5e-05	0.000277
HNRNPD	-1.27	0.41	5e-05	0.000277
GATA2	-1.27	0.41	5e-05	0.000277
CERK	-1.27	0.41	5e-05	0.000277
LOC643733	-1.28	0.41	5e-05	0.000277
RNASEH2B	-1.28	0.41	5e-05	0.000277
GABBR2	-1.28	0.41	5e-05	0.000277
PLCD4	-1.28	0.41	5e-05	0.000277
SCAF4	-1.29	0.41	5e-05	0.000277
HOXA5	-1.29	0.41	0.009	0.025688
SOCS3	-1.29	0.41	5e-05	0.000277
HMGB3	-1.29	0.41	5e-05	0.000277
COBRA1	-1.29	0.41	5e-05	0.000277
GPRC5B	-1.29	0.41	5e-05	0.000277
TONSL	-1.30	0.41	5e-05	0.000277
STARD7	-1.30	0.41	5e-05	0.000277
AMIGO2	-1.30	0.41	5e-05	0.000277
NGLY1	-1.30	0.41	5e-05	0.000277
EXOSC3	-1.30	0.41	5e-05	0.000277
NCAPD2	-1.30	0.41	5e-05	0.000277
PEX11A	-1.31	0.40	0.019	0.047016
KLHL23,PHOSPHO2,PHOSPHO2-KLHL23	-1.31	0.40	5e-05	0.000277
ALG10	-1.31	0.40	5e-05	0.000277
SMC2	-1.31	0.40	5e-05	0.000277
MRAS	-1.32	0.40	5e-05	0.000277
FNBP1L	-1.32	0.40	5e-05	0.000277
DDX11	-1.32	0.40	5e-05	0.000277
FAM89A,MIR1182	-1.32	0.40	0.003	0.009007
PWP2	-1.32	0.40	5e-05	0.000277
MYBL1	-1.32	0.40	1e-04	0.000521
MMP17	-1.32	0.40	5e-05	0.000277
SLC35E2B	-1.32	0.40	5e-05	0.000277
NPM1	-1.32	0.40	5e-05	0.000277
ANP32E	-1.32	0.40	5e-05	0.000277
MDK	-1.33	0.40	5e-05	0.000277
ABCA4	-1.33	0.40	5e-05	0.000277
BAHCC1	-1.33	0.40	5e-05	0.000277
CENPJ	-1.33	0.40	5e-05	0.000277
HTR1B	-1.33	0.40	5e-05	0.000277
THSD7A	-1.33	0.40	5e-05	0.000277
VRK1	-1.34	0.40	5e-05	0.000277
MRPL45	-1.34	0.40	5e-05	0.000277
CTDSP1	-1.34	0.40	5e-05	0.000277
RND1	-1.34	0.40	0.004	0.011914
TMEM48	-1.34	0.40	5e-05	0.000277
HAUS4,MIR4707	-1.34	0.39	5e-05	0.000277
WDR67	-1.35	0.39	5e-05	0.000277
CHAF1A	-1.35	0.39	5e-05	0.000277
CDKN2D	-1.35	0.39	0.000	0.001587
HNRNPM	-1.35	0.39	5e-05	0.000277
WBSCR16	-1.35	0.39	5e-05	0.000277
ZNF268	-1.36	0.39	5e-05	0.000277
PAFAH1B3	-1.36	0.39	5e-05	0.000277
RGS4	-1.37	0.39	5e-05	0.000277
STARD4	-1.37	0.39	0.001	0.003916
CRIP2	-1.37	0.39	5e-05	0.000277
USP1	-1.37	0.39	5e-05	0.000277
CREG2	-1.38	0.39	0.000	0.001587
ZNF714	-1.38	0.39	5e-05	0.000277
GRSF1	-1.38	0.39	5e-05	0.000277
LIN9	-1.38	0.38	5e-05	0.000277
PSPH	-1.38	0.38	5e-05	0.000277
C7orf44	-1.38	0.38	5e-05	0.000277
TPGS1	-1.38	0.38	0.004	0.012045
MYCN	-1.38	0.38	3e-04	0.001388
LIG3	-1.38	0.38	5e-05	0.000277
BCL2L11	-1.38	0.38	5e-05	0.000277
FANCG	-1.38	0.38	5e-05	0.000277
TOX	-1.38	0.38	5e-05	0.000277
PRPS2	-1.38	0.38	5e-05	0.000277
RCC1,SNHG3	-1.38	0.38	5e-05	0.000277
EIF4EBP1	-1.38	0.38	5e-05	0.000277
RMI1	-1.38	0.38	5e-05	0.000277
RANBP9	-1.39	0.38	5e-05	0.000277
HOXA3	-1.39	0.38	5e-05	0.000277
CCDC117	-1.39	0.38	5e-05	0.000277
LDHA	-1.39	0.38	5e-05	0.000277
VAPA	-1.39	0.38	5e-05	0.000277
C4orf46	-1.39	0.38	5e-05	0.000277
FBXO21	-1.40	0.38	5e-05	0.000277
GRB14	-1.40	0.38	5e-05	0.000277
SLC35G1	-1.40	0.38	0.006	0.018973
RFC5	-1.40	0.38	5e-05	0.000277
C12orf52	-1.40	0.38	5e-05	0.000277
RNASEH2A	-1.40	0.38	5e-05	0.000277
CTDNEP1	-1.40	0.38	5e-05	0.000277
HSPBP1	-1.41	0.38	5e-05	0.000277
DCXR	-1.41	0.38	5e-05	0.000277
CYP27C1	-1.41	0.38	1e-04	0.000521
GPSM2	-1.41	0.38	0.006	0.018863
EXOSC6	-1.41	0.38	5e-05	0.000277

Supplementary Table 1 - continued

Gene name	Log2 fold change	Fold change	p-value	q-value
ALG10B	-1.41	0.38	5e-05	0.000277
UTP11L	-1.41	0.38	5e-05	0.000277
FUS	-1.41	0.38	5e-05	0.000277
CLDN7	-1.41	0.38	0.001	0.003073
UNC5A	-1.42	0.37	0.001	0.004276
CBR3	-1.42	0.37	0.002	0.005530
NFIX	-1.42	0.37	5e-05	0.000277
TUBA1B	-1.42	0.37	5e-05	0.000277
KRT80	-1.42	0.37	5e-05	0.000277
C4orf21	-1.42	0.37	0.009	0.026311
MT1X	-1.42	0.37	0.009	0.024123
C5orf34	-1.43	0.37	0.002	0.006458
LOC145783	-1.43	0.37	0.002	0.006141
CREB3L2	-1.43	0.37	5e-05	0.000277
NUDT11	-1.43	0.37	5e-05	0.000277
C1orf187	-1.44	0.37	5e-05	0.000277
ISOC1	-1.44	0.37	5e-05	0.000277
CKS2	-1.44	0.37	5e-05	0.000277
PASK	-1.44	0.37	5e-05	0.000277
H2AFZ	-1.44	0.37	5e-05	0.000277
PCNA-AS1	-1.44	0.37	3e-04	0.001388
LRP12	-1.44	0.37	5e-05	0.000277
USP46	-1.45	0.37	5e-05	0.000277
TTYH2	-1.45	0.37	0.000	0.001183
MMP15	-1.45	0.37	5e-05	0.000277
MAF	-1.45	0.37	0.001	0.004097
DHFR	-1.45	0.36	0.010	0.027380
MCM5	-1.46	0.36	5e-05	0.000277
H1FX	-1.46	0.36	5e-05	0.000277
TFAP4	-1.46	0.36	5e-05	0.000277
SLC19A1	-1.46	0.36	5e-05	0.000277
TCF15	-1.46	0.36	0.011	0.030446
SAMD1	-1.46	0.36	5e-05	0.000277
CDC25A	-1.46	0.36	5e-05	0.000277
KNTC1	-1.46	0.36	5e-05	0.000277
SLC29A4	-1.47	0.36	5e-05	0.000277
KRT18	-1.47	0.36	5e-05	0.000277
BAK1	-1.47	0.36	5e-05	0.000277
PCNA	-1.48	0.36	5e-05	0.000277
MIR3134,PTBP3	-1.48	0.36	5e-05	0.000277
ARHGAP39	-1.48	0.36	5e-05	0.000277
SEMA6A	-1.48	0.36	5e-05	0.000277
GGT5	-1.48	0.36	5e-05	0.000277
DBF4B	-1.49	0.36	5e-05	0.000277
H3F3A	-1.49	0.36	5e-05	0.000277
C1orf96	-1.49	0.36	5e-05	0.000277
LYPD1	-1.49	0.36	5e-05	0.000277
HMGN4	-1.49	0.35	5e-05	0.000277
TBX3	-1.50	0.35	5e-05	0.000277
TRAIP	-1.50	0.35	0.002	0.006458
CHRNA1	-1.50	0.35	2e-04	0.000971
PRRX1	-1.50	0.35	5e-05	0.000277
N4BP2	-1.50	0.35	5e-05	0.000277
DKK1	-1.50	0.35	5e-05	0.000277
CCDC138	-1.50	0.35	0.000	0.001981
FANCE	-1.51	0.35	5e-05	0.000277
MSMP	-1.51	0.35	5e-05	0.000277
MCM3	-1.51	0.35	5e-05	0.000277
CACYBP	-1.52	0.35	5e-05	0.000277
TUBB3	-1.52	0.35	5e-05	0.000277
SNAI2	-1.52	0.35	5e-05	0.000277
MCM8	-1.52	0.35	5e-05	0.000277
GNB3	-1.53	0.35	0.001	0.004601
SPSB1	-1.53	0.35	5e-05	0.000277
CDCA4	-1.53	0.35	5e-05	0.000277
PTGR2	-1.53	0.35	0.003	0.010839
EVL	-1.54	0.34	5e-05	0.000277
ZAK	-1.54	0.34	5e-05	0.000277
CLIC3	-1.54	0.34	0.007	0.021219
ARHGAP18	-1.55	0.34	5e-05	0.000277
HES2	-1.55	0.34	5e-05	0.000277
YAP1	-1.55	0.34	5e-05	0.000277
SLFN11	-1.55	0.34	5e-05	0.000277
SHF	-1.55	0.34	0.001	0.005090
UBE2S	-1.55	0.34	5e-05	0.000277
IER5L	-1.55	0.34	6e-04	0.002551
LIMS2	-1.55	0.34	0.000	0.001587
INCENP	-1.56	0.34	5e-05	0.000277
SLC16A5	-1.56	0.34	5e-05	0.000277
LOXL2	-1.56	0.34	5e-05	0.000277
UNC13D	-1.56	0.34	5e-05	0.000277
RAB34	-1.56	0.34	5e-05	0.000277
TRIM59	-1.56	0.34	5e-05	0.000277
CBX6	-1.57	0.34	5e-05	0.000277
MT1L	-1.57	0.34	0.003	0.010988
SPATA6	-1.57	0.34	5e-05	0.000277
ZCCHC24	-1.58	0.33	5e-05	0.000277
EGR1	-1.58	0.33	5e-05	0.000277
RRM1	-1.59	0.33	5e-05	0.000277
DNMT3B	-1.59	0.33	5e-05	0.000277
FBXL16	-1.59	0.33	5e-05	0.000277

Supplementary Table 1 - continued

Gene name	Log2 fold change	Fold change	p-value	q-value
IGFBP6	-1.59	0.33	5e-05	0.000277
BRD3	-1.59	0.33	0.000	0.001183
WDR4	-1.59	0.33	5e-05	0.000277
STAP2	-1.60	0.33	0.001	0.003402
RPA3	-1.60	0.33	0.006	0.017054
CHAF1B	-1.60	0.33	5e-05	0.000277
CMTM6	-1.62	0.33	5e-05	0.000277
FAM174A	-1.62	0.33	5e-05	0.000277
LOC100134868	-1.62	0.33	8e-04	0.003240
RHOD	-1.62	0.33	1e-04	0.000521
CHRNA5	-1.62	0.32	5e-05	0.000277
WDFY2	-1.63	0.32	5e-05	0.000277
FEN1	-1.63	0.32	5e-05	0.000277
AMMECR1	-1.63	0.32	5e-05	0.000277
HES6	-1.64	0.32	0.004	0.013417
CHTF18	-1.65	0.32	5e-05	0.000277
MIR210HG	-1.65	0.32	0.002	0.008261
CHML	-1.65	0.32	0.000	0.001183
DUT	-1.65	0.32	5e-05	0.000277
ZNF367	-1.65	0.32	5e-05	0.000277
STAT5A	-1.65	0.32	5e-05	0.000277
PLDN	-1.66	0.32	5e-05	0.000277
SLC25A10	-1.66	0.32	5e-05	0.000277
DLL4	-1.67	0.31	5e-05	0.000277
GMNN	-1.67	0.31	5e-05	0.000277
PRR11	-1.68	0.31	5e-05	0.000277
PEG10	-1.68	0.31	5e-05	0.000277
ZNF300	-1.68	0.31	5e-05	0.000277
MIS18BP1	-1.68	0.31	5e-05	0.000277
ALDH1A2	-1.68	0.31	5e-05	0.000277
HNRNPA1,HNRNPA1P10	-1.68	0.31	5e-05	0.000277
CCDC34	-1.69	0.31	5e-05	0.000277
FANCI	-1.69	0.31	5e-05	0.000277
B3GALNT2	-1.69	0.31	5e-05	0.000277
MMP14	-1.69	0.31	5e-05	0.000277
ABCB6	-1.69	0.31	5e-05	0.000277
PHGDH	-1.69	0.31	5e-05	0.000277
HMGCS1	-1.69	0.31	5e-05	0.000277
MCM7	-1.70	0.31	5e-05	0.000277
SYNE2	-1.71	0.31	5e-05	0.000277
GPT2	-1.71	0.30	5e-05	0.000277
KIT	-1.72	0.30	5e-05	0.000277
TEAD2	-1.73	0.30	5e-05	0.000277
CKAP2	-1.74	0.30	5e-05	0.000277
MCM2	-1.74	0.30	5e-05	0.000277
CYP26B1	-1.74	0.30	1e-04	0.000521
GALM	-1.75	0.30	0.002	0.005860
DHRS3	-1.75	0.30	5e-05	0.000277
DBF4	-1.75	0.30	5e-05	0.000277
ZNF655	-1.75	0.30	0.000	0.001183
TLL1	-1.75	0.30	5e-05	0.000277
RBL1	-1.76	0.30	5e-05	0.000277
TMEM71	-1.76	0.29	4e-04	0.001790
PC	-1.78	0.29	0.002	0.007381
RAB27A	-1.78	0.29	5e-05	0.000277
BARD1	-1.79	0.29	1e-04	0.000521
C17orf53	-1.79	0.29	5e-05	0.000277
TMEM97	-1.81	0.29	5e-04	0.002168
ABCB7	-1.81	0.28	5e-05	0.000277
CCDC99	-1.82	0.28	5e-05	0.000277
ABCG2	-1.83	0.28	5e-05	0.000277
LOC100288637	-1.85	0.28	5e-05	0.000277
CDKN1C	-1.85	0.28	4e-04	0.001790
BRI3BP	-1.86	0.28	0.001	0.004932
HIC1	-1.86	0.28	5e-05	0.000277
H2AFX	-1.86	0.28	5e-05	0.000277
PARPBP	-1.87	0.27	5e-05	0.000277
HELLS	-1.87	0.27	5e-05	0.000277
C1orf112	-1.88	0.27	1e-04	0.000521
DPYSL3	-1.88	0.27	5e-05	0.000277
SPARC	-1.89	0.27	5e-05	0.000277
WNK4	-1.90	0.27	5e-05	0.000277
MTBP	-1.91	0.27	5e-05	0.000277
TACC3	-1.92	0.26	5e-05	0.000277
HAPLN1	-1.92	0.26	5e-05	0.000277
SHMT1	-1.92	0.26	0.013	0.035084
NCAPG2	-1.92	0.26	5e-05	0.000277
C9orf167	-1.93	0.26	5e-05	0.000277
CDC7	-1.93	0.26	5e-05	0.000277
KIF14	-1.94	0.26	5e-05	0.000277
STIL	-1.94	0.26	5e-05	0.000277
SMC4	-1.96	0.26	5e-05	0.000277
PCDH10	-1.97	0.26	5e-05	0.000277
ELMOD1	-1.97	0.26	5e-05	0.000277
PACSIN3	-1.97	0.26	5e-05	0.000277
LOC100506178	-1.97	0.25	5e-05	0.000277
HRCT1	-1.97	0.25	0.001	0.002718
HOXD9	-1.97	0.25	0.002	0.006005
NTSR1	-1.98	0.25	5e-05	0.000277
WNT9A	-1.98	0.25	0.001	0.004601
FGD4	-1.99	0.25	5e-05	0.000277

Supplementary Table 1 - continued

Gene name	Log2 fold change	Fold change	p-value	q-value
FANCA	-2.00	0.25	5e-05	0.000277
C1orf110	-2.00	0.25	0.015	0.039300
SOX18	-2.00	0.25	5e-05	0.000277
C10orf10	-2.01	0.25	5e-05	0.000277
EBPL	-2.01	0.25	5e-05	0.000277
CENPH	-2.01	0.25	5e-05	0.000277
CXCR7	-2.02	0.25	5e-05	0.000277
TMPO	-2.02	0.25	5e-05	0.000277
CENPW	-2.04	0.24	5e-05	0.000277
MCM6	-2.04	0.24	5e-05	0.000277
IGSF8	-2.04	0.24	5e-05	0.000277
MELK	-2.05	0.24	5e-05	0.000277
FST	-2.06	0.24	0.011	0.028937
CORO1A,LOC606724	-2.06	0.24	5e-05	0.000277
MAP2K6	-2.06	0.24	5e-05	0.000277
DIAPH3	-2.06	0.24	5e-05	0.000277
UCP2	-2.07	0.24	0.000	0.001587
POC1A	-2.09	0.24	5e-05	0.000277
LMO7	-2.10	0.23	5e-05	0.000277
RFC3	-2.10	0.23	5e-05	0.000277
KIF22	-2.10	0.23	5e-05	0.000277
HMGN2	-2.11	0.23	5e-05	0.000277
MIF	-2.11	0.23	5e-05	0.000277
ACTB	-2.11	0.23	5e-05	0.000277
ACE	-2.12	0.23	5e-05	0.000277
LPPR3	-2.12	0.23	5e-04	0.002168
LOC158376	-2.13	0.23	5e-05	0.000277
TYMS	-2.14	0.23	5e-05	0.000277
UBE2T	-2.14	0.23	5e-05	0.000277
TACO1	-2.14	0.23	5e-05	0.000277
TRIP13	-2.16	0.22	5e-05	0.000277
FADS2	-2.16	0.22	5e-05	0.000277
SERTAD4	-2.16	0.22	5e-05	0.000277
DNA2	-2.16	0.22	5e-05	0.000277
HMGB2	-2.17	0.22	5e-05	0.000277
SNTB1	-2.17	0.22	5e-05	0.000277
PRSS12	-2.18	0.22	5e-05	0.000277
CKS1B	-2.18	0.22	5e-05	0.000277
ATAD5	-2.19	0.22	5e-05	0.000277
CHST1	-2.20	0.22	5e-05	0.000277
ESPL1	-2.21	0.22	5e-05	0.000277
CKB	-2.22	0.21	0.001	0.003747
FBXO5	-2.25	0.21	5e-05	0.000277
C19orf33	-2.26	0.21	0.013	0.033969
DDX12P	-2.26	0.21	0.001	0.003916
CENPV	-2.27	0.21	5e-05	0.000277
SLC24A1	-2.27	0.21	5e-05	0.000277
SULT1B1	-2.27	0.21	5e-05	0.000277
SLCO4A1	-2.28	0.21	3e-04	0.001388
NRARP	-2.29	0.20	5e-05	0.000277
AURKA	-2.29	0.20	5e-05	0.000277
LRRC17	-2.30	0.20	0.001	0.004601
C1orf135	-2.32	0.20	0.004	0.013928
KIAA1524	-2.33	0.20	5e-05	0.000277
CENPK	-2.33	0.20	5e-05	0.000277
COL1A2	-2.34	0.20	5e-05	0.000277
FAM54A	-2.36	0.19	1e-04	0.000521
C21orf58	-2.37	0.19	4e-04	0.001790
GDF6	-2.38	0.19	5e-05	0.000277
MCM4	-2.38	0.19	5e-05	0.000277
EDIL3	-2.38	0.19	5e-05	0.000277
SGOL2	-2.38	0.19	5e-05	0.000277
TNR	-2.39	0.19	1e-04	0.000521
CD24	-2.40	0.19	5e-05	0.000277
CLCN4	-2.40	0.19	5e-05	0.000277
GNB4	-2.40	0.19	5e-05	0.000277
SH3GL1	-2.41	0.19	5e-05	0.000277
FAM174B	-2.41	0.19	5e-05	0.000277
RPL22L1	-2.42	0.19	5e-05	0.000277
MYLK2	-2.43	0.19	3e-04	0.001388
CCL14,CCL14-CCL15,CCL15	-2.43	0.19	5e-05	0.000277
GINS4	-2.44	0.18	5e-05	0.000277
SLC7A2	-2.45	0.18	5e-05	0.000277
PLS1	-2.47	0.18	0.000	0.001981
BRCA1	-2.49	0.18	5e-05	0.000277
KIF20B	-2.49	0.18	5e-05	0.000277
CCNA1	-2.49	0.18	5e-05	0.000277
TWIST2	-2.50	0.18	0.003	0.010726
AQP1	-2.53	0.17	5e-04	0.002168
TCF19	-2.57	0.17	0.001	0.005233
PRIM1	-2.60	0.17	5e-05	0.000277
BGN	-2.60	0.16	5e-05	0.000277
CDT1	-2.60	0.16	5e-05	0.000277
GINS2	-2.62	0.16	5e-05	0.000277
ORC6	-2.62	0.16	5e-05	0.000277
RMI2	-2.63	0.16	0.000	0.000750
ACSS1	-2.63	0.16	5e-05	0.000277
UHRF1	-2.64	0.16	5e-05	0.000277
E2F8	-2.65	0.16	5e-05	0.000277
CEP128	-2.66	0.16	5e-05	0.000277
TRIM7	-2.70	0.15	5e-05	0.000277

Supplementary Table 1 - continued

Gene name	Log2 fold change	Fold change	p-value	q-value
LINC00176	-2.70	0.15	5e-05	0.000277
ATAD2	-2.72	0.15	5e-05	0.000277
HAS3	-2.73	0.15	0.012	0.031547
CENPI	-2.74	0.15	5e-05	0.000277
KIF23	-2.75	0.15	5e-05	0.000277
POLE2	-2.76	0.15	5e-05	0.000277
FAM172A,MIR2277	-2.77	0.15	5e-05	0.000277
KRT7	-2.78	0.15	5e-05	0.000277
C2CD4A	-2.79	0.14	5e-05	0.000277
BCL2A1	-2.80	0.14	2e-04	0.000971
KIF26A	-2.81	0.14	5e-05	0.000277
GINS1	-2.82	0.14	5e-05	0.000277
CCNA2	-2.87	0.14	5e-05	0.000277
FSCN1	-2.89	0.14	5e-05	0.000277
CENPE	-2.89	0.13	5e-05	0.000277
FANCD2	-2.91	0.13	5e-05	0.000277
CDCA7	-2.92	0.13	5e-05	0.000277
CDCA5	-2.92	0.13	5e-05	0.000277
BRIP1	-2.93	0.13	5e-05	0.000277
WDR62	-2.93	0.13	5e-05	0.000277
TFF3	-2.93	0.13	5e-05	0.000277
BLM	-2.95	0.13	5e-05	0.000277
PLK4	-2.96	0.13	5e-05	0.000277
GJA4	-2.96	0.13	5e-05	0.000277
KCNN2	-2.97	0.13	0.003	0.011111
RAD51	-2.97	0.13	5e-05	0.000277
MLF1IP	-2.99	0.13	5e-05	0.000277
ARHGAP11A	-3.00	0.12	5e-05	0.000277
EPHB6	-3.00	0.12	5e-05	0.000277
RAD51AP1	-3.01	0.12	5e-05	0.000277
DPF1	-3.02	0.12	0.004	0.011374
MT1E	-3.02	0.12	5e-05	0.000277
POLQ	-3.06	0.12	5e-05	0.000277
WDR76	-3.10	0.12	5e-05	0.000277
CDC6	-3.11	0.12	5e-05	0.000277
PTTG1	-3.12	0.12	5e-05	0.000277
SPC24	-3.15	0.11	0.002	0.006298
BRCA2	-3.16	0.11	5e-05	0.000277
CCNB1	-3.17	0.11	5e-05	0.000277
CIT,MIR1178	-3.22	0.11	0.008	0.022843
GTSE1	-3.26	0.10	5e-05	0.000277
XRCC2	-3.27	0.10	0.001	0.002718
SAPCD2	-3.28	0.10	5e-05	0.000277
FANCB	-3.30	0.10	9e-04	0.003578
TPX2	-3.30	0.10	5e-05	0.000277
DTL	-3.31	0.10	5e-05	0.000277
CENPM	-3.33	0.10	5e-05	0.000277
C15orf42	-3.34	0.10	0.001	0.003747
CKAP2L	-3.35	0.10	5e-05	0.000277
FAM83D	-3.35	0.10	5e-05	0.000277
CCNE2	-3.36	0.10	0.001	0.002358
CENPF	-3.40	0.09	5e-05	0.000277
RACGAP1	-3.44	0.09	5e-05	0.000277
CDC45	-3.44	0.09	5e-05	0.000277
CDCA8	-3.45	0.09	5e-05	0.000277
CDCA3	-3.47	0.09	5e-05	0.000277
ANLN	-3.51	0.09	5e-05	0.000277
DSCC1	-3.53	0.09	0.001	0.003073
MAD2L1	-3.53	0.09	5e-05	0.000277
HJURP	-3.55	0.09	5e-05	0.000277
DEPDC1B	-3.60	0.08	5e-05	0.000277
LMNB1	-3.62	0.08	5e-05	0.000277
NEIL3	-3.64	0.08	0.003	0.010310
KIF18A	-3.64	0.08	5e-05	0.000277
TROAP	-3.66	0.08	1e-04	0.000521
ZWINT	-3.67	0.08	5e-05	0.000277
CDKN3	-3.68	0.08	5e-05	0.000277
SHCBP1	-3.69	0.08	5e-05	0.000277
SPAG5	-3.70	0.08	5e-05	0.000277
ASF1B	-3.71	0.08	5e-05	0.000277
IQGAP3	-3.72	0.08	5e-05	0.000277
CEP55	-3.72	0.08	5e-05	0.000277
CDK1	-3.74	0.07	5e-05	0.000277
ERCC6L	-3.74	0.07	0.009	0.025446
CDKN2C	-3.75	0.07	0.001	0.004769
EFNA2	-3.77	0.07	0.003	0.009605
KIF4A	-3.82	0.07	5e-05	0.000277
PKMYT1	-3.83	0.07	0.000	0.001183
CENPA	-3.85	0.07	1e-04	0.000521
KIAA0101	-3.90	0.07	5e-05	0.000277
DLGAP5	-3.92	0.07	5e-05	0.000277
DEPDC1	-3.94	0.07	5e-05	0.000277
FAM64A	-3.94	0.07	5e-05	0.000277
KIF11	-4.01	0.06	5e-05	0.000277
ORC1	-4.01	0.06	5e-05	0.000277
CASC5	-4.03	0.06	5e-05	0.000277
MYBL2	-4.06	0.06	5e-05	0.000277
CLSPN	-4.06	0.06	5e-05	0.000277
TTK	-4.09	0.06	5e-05	0.000277
TK1	-4.10	0.06	5e-05	0.000277
UBE2C	-4.11	0.06	5e-05	0.000277

Supplementary Table 1 - continued

Gene name	Log2 fold change	Fold change	p-value	q-value
CDCA2	-4.11	0.06	5e-05	0.000277
PRC1	-4.12	0.06	5e-05	0.000277
SPC25	-4.16	0.06	1e-04	0.000521
FOXM1	-4.16	0.06	5e-05	0.000277
NDC80	-4.16	0.06	5e-05	0.000277
IGF2,INS,INS-IGF2	-4.17	0.06	5e-05	0.000277
EXO1	-4.17	0.06	5e-05	0.000277
FAM111B	-4.20	0.05	5e-05	0.000277
ESCO2	-4.23	0.05	0.003	0.009007
OIP5	-4.24	0.05	0.018	0.044591
CCNB2	-4.37	0.05	5e-05	0.000277
AURKB	-4.39	0.05	5e-05	0.000277
KIF18B	-4.45	0.05	5e-05	0.000277
TOP2A	-4.48	0.04	5e-05	0.000277
EZH2	-4.50	0.04	5e-05	0.000277
SGOL1	-4.51	0.04	5e-05	0.000277
SKA3	-4.52	0.04	0.002	0.005860
SKA1	-4.53	0.04	5e-05	0.000277
NCAPG	-4.59	0.04	1e-04	0.000521
NUSAP1	-4.59	0.04	5e-05	0.000277
MKI67	-4.62	0.04	5e-05	0.000277
KIF2C	-4.66	0.04	5e-05	0.000277
KIF20A	-4.67	0.04	0.003	0.009731
NUF2	-4.68	0.04	5e-05	0.000277
RRM2	-4.69	0.04	5e-05	0.000277
CDC20	-4.77	0.04	5e-05	0.000277
PLK1	-4.80	0.04	5e-05	0.000277
BUB1B,PAK6	-4.82	0.04	5e-05	0.000277
BUB1	-4.83	0.04	5e-05	0.000277
ASPM	-4.84	0.03	5e-05	0.000277
E2F1	-4.93	0.03	5e-05	0.000277
HMMR	-4.99	0.03	5e-05	0.000277
BIRC5	-5.19	0.03	5e-05	0.000277
NEK2	-5.21	0.03	5e-05	0.000277
NCAPH	-5.27	0.03	5e-05	0.000277
PBK	-5.72	0.02	1e-04	0.000521

*PCDHA1,PCDHA10,PCDHA11,PCDHA12,PCDHA13,PCDHA2,PCDHA3,PCDHA4,PCDHA5,PCDHA6,PCDHA7,
PCDHA8,PCDHA9,PCDHAC1,PCDHAC2

Supplementary Table 2. Biological Process Gene Ontology terms that were significantly overrepresented in the analysis of the list of genes upregulated 2 times or more by EZH-depletion.

Biological Process	GO number	Over- (+) or underrepresented (-)	p-value
Unclassified	-	-	1.77E-10
cell adhesion	GO:0007155	+	2.25E-09
biological adhesion	GO:0022610	+	2.25E-09
immune system process	GO:0002376	+	2.07E-08
response to stimulus	GO:0050896	+	5.94E-07
cellular process	GO:0009987	+	6.88E-06
RNA metabolic process	GO:0016070	-	2.14E-05
immune response	GO:0006955	+	4.65E-05
response to external stimulus	GO:0009605	+	1.12E-04
developmental process	GO:0032502	+	2.13E-04
nucleobase-containing compound metabolic process	GO:0006139	-	4.83E-04
blood coagulation	GO:0007596	+	8.14E-04
macrophage activation	GO:0042116	+	8.24E-04
cell-matrix adhesion	GO:0007160	+	3.15E-03
cell communication	GO:0007154	+	3.42E-03
cellular defense response	GO:0006968	+	6.92E-03
cell-cell adhesion	GO:0016337	+	9.43E-03
transcription, DNA-dependent	GO:0006351	-	9.68E-03
transcription from RNA polymerase II promoter	GO:0006366	-	1.12E-02
anatomical structure morphogenesis	GO:0009653	+	1.22E-02
system development	GO:0048731	+	2.31E-02
mRNA splicing, via spliceosome	GO:0000398	-	3.77E-02
carbohydrate metabolic process	GO:0005975	+	4.59E-02

Supplementary Table 3. Biological Process Gene Ontology terms that were significantly overrepresented in the analysis of the list of genes downregulated 2 times or more by EZH-depletion.

Biological Process	GO number	Over- (+) or underrepresented (-)	p-value
cell cycle	GO:0007049	+	1.60E-26
DNA metabolic process	GO:0006259	+	3.40E-18
DNA replication	GO:0006260	+	3.87E-17
cellular process	GO:0009987	+	1.02E-13
nucleobase-containing compound metabolic process	GO:0006139	+	9.75E-12
mitosis	GO:0007067	+	1.94E-10
chromosome segregation	GO:0007059	+	8.98E-10
Unclassified	-	-	3.37E-09
metabolic process	GO:0008152	+	3.03E-07
chromatin organization	GO:0006325	+	6.34E-07
cellular component organization	GO:0016043	+	2.24E-06
DNA repair	GO:0006281	+	6.05E-06
cellular component organization or biogenesis	GO:0071840	+	1.30E-05
cytokinesis	GO:0000910	+	1.69E-05
organelle organization	GO:0006996	+	1.92E-05
primary metabolic process	GO:0044238	+	1.34E-04
immune response	GO:0006955	-	2.74E-02

Supplementary Table 4. Genes up- or down-regulated 2 times or more by the exposure of HUVEC to FSS.

Gene name	Log2 fold change	Fold change	p-value	q-value
IGFBP5	7.86	232.18	5e-05	0.000214
ASS1	7.65	200.81	0.011	0.026357
CMKLR1	7.58	191.91	5e-05	0.000214
ITGB4	7.32	159.51	5e-05	0.000214
NOV	7.10	137.12	5e-05	0.000214
FBLN2	6.98	126.32	5e-05	0.000214
MYOM3	6.51	91.29	0.003	0.007650
KCNK3	5.75	53.90	5e-05	0.000214
KCNN4	5.25	37.94	0.003	0.007650
ADAMTS1	5.02	32.35	5e-05	0.000214
FAM3D	4.97	31.31	5e-05	0.000214
STC1	4.94	30.73	5e-05	0.000214
L1CAM	4.91	30.03	0.015	0.035822
TXNIP	4.91	29.98	5e-05	0.000214
KLF4	4.84	28.62	5e-05	0.000214
FGF18	4.76	27.15	0.018	0.040506
HAVCR2	4.42	21.47	0.003	0.007650
VCAN	4.26	19.12	5e-05	0.000214
OLFML2A	4.24	18.87	5e-05	0.000214
CXXC5	4.16	17.88	1e-04	0.000410
PPP1R3C	4.10	17.13	5e-05	0.000214
HPSE	4.08	16.86	5e-05	0.000214
CRISPLD2	3.84	14.34	5e-05	0.000214
NTN1	3.84	14.32	0.003	0.008038
NPR1	3.77	13.64	5e-05	0.000214
LHX6	3.74	13.36	5e-05	0.000214
GDF3	3.73	13.31	5e-05	0.000214
PTHLH	3.69	12.91	5e-05	0.000214
NGEF	3.68	12.79	5e-05	0.000214
SEMA7A	3.66	12.65	5e-05	0.000214
THBD	3.65	12.51	5e-05	0.000214
IL17RE	3.60	12.11	0.006	0.016404
ABCC3	3.57	11.89	0.003	0.008907
EPS8L2	3.52	11.45	5e-05	0.000214
ECM1	3.46	11.02	5e-05	0.000214
PTGS1	3.45	10.95	5e-05	0.000214
APLNR	3.38	10.42	5e-05	0.000214
COL17A1,MIR936	3.38	10.40	5e-05	0.000214
ST6GALNAC1	3.33	10.09	5e-05	0.000214
HS3ST1	3.31	9.91	5e-05	0.000214
ST8SIA6	3.25	9.54	5e-05	0.000214
FAM167B	3.24	9.48	0.001	0.002841
TMEM38A	3.24	9.45	0.001	0.003132
APOL1	3.21	9.27	5e-05	0.000214
LINC00520	3.21	9.24	5e-05	0.000214
C15orf26	3.15	8.90	0.006	0.014902
TMEM229B	3.15	8.86	0.008	0.020483
AQP1	3.14	8.80	5e-05	0.000214
CCDC69	3.13	8.74	5e-05	0.000214
GPR68	3.13	8.73	5e-05	0.000214
LINGO1	3.12	8.70	0.006	0.016879
HSPA12B	3.11	8.63	5e-05	0.000214
MYO7A	3.10	8.58	5e-05	0.000214
AQP3	3.09	8.52	0.001	0.002841
CLIC2	3.05	8.29	5e-05	0.000214
KCNJ12,KCNJ18	3.05	8.28	5e-05	0.000214
CFP	3.03	8.16	5e-05	0.000214
ABI3BP	3.02	8.09	5e-05	0.000214
EXOC3L2	3.00	8.00	5e-05	0.000214
KLF2	2.98	7.91	5e-05	0.000214
ANO1	2.97	7.85	0.004	0.011374
DHH	2.97	7.84	5e-05	0.000214
CRTAC1	2.96	7.80	5e-05	0.000214

Supplementary Table 4 - continued

Gene name	Log2 fold change	Fold change	p-value	q-value
SLC37A2	2.96	7.77	5e-05	0.000214
ADC	2.95	7.72	2e-04	0.000770
CDCP1	2.91	7.52	5e-05	0.000214
RAMP2	2.91	7.49	5e-05	0.000214
C20orf160	2.89	7.41	5e-05	0.000214
FCER1G	2.89	7.41	0.003	0.008907
ZNF488	2.88	7.35	0.002	0.004986
SLC9A3R2	2.87	7.33	5e-05	0.000214
GCH1	2.87	7.32	5e-05	0.000214
HEG1	2.87	7.30	5e-05	0.000214
CXCR7	2.84	7.17	5e-05	0.000214
SULF1	2.80	6.98	5e-05	0.000214
TTC9	2.77	6.81	3e-04	0.001114
MN1	2.76	6.79	5e-05	0.000214
FAM189A2	2.76	6.76	5e-05	0.000214
LIMS2	2.75	6.73	5e-05	0.000214
MAF	2.75	6.72	5e-05	0.000214
COL1A2	2.74	6.70	5e-05	0.000214
ACSL5	2.72	6.61	5e-05	0.000214
PAPPA	2.72	6.57	5e-05	0.000214
UCP2	2.71	6.56	5e-05	0.000214
LYNX1	2.67	6.37	5e-05	0.000214
CTSH	2.67	6.36	5e-05	0.000214
PPAP2B	2.66	6.34	5e-05	0.000214
FLRT3	2.66	6.30	5e-05	0.000214
ENKUR	2.63	6.18	0.001	0.002241
GUCY1A3	2.62	6.16	5e-05	0.000214
LAMB3,MIR4260	2.62	6.14	5e-05	0.000214
NDRG4	2.62	6.14	5e-05	0.000214
KLF11	2.62	6.13	5e-05	0.000214
KCNN3	2.61	6.11	5e-05	0.000214
NDRG1	2.58	5.97	5e-05	0.000214
PRICKLE4	2.58	5.97	5e-05	0.000214
ACP5	2.58	5.96	5e-05	0.000214
LOC151009,LOC440894	2.57	5.93	5e-05	0.000214
KIAA1522	2.56	5.92	5e-05	0.000214
MALL	2.55	5.84	5e-05	0.000214
LOC100190938	2.51	5.70	0.000	0.000595
FAM46C	2.51	5.69	5e-05	0.000214
LMO7	2.50	5.66	5e-05	0.000214
PLAC9	2.50	5.65	5e-05	0.000214
SLC7A8	2.49	5.60	5e-05	0.000214
GNA14	2.47	5.53	0.018	0.040506
PMP22	2.47	5.53	5e-05	0.000214
TSC22D3	2.47	5.52	5e-05	0.000214
ADAMTS9	2.45	5.48	5e-05	0.000214
ADAM19	2.43	5.37	5e-05	0.000214
TMEM158	2.42	5.37	5e-05	0.000214
GJC2	2.42	5.34	0.019	0.042287
GPER	2.41	5.32	5e-05	0.000214
CSMD2	2.40	5.29	5e-05	0.000214
LGALS9	2.40	5.29	5e-05	0.000214
SLCO4A1	2.40	5.29	5e-05	0.000214
OAF	2.39	5.22	5e-05	0.000214
SELP	2.38	5.19	5e-05	0.000214
LMCD1	2.37	5.17	5e-05	0.000214
SCUBE1	2.37	5.16	5e-05	0.000214
KCNMA1	2.36	5.14	5e-05	0.000214
ZNF467	2.36	5.14	5e-05	0.000214
HMOX1	2.36	5.13	5e-05	0.000214
IL1R1	2.36	5.12	5e-05	0.000214
TEK	2.35	5.08	5e-05	0.000214
LINC00341	2.34	5.06	5e-05	0.000214

Supplementary Table 4 - continued

Gene name	Log2 fold change	Fold change	p-value	q-value
MPZL3	2.34	5.05	5e-05	0.000214
GNAZ	2.33	5.04	5e-05	0.000214
ADCY4	2.33	5.02	5e-05	0.000214
BMPER	2.32	5.00	5e-05	0.000214
OAS2	2.32	5.00	5e-05	0.000214
ENTPD1	2.31	4.97	5e-05	0.000214
GPR153	2.31	4.96	5e-05	0.000214
MEF2A	2.31	4.95	5e-05	0.000214
NIPAL3	2.30	4.94	5e-05	0.000214
ITGA10	2.30	4.93	5e-05	0.000214
AKR1C1	2.30	4.92	5e-05	0.000214
GPX3	2.30	4.92	0.000	0.000595
MAMDC2	2.30	4.91	5e-05	0.000214
CALHM2	2.29	4.91	5e-05	0.000214
LOC100507050	2.29	4.90	0.007	0.017106
SLC16A2	2.29	4.89	5e-05	0.000214
SERPINE2	2.29	4.89	5e-05	0.000214
CCIN	2.28	4.86	0.013	0.030164
ANO4	2.26	4.78	5e-05	0.000214
TUBA4A	2.25	4.75	5e-05	0.000214
SEMA6A	2.25	4.74	5e-05	0.000214
AGPAT9	2.24	4.74	5e-05	0.000214
AGAP11,C10orf116	2.24	4.72	5e-05	0.000214
PLVAP	2.24	4.71	5e-05	0.000214
ESPN	2.23	4.71	5e-05	0.000214
TNFRSF19	2.23	4.69	5e-05	0.000214
MATN2	2.23	4.68	0.000	0.000595
MAFB	2.22	4.65	5e-05	0.000214
IGFBP3	2.22	4.64	5e-05	0.000214
ATG9B	2.21	4.62	0.001	0.003278
SOX2	2.20	4.59	0.012	0.029032
TBC1D2	2.19	4.57	5e-05	0.000214
RNF125	2.19	4.56	5e-05	0.000214
ADAMTS4	2.19	4.55	5e-05	0.000214
ADAMTS18	2.18	4.52	5e-05	0.000214
IFNE	2.17	4.49	0.020	0.045326
NKX3-1	2.16	4.47	5e-05	0.000214
EBF1	2.16	4.47	5e-05	0.000214
EMP3	2.15	4.45	5e-05	0.000214
OBFC1	2.15	4.44	5e-05	0.000214
ITGA8	2.13	4.39	0.003	0.008796
SYNPO	2.13	4.38	5e-05	0.000214
APOLD1	2.13	4.36	5e-05	0.000214
GM2A	2.12	4.34	5e-05	0.000214
DLL1	2.11	4.30	5e-05	0.000214
ATP8B2	2.10	4.30	5e-05	0.000214
CYP27C1	2.09	4.24	5e-05	0.000214
CYP1A1	2.08	4.22	5e-05	0.000214
SIGIRR	2.07	4.20	5e-05	0.000214
GPR162	2.07	4.20	5e-05	0.000214
RAPGEF5	2.07	4.19	5e-05	0.000214
CSF2RB	2.07	4.19	5e-05	0.000214
DUSP5	2.07	4.19	5e-05	0.000214
LYST	2.06	4.18	5e-05	0.000214
PLEK2	2.05	4.13	5e-05	0.000214
KCNMB1	2.05	4.13	0.007	0.018036
SEMA4B	2.04	4.12	5e-05	0.000214
ARHGAP6	2.04	4.11	5e-05	0.000214
CPE	2.04	4.10	5e-05	0.000214
OAS1	2.03	4.09	5e-05	0.000214
TNS1	2.03	4.09	5e-05	0.000214
NOS3	2.03	4.09	5e-05	0.000214
MFI2	2.02	4.06	0.001	0.003841

Supplementary Table 4 - continued

Gene name	Log2 fold change	Fold change	p-value	q-value
GCKR	2.02	4.05	5e-05	0.000214
EPB49	2.01	4.04	5e-05	0.000214
ARL4D	2.01	4.03	5e-05	0.000214
SYNE1	2.00	4.00	5e-05	0.000214
CD68	2.00	4.00	5e-05	0.000214
CMAHP	1.99	3.98	0.005	0.014310
SCN1B	1.99	3.96	5e-05	0.000214
SMAD7	1.99	3.96	5e-05	0.000214
PPARGC1B	1.98	3.96	5e-05	0.000214
F2RL3	1.98	3.93	5e-05	0.000214
STARD8	1.98	3.93	5e-05	0.000214
PODXL	1.97	3.91	5e-05	0.000214
FXYD2,FXYD6,FXYD6-FXYD2	1.96	3.90	1e-04	0.000410
NPTX1	1.96	3.89	5e-05	0.000214
FBN1	1.94	3.85	5e-05	0.000214
CALCRL	1.94	3.84	5e-05	0.000214
DNASE1L1	1.94	3.84	1e-04	0.000410
MBP	1.93	3.82	5e-05	0.000214
PLCD3	1.93	3.81	5e-05	0.000214
ITPR3	1.92	3.78	5e-05	0.000214
IL15	1.91	3.75	5e-05	0.000214
ANGPTL4	1.90	3.74	5e-05	0.000214
LOC644242	1.90	3.74	5e-05	0.000214
CORO6	1.90	3.73	0.016	0.037080
COL4A6	1.90	3.73	5e-05	0.000214
PHOSPHO1	1.89	3.72	4e-04	0.001444
FGFR3	1.89	3.71	5e-05	0.000214
LRRK2	1.88	3.69	5e-05	0.000214
SLC27A3	1.88	3.69	5e-05	0.000214
SLIT2	1.88	3.68	5e-05	0.000214
TNS3	1.87	3.66	5e-05	0.000214
HYAL2	1.86	3.64	5e-05	0.000214
ZMIZ1	1.86	3.62	5e-05	0.000214
BFSP1	1.85	3.60	5e-05	0.000214
MRAS	1.84	3.58	5e-05	0.000214
APOL6	1.83	3.56	5e-05	0.000214
DDIT4	1.83	3.55	5e-05	0.000214
PFN1P2	1.82	3.54	5e-05	0.000214
SCN9A	1.82	3.52	5e-05	0.000214
FIGNL2	1.81	3.51	5e-05	0.000214
EFR3B	1.81	3.51	5e-05	0.000214
FAM107A	1.80	3.49	5e-05	0.000214
IL18BP	1.80	3.48	0.017	0.040234
HTR1D	1.80	3.48	5e-05	0.000214
SEMA3F	1.80	3.48	5e-05	0.000214
CA5B	1.80	3.47	5e-05	0.000214
AP1G2,JPH4	1.79	3.47	5e-05	0.000214
C2orf55	1.79	3.46	6e-04	0.002086
ISG20	1.79	3.46	5e-05	0.000214
S100A4	1.79	3.45	0.006	0.015224
CDON	1.79	3.45	5e-05	0.000214
DMRTA1	1.78	3.44	5e-05	0.000214
SERPINB2	1.78	3.44	5e-05	0.000214
ALS2CL	1.78	3.42	5e-05	0.000214
ZNF589	1.77	3.42	5e-05	0.000214
AHNAK2	1.77	3.42	5e-05	0.000214
KIAA1644	1.76	3.38	5e-05	0.000214
PALM	1.75	3.37	5e-05	0.000214
C14orf49	1.75	3.36	5e-05	0.000214
KIAA1199	1.74	3.35	5e-05	0.000214
KITLG	1.74	3.33	5e-05	0.000214
DNM3	1.74	3.33	5e-05	0.000214
PLA2R1	1.73	3.32	5e-05	0.000214

Supplementary Table 4 - continued

Gene name	Log2 fold change	Fold change	p-value	q-value
SLCO3A1	1.73	3.31	5e-05	0.000214
PCDH12	1.73	3.31	5e-05	0.000214
LAPTM5	1.72	3.29	5e-05	0.000214
IL11	1.71	3.27	5e-05	0.000214
SLC17A5	1.71	3.27	5e-05	0.000214
GATSL3	1.70	3.25	5e-05	0.000214
ICAM1	1.70	3.24	5e-05	0.000214
GEM	1.70	3.24	5e-05	0.000214
PPAP2A	1.69	3.24	5e-05	0.000214
NFKBIA	1.69	3.23	5e-05	0.000214
PLAT	1.69	3.23	5e-05	0.000214
CASKIN2	1.68	3.20	5e-05	0.000214
IFI27	1.68	3.20	5e-05	0.000214
ZNF185	1.67	3.19	5e-05	0.000214
FAM22A	1.67	3.18	5e-05	0.000214
OSGIN1	1.67	3.18	5e-05	0.000214
SLC35E4	1.67	3.18	5e-05	0.000214
SSC5D	1.66	3.16	1e-04	0.000410
RASA4	1.65	3.15	5e-05	0.000214
ENDOD1	1.65	3.14	5e-05	0.000214
RASSF9	1.65	3.14	5e-05	0.000214
TESC	1.65	3.13	0.012	0.030075
PLEKHO2	1.65	3.13	5e-05	0.000214
C9orf3	1.64	3.12	5e-05	0.000214
RNASE1	1.64	3.12	5e-05	0.000214
HIST1H2AC	1.64	3.11	5e-05	0.000214
SLC6A6	1.63	3.10	5e-05	0.000214
TSC22D1	1.63	3.09	5e-05	0.000214
TRNP1	1.63	3.09	5e-05	0.000214
CCRL2	1.63	3.09	5e-05	0.000214
ADCY7	1.62	3.07	5e-05	0.000214
ADAM15	1.62	3.07	5e-05	0.000214
C10orf128	1.60	3.04	0.001	0.004564
CLDN15	1.60	3.03	5e-05	0.000214
SFR1	1.59	3.02	5e-05	0.000214
S1PR4	1.59	3.02	0.009	0.021957
PTPRB	1.59	3.01	5e-05	0.000214
SOX13	1.59	3.01	5e-05	0.000214
IL32	1.59	3.00	5e-05	0.000214
CDC25B	1.59	3.00	5e-05	0.000214
QPCT	1.58	3.00	5e-05	0.000214
NUDT7	1.58	2.99	0.003	0.008290
KIF19	1.58	2.99	4e-04	0.001444
PLEKHA6	1.57	2.98	5e-05	0.000214
DIXDC1	1.57	2.97	5e-05	0.000214
NT5E	1.57	2.96	5e-05	0.000214
HIST1H2BK	1.56	2.96	5e-05	0.000214
ADPRH	1.56	2.95	0.001	0.002546
MPZL2	1.56	2.95	5e-05	0.000214
ITGA3	1.55	2.93	5e-05	0.000214
DGKA	1.55	2.93	5e-05	0.000214
PLXDC2	1.55	2.92	5e-05	0.000214
ST8SIA2	1.55	2.92	5e-05	0.000214
PTK2B	1.54	2.90	5e-05	0.000214
MTMR11	1.53	2.89	5e-05	0.000214
MAPK13	1.53	2.89	5e-05	0.000214
WDR66	1.53	2.89	0.004	0.010890
TMBIM1	1.53	2.88	5e-05	0.000214
ERMP1	1.52	2.88	5e-05	0.000214
APBA1	1.52	2.87	5e-05	0.000214
SCUBE3	1.52	2.87	5e-05	0.000214
ATP6V1D	1.51	2.84	5e-05	0.000214
SERPINI1	1.51	2.84	0.017	0.039753

Supplementary Table 4 - continued

Gene name	Log2 fold change	Fold change	p-value	q-value
RAB3C	1.51	2.84	0.005	0.013251
ZC3H6	1.50	2.84	5e-05	0.000214
ARHGAP8,PRR5,PRR5-ARHGAP8	1.50	2.83	5e-05	0.000214
TNFRSF11A	1.50	2.82	5e-05	0.000214
KLF9	1.49	2.81	5e-05	0.000214
TBXA2R	1.49	2.80	5e-05	0.000214
SH3PXD2A	1.48	2.79	5e-05	0.000214
TCN2	1.48	2.79	5e-05	0.000214
NQO1	1.47	2.77	5e-05	0.000214
LOC399744	1.47	2.77	0.000	0.000946
LFNG,MIR4648	1.47	2.77	5e-05	0.000214
CAMKK1	1.46	2.75	5e-05	0.000214
ARHGEF3	1.46	2.75	5e-05	0.000214
C13orf33	1.45	2.74	5e-05	0.000214
ANKRD33B	1.45	2.73	5e-05	0.000214
CD58	1.45	2.73	5e-05	0.000214
ORAI3	1.45	2.73	5e-05	0.000214
C1orf201	1.44	2.72	5e-05	0.000214
LNX1	1.44	2.71	0.002	0.004846
C7orf41	1.44	2.71	5e-05	0.000214
NPC1	1.43	2.70	5e-05	0.000214
TFEB	1.43	2.70	5e-05	0.000214
SLC2A3	1.43	2.69	5e-05	0.000214
PRR5L	1.43	2.69	5e-05	0.000214
ALDH1A2	1.43	2.69	5e-05	0.000214
CYBRD1	1.42	2.68	5e-05	0.000214
TMEM173	1.42	2.68	5e-05	0.000214
ADAM12	1.42	2.67	5e-05	0.000214
SPTBN5	1.42	2.67	5e-05	0.000214
PCDH7	1.41	2.67	5e-05	0.000214
RENBP	1.41	2.66	0.000	0.000595
SLC48A1	1.41	2.66	0.004	0.011006
PROX1	1.41	2.66	5e-05	0.000214
PRICKLE1	1.41	2.65	5e-05	0.000214
PTGR1	1.40	2.64	5e-05	0.000214
BAALC	1.40	2.64	0.003	0.009653
SLC16A4	1.40	2.63	5e-05	0.000214
PIK3C2B	1.39	2.63	5e-05	0.000214
SLK	1.39	2.63	5e-05	0.000214
EPHB1	1.39	2.62	5e-05	0.000214
ZNF790	1.39	2.62	0.001	0.001933
GLIS3	1.39	2.62	5e-05	0.000214
PMEPA1	1.38	2.61	5e-05	0.000214
GFOD1	1.38	2.60	5e-05	0.000214
C14orf43	1.37	2.59	5e-05	0.000214
LOC728431	1.37	2.59	0.009	0.022413
SHISA2	1.37	2.59	5e-05	0.000214
EPHA4	1.37	2.59	5e-05	0.000214
MOBP	1.37	2.59	6e-04	0.002086
GGT5	1.37	2.58	5e-05	0.000214
DSTNP2	1.37	2.58	5e-05	0.000214
EPB41L1	1.37	2.58	5e-05	0.000214
SAMD9	1.37	2.58	5e-05	0.000214
CITED4	1.36	2.57	0.009	0.023372
VSIG10L	1.36	2.57	0.004	0.011866
TIMP1	1.36	2.56	5e-05	0.000214
SQRDL	1.35	2.56	5e-05	0.000214
PARP15	1.35	2.55	0.016	0.036467
TM6SF1	1.35	2.55	5e-05	0.000214
IQCE	1.35	2.54	5e-05	0.000214
DUSP4	1.35	2.54	5e-05	0.000214
TRIM47	1.34	2.54	5e-05	0.000214
CD109	1.34	2.54	5e-05	0.000214

Supplementary Table 4 - continued

Gene name	Log2 fold change	Fold change	p-value	q-value
C7orf10	1.34	2.53	0.003	0.008796
HSPB8	1.34	2.53	5e-05	0.000214
ABLIM3	1.34	2.53	5e-05	0.000214
LGALS3	1.34	2.53	5e-05	0.000214
ARL4C	1.34	2.53	5e-05	0.000214
IER5	1.34	2.52	5e-05	0.000214
HTRA1	1.33	2.52	5e-05	0.000214
FERMT1	1.33	2.52	5e-05	0.000214
B3GNT8	1.33	2.52	5e-05	0.000214
NCKAP5	1.33	2.52	5e-05	0.000214
MIR4315-2,PLEKHM1	1.33	2.51	0.002	0.005918
PLCXD3	1.33	2.51	5e-05	0.000214
PLEC	1.33	2.51	5e-05	0.000214
APOL3	1.33	2.51	5e-05	0.000214
FBXW7	1.32	2.50	5e-05	0.000214
RAPGEF4	1.32	2.50	5e-05	0.000214
RALGAPA2	1.32	2.49	5e-05	0.000214
LGALSL	1.32	2.49	5e-05	0.000214
LURAP1L	1.31	2.48	5e-05	0.000214
RIN1	1.31	2.48	5e-05	0.000214
ZNF347	1.31	2.48	5e-05	0.000214
LRP4	1.31	2.48	5e-05	0.000214
TMC7	1.31	2.48	5e-05	0.000214
SYNJ2	1.31	2.48	5e-05	0.000214
PDK4	1.31	2.47	5e-05	0.000214
RILP	1.31	2.47	5e-05	0.000214
GCHFR	1.31	2.47	1e-04	0.000410
SLC24A6	1.30	2.47	5e-05	0.000214
MIR31HG	1.30	2.47	5e-05	0.000214
FNIP2	1.30	2.46	5e-05	0.000214
LRRC33	1.30	2.46	5e-05	0.000214
FUCA1	1.30	2.46	5e-05	0.000214
TLR1	1.30	2.46	5e-05	0.000214
FAS	1.30	2.46	5e-05	0.000214
LOC100129550	1.30	2.45	5e-05	0.000214
FAM40B	1.29	2.45	5e-05	0.000214
AKR1C3	1.29	2.44	5e-05	0.000214
CALML4	1.28	2.43	5e-05	0.000214
LDLR	1.28	2.43	5e-05	0.000214
KBTBD8	1.28	2.42	5e-05	0.000214
PHF17	1.27	2.42	5e-05	0.000214
FAM110A	1.27	2.42	5e-05	0.000214
ZDHHC17	1.27	2.42	5e-05	0.000214
C1orf21	1.27	2.41	5e-05	0.000214
ITGA11	1.27	2.41	5e-05	0.000214
MARC2	1.27	2.41	5e-05	0.000214
DDX60L	1.27	2.41	5e-05	0.000214
MGAT4A	1.27	2.41	5e-05	0.000214
SNX29	1.27	2.41	5e-05	0.000214
NR4A1	1.26	2.40	5e-05	0.000214
CTSL1	1.26	2.40	5e-05	0.000214
TRIM16L	1.26	2.40	5e-05	0.000214
SH3BP2	1.26	2.39	5e-05	0.000214
KIAA1671	1.26	2.39	5e-05	0.000214
SPTBN1	1.25	2.39	5e-05	0.000214
PCDH9	1.25	2.38	5e-05	0.000214
DOK4	1.25	2.38	5e-05	0.000214
SLC29A1	1.25	2.38	5e-05	0.000214
NFAT5	1.25	2.37	5e-05	0.000214
ALDH3A2	1.25	2.37	5e-05	0.000214
IRF2BPL	1.25	2.37	5e-05	0.000214
LYN	1.24	2.37	5e-05	0.000214
HVCN1	1.24	2.37	0.014	0.033958

Supplementary Table 4 - continued

Gene name	Log2 fold change	Fold change	p-value	q-value
FRY	1.24	2.36	5e-05	0.000214
EPAS1	1.24	2.36	5e-05	0.000214
PIGZ	1.24	2.36	5e-05	0.000214
ZNF366	1.24	2.36	0.001	0.004564
CD59	1.24	2.36	5e-05	0.000214
PRSS3	1.23	2.35	5e-05	0.000214
PLEKHF1	1.23	2.34	5e-05	0.000214
HAPLN3	1.22	2.34	5e-05	0.000214
CSF1	1.22	2.33	5e-05	0.000214
GSN	1.22	2.33	5e-05	0.000214
NPEPPS	1.22	2.33	5e-05	0.000214
PROCR	1.21	2.32	5e-05	0.000214
SNX16	1.21	2.32	5e-05	0.000214
MMP24	1.21	2.31	5e-05	0.000214
TSPAN9	1.21	2.31	5e-05	0.000214
SLC2A13	1.20	2.30	5e-05	0.000214
NAAA	1.20	2.30	5e-05	0.000214
DNM3OS	1.20	2.30	0.001	0.003564
C19orf33	1.20	2.29	0.010	0.025519
PLTP	1.20	2.29	5e-05	0.000214
HEXIM2	1.19	2.29	0.011	0.027278
CD47	1.19	2.29	5e-05	0.000214
TBC1D2B	1.19	2.28	5e-05	0.000214
E2F7	1.19	2.28	5e-05	0.000214
LOC100132832	1.19	2.28	0.000	0.001281
ESYT2	1.19	2.28	5e-05	0.000214
ZFYVE28	1.19	2.28	5e-05	0.000214
ZCCHC24	1.19	2.28	5e-05	0.000214
TSPAN11	1.19	2.28	5e-05	0.000214
TNFAIP8L3	1.18	2.27	5e-05	0.000214
TGFBR3	1.18	2.27	5e-05	0.000214
FGF12	1.18	2.26	5e-05	0.000214
C5orf41	1.18	2.26	5e-05	0.000214
TMEM164	1.17	2.26	5e-05	0.000214
CLU	1.17	2.25	5e-05	0.000214
TMCC1	1.17	2.24	5e-05	0.000214
INSIG1	1.17	2.24	5e-05	0.000214
RHBDF1	1.16	2.24	5e-05	0.000214
SLC41A2	1.16	2.24	5e-05	0.000214
ELFN1	1.16	2.24	0.000	0.001281
GRN	1.16	2.24	5e-05	0.000214
OLFML3	1.16	2.24	0.002	0.006864
FUK	1.16	2.23	5e-05	0.000214
NRP2	1.16	2.23	5e-05	0.000214
ARMC7	1.16	2.23	5e-05	0.000214
BTN3A3	1.16	2.23	5e-05	0.000214
TYRO3	1.16	2.23	5e-05	0.000214
SLC37A1	1.16	2.23	5e-05	0.000214
NDP	1.16	2.23	0.005	0.012347
TMEM180	1.16	2.23	0.003	0.008907
TRIB2	1.15	2.22	5e-05	0.000214
EHBP1L1	1.15	2.22	5e-05	0.000214
ABCC1	1.15	2.22	5e-05	0.000214
MIR4315-1,PLEKHM1P	1.15	2.22	0.018	0.040589
C8orf4	1.15	2.22	5e-05	0.000214
PDLIM3	1.15	2.21	5e-05	0.000214
LRRC6	1.14	2.21	0.001	0.003278
PTPRG	1.14	2.21	5e-05	0.000214
CAMK2D	1.14	2.21	5e-05	0.000214
CAPN5	1.14	2.21	5e-05	0.000214
RAI2	1.14	2.20	5e-05	0.000214
CLDN14	1.14	2.20	0.004	0.011126
FCHO2	1.14	2.20	5e-05	0.000214

Supplementary Table 4 - continued

Gene name	Log2 fold change	Fold change	p-value	q-value
RORA	1.14	2.20	5e-05	0.000214
ZFHX3	1.14	2.20	5e-05	0.000214
BCL6B	1.13	2.20	5e-05	0.000214
TFF3	1.13	2.19	5e-05	0.000214
FAT1	1.13	2.19	5e-05	0.000214
FTL	1.13	2.19	5e-05	0.000214
FSTL1,MIR198	1.13	2.19	5e-05	0.000214
MIF4GD	1.13	2.18	0.013	0.030892
LMBRD1	1.13	2.18	5e-05	0.000214
TIMP2	1.13	2.18	5e-05	0.000214
CRMP1	1.12	2.18	5e-05	0.000214
TOB1	1.12	2.18	5e-05	0.000214
RGS11	1.12	2.18	0.019	0.042287
INPP4B	1.12	2.18	5e-05	0.000214
SPTAN1	1.12	2.17	5e-05	0.000214
GPR37	1.12	2.17	5e-05	0.000214
BMP1	1.11	2.16	5e-05	0.000214
BMPR1B	1.11	2.16	5e-05	0.000214
C22orf23	1.11	2.16	5e-05	0.000214
IFIT5	1.11	2.16	0.000	0.000946
FAM102A	1.11	2.16	5e-05	0.000214
HDAC5	1.11	2.16	5e-05	0.000214
TFEC	1.11	2.15	5e-05	0.000214
THSD1	1.10	2.15	5e-05	0.000214
CYB561D1	1.10	2.15	5e-05	0.000214
SYNC	1.10	2.14	5e-05	0.000214
AKD1	1.10	2.14	5e-05	0.000214
MAPK7	1.10	2.14	5e-05	0.000214
HTRA3	1.10	2.14	5e-05	0.000214
KLF3	1.09	2.13	5e-05	0.000214
S100A6	1.09	2.13	5e-05	0.000214
TMEM144	1.09	2.13	0.000	0.000595
FLJ44635	1.09	2.13	0.017	0.038838
LOC100132273	1.09	2.12	0.008	0.020483
ABCA7	1.09	2.12	5e-05	0.000214
FMNL3	1.08	2.12	5e-05	0.000214
PTPRM	1.08	2.12	5e-05	0.000214
MAPKAPK3	1.08	2.12	5e-05	0.000214
C15orf52	1.08	2.12	5e-05	0.000214
ZNF615	1.08	2.12	5e-05	0.000214
FOXO3	1.08	2.11	5e-05	0.000214
NID2	1.08	2.11	5e-05	0.000214
TENC1	1.08	2.11	5e-05	0.000214
SHANK3	1.08	2.11	5e-05	0.000214
CLIP4	1.08	2.11	5e-05	0.000214
FGF16	1.08	2.11	0.012	0.028819
KIAA1161	1.07	2.11	5e-05	0.000214
ANPEP	1.07	2.11	5e-05	0.000214
SMAGP	1.07	2.10	0.001	0.004413
LRRC8A	1.07	2.10	5e-05	0.000214
MTRNR2L8	1.07	2.10	5e-05	0.000214
BCL9L	1.07	2.10	5e-05	0.000214
OGDH	1.07	2.10	5e-05	0.000214
DHRS13	1.07	2.10	0.002	0.006455
ZNF555	1.07	2.09	5e-05	0.000214
INPP1	1.06	2.09	5e-05	0.000214
ERAP2	1.06	2.08	5e-05	0.000214
BMF	1.06	2.08	5e-05	0.000214
TANC2	1.06	2.08	5e-05	0.000214
DUSP3	1.05	2.08	5e-05	0.000214
CARHSP1	1.05	2.08	5e-05	0.000214
ARHGAP21	1.05	2.07	5e-05	0.000214
TMEM117	1.05	2.07	5e-05	0.000214

Supplementary Table 4 - continued

Gene name	Log2 fold change	Fold change	p-value	q-value
ADAMTS7	1.05	2.07	5e-05	0.000214
CD276	1.05	2.07	5e-05	0.000214
LOC100499466	1.05	2.07	5e-05	0.000214
RABGAP1L	1.05	2.07	5e-05	0.000214
IGIP	1.05	2.07	5e-05	0.000214
HFE	1.05	2.06	0.002	0.007135
AHNAK	1.04	2.06	5e-05	0.000214
IFIT3	1.04	2.06	0.001	0.003564
MOB2	1.04	2.06	0.008	0.020588
RRAS	1.04	2.06	5e-05	0.000214
SLC25A25	1.04	2.05	5e-05	0.000214
HM13	1.04	2.05	5e-05	0.000214
DNAH1	1.04	2.05	5e-05	0.000214
CNTNAP1	1.04	2.05	5e-05	0.000214
SGMS2	1.03	2.05	5e-05	0.000214
C1orf38	1.03	2.05	0.002	0.006316
HMCN1	1.03	2.04	0.011	0.026357
BCL2L1	1.03	2.04	5e-05	0.000214
KLF13	1.03	2.04	5e-05	0.000214
CPNE8	1.03	2.04	5e-05	0.000214
MMRN2	1.03	2.04	5e-05	0.000214
ZBTB47	1.03	2.04	5e-05	0.000214
PQLC3	1.03	2.04	5e-05	0.000214
ZNF527	1.03	2.04	0.001	0.002241
LRRC16A	1.03	2.04	5e-05	0.000214
HCN2	1.03	2.04	5e-05	0.000214
LAMA5,MIR4758	1.02	2.03	5e-05	0.000214
ID1	1.02	2.03	5e-05	0.000214
SLC31A2	1.02	2.03	5e-05	0.000214
TMEM45A	1.02	2.03	5e-05	0.000214
GLIS2	1.02	2.03	5e-05	0.000214
CASP10	1.02	2.03	5e-05	0.000214
TIPARP	1.02	2.02	5e-05	0.000214
ADRB2	1.02	2.02	0.001	0.003132
ST6GALNAC4	1.02	2.02	5e-05	0.000214
ZNRF2	1.01	2.02	0.000	0.000595
SSBP2	1.01	2.02	5e-05	0.000214
CD9	1.01	2.02	5e-05	0.000214
ARMCX1	1.01	2.02	5e-05	0.000214
IL6R	1.01	2.01	5e-05	0.000214
COL5A1	1.01	2.01	5e-05	0.000214
SSH2	1.00	2.01	5e-05	0.000214
CRISPLD1	1.00	2.00	5e-05	0.000214
TEF	1.00	2.00	5e-05	0.000214
RGS7BP	1.00	2.00	0.002	0.006591
LY96	1.00	2.00	5e-05	0.000214
NAGA	1.00	2.00	5e-05	0.000214
PLEKHG5,TNFRSF25	1.00	2.00	5e-05	0.000214
LOC201651	-1.00	0.50	0.004	0.011866
SOCS2	-1.00	0.50	0.003	0.008532
ZNF628	-1.00	0.50	5e-05	0.000214
HMGB1	-1.00	0.50	5e-05	0.000214
APLP1	-1.00	0.50	5e-05	0.000214
C12orf75	-1.01	0.50	5e-05	0.000214
GMDS	-1.01	0.50	5e-05	0.000214
EXOSC2	-1.01	0.50	5e-05	0.000214
YPEL2	-1.01	0.50	5e-05	0.000214
HHIP	-1.01	0.50	5e-05	0.000214
MTMR4	-1.01	0.50	5e-05	0.000214
TATDN1	-1.01	0.50	0.008	0.019528
FHL2	-1.01	0.50	5e-05	0.000214
PXMP2	-1.01	0.50	0.000	0.000946
GULP1	-1.01	0.50	5e-05	0.000214

Supplementary Table 4 - continued

Gene name	Log2 fold change	Fold change	p-value	q-value
C1orf226	-1.01	0.50	5e-04	0.001769
GAL3ST4	-1.01	0.50	0.005	0.013141
KLHL23,PHOSPHO2,PHOSPHO2-KLHL23	-1.01	0.50	5e-05	0.000214
NUP107	-1.01	0.49	5e-05	0.000214
PCNA	-1.02	0.49	5e-05	0.000214
EXOSC8	-1.02	0.49	4e-04	0.001444
APBA2	-1.02	0.49	5e-05	0.000214
NIT2	-1.02	0.49	5e-05	0.000214
PARD6G	-1.02	0.49	0.001	0.003419
EPB41L4A-AS1	-1.02	0.49	5e-05	0.000214
MYBBP1A	-1.02	0.49	2e-04	0.000770
TRABD	-1.02	0.49	5e-05	0.000214
CNN2	-1.02	0.49	5e-05	0.000214
METTL3	-1.02	0.49	2e-04	0.000770
WARS	-1.02	0.49	5e-05	0.000214
XIRP2	-1.02	0.49	0.007	0.018375
C1QBP	-1.02	0.49	5e-05	0.000214
VASH1	-1.02	0.49	5e-05	0.000214
MYO19	-1.03	0.49	5e-05	0.000214
BMP6	-1.03	0.49	0.020	0.045782
PAWR	-1.03	0.49	5e-05	0.000214
TPM1	-1.03	0.49	5e-05	0.000214
INHBA	-1.03	0.49	5e-05	0.000214
CD40	-1.03	0.49	5e-05	0.000214
LOC100506178	-1.03	0.49	1e-04	0.000410
TRAF4	-1.03	0.49	5e-05	0.000214
RUVBL2	-1.03	0.49	5e-05	0.000214
SNHG15	-1.03	0.49	5e-05	0.000214
TMCC3	-1.03	0.49	5e-05	0.000214
RGS10	-1.03	0.49	5e-05	0.000214
ZNF593	-1.03	0.49	5e-05	0.000214
MRTO4	-1.04	0.49	5e-05	0.000214
C4orf43	-1.04	0.49	5e-05	0.000214
WDR4	-1.04	0.49	5e-05	0.000214
NF2	-1.04	0.49	5e-05	0.000214
DSN1	-1.04	0.49	5e-05	0.000214
SRM	-1.04	0.49	5e-05	0.000214
SNTB1	-1.04	0.49	5e-05	0.000214
MYCN	-1.04	0.49	0.002	0.005650
CKAP2	-1.04	0.49	5e-05	0.000214
WRAP53	-1.04	0.49	0.015	0.034654
HMGA2	-1.04	0.48	5e-05	0.000214
SLC22A23	-1.04	0.48	5e-05	0.000214
KRT19	-1.05	0.48	5e-05	0.000214
ECI2	-1.05	0.48	5e-05	0.000214
PINX1	-1.05	0.48	5e-05	0.000214
EMG1	-1.05	0.48	5e-05	0.000214
RFX2	-1.05	0.48	5e-05	0.000214
GCN1L1	-1.05	0.48	5e-05	0.000214
FANK1	-1.05	0.48	0.006	0.016404
RFC5	-1.05	0.48	5e-05	0.000214
C14orf80	-1.05	0.48	5e-05	0.000214
HOMER1	-1.05	0.48	5e-05	0.000214
DOK3	-1.05	0.48	0.002	0.005918
NCAPD2	-1.06	0.48	5e-05	0.000214
ASGR1	-1.06	0.48	0.001	0.002841
TOX	-1.06	0.48	5e-05	0.000214
BPHL	-1.06	0.48	5e-05	0.000214
DUS3L	-1.06	0.48	5e-05	0.000214
GRAPL	-1.06	0.48	0.023	0.049939
CCDC85A	-1.06	0.48	5e-05	0.000214
MST4	-1.06	0.48	5e-05	0.000214
BNIP3	-1.07	0.48	5e-05	0.000214

Supplementary Table 4 - continued

Gene name	Log2 fold change	Fold change	p-value	q-value
NUP35	-1.07	0.48	5e-05	0.000214
BCKDHB	-1.07	0.48	4e-04	0.001444
MPP3	-1.07	0.48	0.018	0.040680
RBL1	-1.07	0.48	5e-05	0.000214
RFC2	-1.07	0.48	5e-05	0.000214
NLE1	-1.07	0.48	5e-05	0.000214
FBN2	-1.07	0.48	5e-05	0.000214
CENPL	-1.08	0.47	5e-05	0.000214
RELN	-1.08	0.47	5e-05	0.000214
LONRF3	-1.08	0.47	5e-05	0.000214
HYLS1	-1.08	0.47	0.003	0.008907
ATIC	-1.08	0.47	5e-05	0.000214
GMNN	-1.08	0.47	5e-05	0.000214
SPHK2	-1.08	0.47	5e-05	0.000214
SRSF3	-1.08	0.47	5e-05	0.000214
SRSF7	-1.08	0.47	5e-05	0.000214
MMP10	-1.08	0.47	5e-05	0.000214
EXOC6	-1.08	0.47	5e-05	0.000214
C20orf96	-1.08	0.47	5e-05	0.000214
HAUS8	-1.09	0.47	5e-05	0.000214
FIGN	-1.09	0.47	8e-04	0.002694
FBL	-1.09	0.47	5e-05	0.000214
NUDT4,NUDT4P1	-1.09	0.47	5e-05	0.000214
GOLGA8A	-1.09	0.47	5e-05	0.000214
BCL2L12	-1.09	0.47	0.004	0.010637
BCL2L11	-1.09	0.47	5e-05	0.000214
NAT14	-1.10	0.47	5e-05	0.000214
PAFAH1B3	-1.10	0.47	5e-05	0.000214
SASS6	-1.10	0.47	5e-04	0.001769
MRPL46	-1.10	0.47	5e-05	0.000214
FAIM	-1.10	0.47	0.003	0.007524
GEMIN2	-1.10	0.47	5e-05	0.000214
FAM60A	-1.10	0.47	5e-05	0.000214
RRP9	-1.10	0.47	5e-05	0.000214
NMT2	-1.11	0.46	5e-05	0.000214
DDT	-1.11	0.46	5e-05	0.000214
NOC3L	-1.11	0.46	5e-05	0.000214
GSDMD	-1.11	0.46	5e-05	0.000214
GNL3	-1.11	0.46	5e-05	0.000214
KIAA1274	-1.11	0.46	5e-05	0.000214
CABLES1	-1.11	0.46	5e-05	0.000214
LOC254896,TNFRSF10C	-1.11	0.46	5e-05	0.000214
ADK	-1.11	0.46	5e-05	0.000214
RCC1,SNHG3	-1.11	0.46	5e-05	0.000214
SAMD10	-1.11	0.46	0.001	0.002841
TTYH2	-1.11	0.46	0.001	0.003841
PRIM2	-1.11	0.46	5e-05	0.000214
ODC1	-1.11	0.46	5e-05	0.000214
NASP	-1.11	0.46	5e-05	0.000214
MURC	-1.11	0.46	0.001	0.001933
DKC1	-1.11	0.46	5e-05	0.000214
FAM203A	-1.12	0.46	0.005	0.013602
GLIPR1	-1.12	0.46	5e-05	0.000214
AXL	-1.12	0.46	5e-05	0.000214
SLC46A1	-1.12	0.46	0.000	0.000595
CTNNBIP1	-1.12	0.46	5e-05	0.000214
C16orf59	-1.12	0.46	0.001	0.002546
TBX1	-1.12	0.46	5e-05	0.000214
HNRNPD	-1.12	0.46	5e-05	0.000214
KLRG1	-1.12	0.46	0.004	0.012233
RRM1	-1.12	0.46	5e-05	0.000214
RGL1	-1.12	0.46	5e-05	0.000214
DUT	-1.13	0.46	5e-05	0.000214

Supplementary Table 4 - continued

Gene name	Log2 fold change	Fold change	p-value	q-value
PYCRL	-1.13	0.46	5e-05	0.000214
NARS2	-1.13	0.46	5e-05	0.000214
CHEK1	-1.13	0.46	5e-05	0.000214
UAP1	-1.13	0.46	5e-05	0.000214
ZNF704	-1.13	0.46	5e-05	0.000214
PPP1R35	-1.13	0.46	5e-05	0.000214
RHOBTB3	-1.13	0.46	5e-05	0.000214
SGCE	-1.13	0.46	5e-05	0.000214
SDC4	-1.13	0.46	5e-05	0.000214
RPS15	-1.13	0.46	5e-05	0.000214
FUS	-1.13	0.46	5e-05	0.000214
NCAPD3	-1.13	0.46	5e-05	0.000214
SLC7A5	-1.13	0.46	5e-05	0.000214
PSMC3IP	-1.13	0.46	1e-04	0.000410
FAM207A	-1.14	0.46	5e-05	0.000214
TUBB4B	-1.14	0.45	5e-05	0.000214
NPM1	-1.14	0.45	5e-05	0.000214
RANBP1	-1.14	0.45	5e-05	0.000214
ITGA4	-1.14	0.45	5e-05	0.000214
EFNA2	-1.14	0.45	0.001	0.003841
AGFG2	-1.14	0.45	5e-05	0.000214
SEMA6B	-1.14	0.45	5e-05	0.000214
SNRK	-1.14	0.45	5e-05	0.000214
DNAJC9	-1.14	0.45	5e-05	0.000214
RINL	-1.14	0.45	0.005	0.014550
SLC7A7	-1.14	0.45	5e-05	0.000214
MTHFD2L	-1.14	0.45	0.013	0.032080
TOE1	-1.14	0.45	5e-05	0.000214
LOC158376	-1.15	0.45	5e-05	0.000214
NINL	-1.15	0.45	5e-05	0.000214
POLA1	-1.15	0.45	5e-05	0.000214
GRAMD1C	-1.15	0.45	5e-05	0.000214
FAM124B	-1.15	0.45	5e-05	0.000214
GJA1	-1.15	0.45	5e-05	0.000214
CMC2	-1.15	0.45	1e-04	0.000410
HERC2P2	-1.15	0.45	5e-05	0.000214
PRMT1	-1.15	0.45	5e-05	0.000214
ZNF530	-1.15	0.45	0.008	0.021035
ITGB3BP	-1.16	0.45	5e-05	0.000214
RUNX1	-1.16	0.45	5e-05	0.000214
NLRC3	-1.16	0.45	5e-05	0.000214
RUVBL1	-1.16	0.45	5e-05	0.000214
SEMA6C	-1.16	0.45	5e-05	0.000214
MAGI1	-1.17	0.44	5e-05	0.000214
SIK1	-1.17	0.44	5e-05	0.000214
SLC39A4	-1.17	0.44	0.010	0.024184
KIAA0922	-1.17	0.44	5e-05	0.000214
TFRC	-1.18	0.44	5e-05	0.000214
RHPN1	-1.18	0.44	5e-05	0.000214
EFNA5	-1.18	0.44	5e-05	0.000214
MIR636,SRSF2	-1.18	0.44	5e-05	0.000214
MRPL3	-1.18	0.44	5e-05	0.000214
SEMA6D	-1.19	0.44	5e-05	0.000214
MPHOSPH6	-1.19	0.44	5e-05	0.000214
BRI3BP	-1.19	0.44	0.002	0.004706
LOC401588	-1.19	0.44	0.016	0.036986
ISOC1	-1.19	0.44	5e-05	0.000214
PCNA-AS1	-1.19	0.44	0.002	0.006997
SLC29A4	-1.19	0.44	5e-05	0.000214
SMO	-1.19	0.44	5e-05	0.000214
ELMOD1	-1.19	0.44	5e-05	0.000214
ZNF703	-1.19	0.44	5e-05	0.000214
ARID3A	-1.19	0.44	5e-05	0.000214

Supplementary Table 4 - continued

Gene name	Log2 fold change	Fold change	p-value	q-value
EFNB2	-1.20	0.44	5e-05	0.000214
AADAT	-1.20	0.44	0.002	0.006725
ST6GALNAC3	-1.20	0.43	5e-05	0.000214
PACSIN3	-1.20	0.43	5e-05	0.000214
AMOTL2	-1.20	0.43	5e-05	0.000214
TRAP1	-1.20	0.43	5e-05	0.000214
SNRNP25	-1.21	0.43	5e-05	0.000214
TMEM177	-1.21	0.43	5e-05	0.000214
NT5DC2	-1.21	0.43	5e-05	0.000214
ZNF714	-1.21	0.43	1e-04	0.000410
TCOF1	-1.21	0.43	5e-05	0.000214
LOC440288	-1.22	0.43	0.016	0.037080
SLC19A2	-1.22	0.43	5e-05	0.000214
PSTPIP2	-1.22	0.43	5e-05	0.000214
TMEM237	-1.22	0.43	5e-05	0.000214
SHF	-1.22	0.43	0.002	0.006051
ENOSF1	-1.22	0.43	5e-05	0.000214
C10orf2	-1.22	0.43	5e-05	0.000214
METTL17	-1.22	0.43	5e-05	0.000214
DYNLT1	-1.23	0.43	5e-05	0.000214
SUV39H1	-1.23	0.43	5e-05	0.000214
HOXD1	-1.23	0.43	0.002	0.006051
LOC388152	-1.23	0.43	5e-05	0.000214
POLD1	-1.23	0.43	5e-05	0.000214
CTPS	-1.23	0.43	5e-05	0.000214
SPOCD1	-1.23	0.43	5e-05	0.000214
PCSK1	-1.23	0.43	5e-05	0.000214
OBSL1	-1.23	0.43	5e-05	0.000214
RNF152	-1.23	0.43	5e-05	0.000214
POLE	-1.24	0.42	5e-05	0.000214
PIDD	-1.24	0.42	5e-05	0.000214
ZNF238	-1.24	0.42	5e-05	0.000214
TNFRSF11B	-1.24	0.42	0.009	0.021500
PKP4	-1.24	0.42	5e-05	0.000214
MIR3917,STMN1	-1.24	0.42	5e-05	0.000214
SLC17A9	-1.24	0.42	5e-05	0.000214
NOP58	-1.24	0.42	5e-05	0.000214
ERI1	-1.24	0.42	5e-05	0.000214
RNASEH2A	-1.24	0.42	5e-05	0.000214
MTHFD1	-1.25	0.42	5e-05	0.000214
MMP11	-1.25	0.42	5e-05	0.000214
HNRNPM	-1.25	0.42	5e-05	0.000214
TUBB	-1.25	0.42	5e-05	0.000214
NT5C	-1.25	0.42	0.016	0.037080
HNMT	-1.25	0.42	0.000	0.001605
DBF4B	-1.25	0.42	5e-05	0.000214
NRARP	-1.25	0.42	5e-05	0.000214
BCCIP	-1.25	0.42	5e-05	0.000214
ARID3B	-1.26	0.42	5e-05	0.000214
RPS6KA1	-1.26	0.42	5e-05	0.000214
C17orf96	-1.26	0.42	0.001	0.003564
TIPIN	-1.26	0.42	5e-05	0.000214
THOP1	-1.26	0.42	5e-05	0.000214
PTPN22	-1.26	0.42	0.003	0.008038
GGH	-1.26	0.42	5e-05	0.000214
LOXL1	-1.26	0.42	5e-05	0.000214
PPIF	-1.26	0.42	5e-05	0.000214
WDFY4	-1.26	0.42	5e-05	0.000214
CITED2	-1.26	0.42	5e-05	0.000214
C4orf21	-1.27	0.42	0.007	0.018605
SMS	-1.27	0.41	5e-05	0.000214
C4orf46	-1.27	0.41	5e-05	0.000214
EEF1E1,EEF1E1-MUTED,MUTED,MUTED-TXNDC5,TXNDC5	-1.27	0.41	5e-05	0.000214

Supplementary Table 4 - continued

Gene name	Log2 fold change	Fold change	p-value	q-value
C5orf55	-1.27	0.41	0.021	0.047500
GMFG	-1.27	0.41	5e-05	0.000214
MCM5	-1.28	0.41	5e-05	0.000214
MARCH4	-1.28	0.41	5e-05	0.000214
GPR116	-1.28	0.41	5e-05	0.000214
GPAT2	-1.28	0.41	0.007	0.018954
AIFM2	-1.28	0.41	5e-05	0.000214
KNTC1	-1.28	0.41	5e-05	0.000214
TUBA1C	-1.28	0.41	5e-05	0.000214
ANKRD1	-1.28	0.41	5e-05	0.000214
ZNF696	-1.28	0.41	1e-04	0.000410
CAP2	-1.29	0.41	1e-04	0.000410
SEMA3A	-1.29	0.41	5e-05	0.000214
RBMS3	-1.29	0.41	5e-05	0.000214
PCDHB5	-1.29	0.41	0.002	0.006455
GSG2	-1.29	0.41	0.015	0.035416
POLR3G	-1.29	0.41	5e-05	0.000214
POP1	-1.29	0.41	5e-05	0.000214
NUDT11	-1.30	0.41	5e-05	0.000214
H2AFZ	-1.30	0.41	5e-05	0.000214
CTTNBP2	-1.30	0.41	2e-04	0.000770
CCDC18	-1.30	0.41	0.000	0.000595
PODXL2	-1.30	0.41	5e-05	0.000214
ANKRD55	-1.30	0.41	0.017	0.039753
SKA2	-1.30	0.41	5e-05	0.000214
MMD	-1.30	0.41	5e-05	0.000214
UBASH3B	-1.30	0.40	5e-05	0.000214
RANBP17	-1.31	0.40	5e-05	0.000214
TTF2	-1.31	0.40	5e-05	0.000214
THSD7A	-1.31	0.40	5e-05	0.000214
DCXR	-1.31	0.40	5e-05	0.000214
PHF10	-1.32	0.40	5e-05	0.000214
CNN3	-1.32	0.40	5e-05	0.000214
PYCR1	-1.32	0.40	5e-05	0.000214
CD97	-1.32	0.40	5e-05	0.000214
BRCA1	-1.32	0.40	5e-05	0.000214
RHPN2	-1.32	0.40	0.000	0.000946
HNRNPA1,HNRNPA1P10	-1.33	0.40	5e-05	0.000214
BOP1	-1.33	0.40	5e-05	0.000214
GK	-1.33	0.40	5e-05	0.000214
EFNA1	-1.33	0.40	5e-05	0.000214
RAB3IP	-1.33	0.40	5e-05	0.000214
F2RL2	-1.33	0.40	5e-05	0.000214
ERV3-1,ZNF117	-1.34	0.40	5e-05	0.000214
STIL	-1.34	0.40	5e-05	0.000214
PHKA1	-1.34	0.40	5e-05	0.000214
CEP70	-1.34	0.39	0.000	0.000595
ID3	-1.35	0.39	5e-05	0.000214
CENPK	-1.35	0.39	5e-05	0.000214
CTXN1	-1.35	0.39	5e-05	0.000214
MGP	-1.35	0.39	5e-05	0.000214
EIF2B2	-1.35	0.39	5e-05	0.000214
ZNF579	-1.35	0.39	5e-05	0.000214
SEZ6L2	-1.35	0.39	5e-05	0.000214
VRK1	-1.35	0.39	5e-05	0.000214
DPYSL4	-1.35	0.39	5e-05	0.000214
EBNA1BP2	-1.35	0.39	5e-05	0.000214
C3orf26	-1.35	0.39	5e-05	0.000214
CERS1	-1.36	0.39	5e-05	0.000214
PFAS	-1.36	0.39	5e-05	0.000214
MYEOV	-1.36	0.39	0.016	0.036387
LOX	-1.36	0.39	5e-05	0.000214
FKBP11	-1.36	0.39	5e-05	0.000214

Supplementary Table 4 - continued

Gene name	Log2 fold change	Fold change	p-value	q-value
CHAF1A	-1.36	0.39	5e-05	0.000214
HMGB2	-1.37	0.39	5e-05	0.000214
ZNF22	-1.37	0.39	5e-05	0.000214
MYLK2	-1.37	0.39	0.001	0.003564
CACHD1	-1.37	0.39	5e-05	0.000214
CCHCR1	-1.37	0.39	0.005	0.012909
RELT	-1.37	0.39	5e-05	0.000214
SLC19A1	-1.37	0.39	5e-05	0.000214
FANCI	-1.37	0.39	5e-05	0.000214
ANKS3	-1.37	0.39	5e-05	0.000214
H2AFX	-1.37	0.39	5e-05	0.000214
HMGN2	-1.38	0.39	5e-05	0.000214
CDC7	-1.38	0.39	5e-05	0.000214
CDC42EP1	-1.38	0.38	5e-05	0.000214
WASF3	-1.38	0.38	5e-05	0.000214
GTF2H2C,GTF2H2D	-1.38	0.38	0.004	0.010139
MYCL1	-1.38	0.38	0.001	0.004275
PKD1L1	-1.38	0.38	5e-05	0.000214
EIF4EBP1	-1.39	0.38	5e-05	0.000214
SAT1	-1.39	0.38	5e-05	0.000214
SNHG10	-1.39	0.38	0.008	0.019957
GPR176	-1.39	0.38	5e-05	0.000214
FAM196B	-1.39	0.38	5e-05	0.000214
GCSH	-1.39	0.38	5e-05	0.000214
TXNRD2	-1.39	0.38	5e-05	0.000214
CAMK4	-1.39	0.38	0.001	0.003983
CCNE2	-1.40	0.38	0.002	0.004706
PHKA2	-1.40	0.38	5e-05	0.000214
ATAD3B	-1.40	0.38	5e-05	0.000214
LMNB2	-1.40	0.38	5e-05	0.000214
MEX3A	-1.40	0.38	5e-05	0.000214
SLC25A29	-1.40	0.38	5e-05	0.000214
TOP1MT	-1.40	0.38	5e-05	0.000214
TSPAN15	-1.40	0.38	5e-05	0.000214
FAM173A	-1.40	0.38	0.000	0.000946
ARSE	-1.40	0.38	4e-04	0.001444
FANCE	-1.40	0.38	5e-05	0.000214
LIMD2	-1.41	0.38	5e-05	0.000214
RNF19A	-1.41	0.38	5e-05	0.000214
LINC00338,SEC14L1	-1.41	0.38	5e-05	0.000214
CADM4	-1.41	0.38	5e-05	0.000214
ETV4	-1.41	0.38	5e-05	0.000214
FAM35A	-1.41	0.38	5e-05	0.000214
TIFA	-1.41	0.38	5e-05	0.000214
TNIK	-1.42	0.37	5e-05	0.000214
TUSC3	-1.42	0.37	5e-05	0.000214
MOCOS	-1.42	0.37	0.005	0.013602
CTNNAL1	-1.42	0.37	5e-05	0.000214
AKAP12	-1.43	0.37	5e-05	0.000214
FAM54A	-1.43	0.37	0.000	0.000595
TGFBI	-1.43	0.37	5e-05	0.000214
ALKBH6	-1.43	0.37	6e-04	0.002086
WDR27	-1.44	0.37	5e-05	0.000214
C9orf46	-1.44	0.37	5e-05	0.000214
FKBP5,LOC285847	-1.44	0.37	5e-05	0.000214
C19orf48	-1.44	0.37	5e-05	0.000214
RAB11FIP1	-1.44	0.37	5e-05	0.000214
ITM2C	-1.45	0.37	5e-05	0.000214
FIGNL1	-1.45	0.37	5e-05	0.000214
ECHDC2	-1.45	0.36	0.003	0.008796
STEAP1	-1.46	0.36	5e-05	0.000214
DANCR	-1.46	0.36	5e-05	0.000214
FSD1	-1.46	0.36	5e-05	0.000214

Supplementary Table 4 - continued

Gene name	Log2 fold change	Fold change	p-value	q-value
DHODH	-1.46	0.36	0.000	0.001605
DDAH1	-1.46	0.36	5e-05	0.000214
SPTBN2	-1.47	0.36	5e-05	0.000214
ARHGEF17	-1.47	0.36	5e-05	0.000214
KCNAB1	-1.47	0.36	5e-05	0.000214
CEND1	-1.47	0.36	0.011	0.026071
MYC	-1.47	0.36	5e-05	0.000214
TCF7	-1.48	0.36	0.001	0.003841
TMPO	-1.48	0.36	5e-05	0.000214
ARHGAP28	-1.48	0.36	5e-05	0.000214
FLJ41200	-1.48	0.36	5e-05	0.000214
PDE5A	-1.48	0.36	5e-05	0.000214
FAM84B	-1.48	0.36	5e-05	0.000214
BASP1	-1.49	0.36	5e-05	0.000214
CD3EAP	-1.49	0.36	0.004	0.010637
FSBP,RAD54B	-1.49	0.36	5e-05	0.000214
SLC25A10	-1.49	0.36	5e-05	0.000214
IL6	-1.49	0.36	0.012	0.029756
FANCA	-1.49	0.36	5e-05	0.000214
DTYMK	-1.49	0.36	5e-05	0.000214
DBF4	-1.49	0.36	5e-05	0.000214
CHST7	-1.50	0.35	5e-05	0.000214
SLC35F2	-1.50	0.35	5e-05	0.000214
SMYD2	-1.50	0.35	5e-05	0.000214
PM20D2	-1.51	0.35	5e-05	0.000214
SCARF2	-1.51	0.35	5e-05	0.000214
FBXO5	-1.51	0.35	5e-05	0.000214
GDF15	-1.51	0.35	5e-05	0.000214
MYO1B	-1.51	0.35	5e-05	0.000214
TMEM71	-1.52	0.35	0.001	0.003841
PRDM1	-1.52	0.35	5e-05	0.000214
CRIM1	-1.52	0.35	5e-05	0.000214
MCM7	-1.52	0.35	5e-05	0.000214
ARHGAP29	-1.52	0.35	5e-05	0.000214
TPM2	-1.52	0.35	5e-05	0.000214
C20orf27	-1.52	0.35	5e-05	0.000214
HOXA3	-1.52	0.35	5e-05	0.000214
B3GNTL1	-1.53	0.35	1e-04	0.000410
TRIM14	-1.53	0.35	0.005	0.014088
LOXL2	-1.53	0.35	5e-05	0.000214
POC1A	-1.53	0.35	5e-05	0.000214
AASS	-1.53	0.35	5e-05	0.000214
MCTP1	-1.54	0.35	5e-05	0.000214
HOTAIRM1	-1.54	0.34	2e-04	0.000770
MOV10L1	-1.54	0.34	5e-05	0.000214
WDR76	-1.54	0.34	5e-05	0.000214
MCM8	-1.54	0.34	5e-05	0.000214
HYI	-1.55	0.34	5e-05	0.000214
CCDC99	-1.55	0.34	5e-05	0.000214
SUSD5	-1.56	0.34	5e-05	0.000214
KIAA0040	-1.56	0.34	5e-05	0.000214
RPL39L	-1.56	0.34	5e-05	0.000214
EZH2	-1.56	0.34	5e-05	0.000214
SNRPA	-1.56	0.34	5e-05	0.000214
GPR3	-1.57	0.34	0.001	0.002546
MTBP	-1.57	0.34	5e-05	0.000214
TRO	-1.57	0.34	0.004	0.010022
PDE4B	-1.57	0.34	5e-05	0.000214
CCDC138	-1.57	0.34	4e-04	0.001444
MTHFD2	-1.57	0.34	5e-05	0.000214
MYH10	-1.57	0.34	5e-05	0.000214
TUBA1B	-1.57	0.34	5e-05	0.000214
CKS1B	-1.58	0.34	5e-05	0.000214

Supplementary Table 4 - continued

Gene name	Log2 fold change	Fold change	p-value	q-value
SNHG8	-1.58	0.33	5e-05	0.000214
RMI2	-1.58	0.33	1e-04	0.000410
PLOD2	-1.58	0.33	5e-05	0.000214
ULBP3	-1.58	0.33	0.007	0.017002
TGFB1I1	-1.59	0.33	5e-05	0.000214
RARG	-1.59	0.33	5e-05	0.000214
NRG1	-1.60	0.33	5e-05	0.000214
C1orf112	-1.60	0.33	3e-04	0.001114
MIS18A	-1.60	0.33	5e-05	0.000214
GOLGA8B	-1.60	0.33	5e-05	0.000214
OIP5	-1.60	0.33	0.002	0.005386
SLC45A4	-1.61	0.33	5e-05	0.000214
SPHK1	-1.62	0.32	5e-05	0.000214
CENPN	-1.63	0.32	5e-05	0.000214
ELMO3	-1.63	0.32	0.003	0.008166
CDH2	-1.63	0.32	5e-05	0.000214
MIF	-1.63	0.32	5e-05	0.000214
FAM216A	-1.63	0.32	0.000	0.000595
SLC9A7	-1.63	0.32	5e-05	0.000214
HBEGF	-1.64	0.32	5e-05	0.000214
LOC100129387	-1.64	0.32	0.022	0.049167
PAICS	-1.64	0.32	5e-05	0.000214
MCM2	-1.64	0.32	5e-05	0.000214
RALGPS2	-1.64	0.32	5e-05	0.000214
LOC729683	-1.65	0.32	2e-04	0.000770
NR2F1	-1.66	0.32	5e-05	0.000214
CECR6	-1.66	0.32	0.001	0.003564
CCDC34	-1.66	0.32	5e-05	0.000214
TCF7L1	-1.66	0.32	5e-05	0.000214
PDE1C	-1.67	0.32	0.000	0.001281
SNHG1	-1.67	0.31	5e-05	0.000214
LOC442421	-1.67	0.31	0.001	0.002241
WNK4	-1.67	0.31	5e-05	0.000214
ADM	-1.67	0.31	5e-05	0.000214
UBE2S	-1.68	0.31	5e-05	0.000214
BRCA2	-1.68	0.31	5e-05	0.000214
TMEM156	-1.69	0.31	5e-05	0.000214
ACN9	-1.70	0.31	0.000	0.000595
F2RL1	-1.70	0.31	5e-05	0.000214
CSGALNACT1	-1.71	0.30	5e-05	0.000214
ZNF711	-1.72	0.30	5e-05	0.000214
RGS4	-1.72	0.30	5e-05	0.000214
CREG2	-1.72	0.30	4e-04	0.001444
PSAT1	-1.72	0.30	5e-05	0.000214
ATAD5	-1.73	0.30	5e-05	0.000214
E2F1	-1.73	0.30	5e-05	0.000214
CD200	-1.73	0.30	5e-05	0.000214
FAM176A	-1.73	0.30	5e-05	0.000214
CDC25A	-1.73	0.30	5e-05	0.000214
STC2	-1.74	0.30	5e-05	0.000214
SUPT3H	-1.75	0.30	5e-05	0.000214
HELLS	-1.75	0.30	5e-05	0.000214
POU2F2	-1.75	0.30	5e-05	0.000214
PKDCC	-1.75	0.30	0.006	0.016879
MCM4	-1.76	0.30	5e-05	0.000214
SULT1C4	-1.76	0.29	5e-05	0.000214
FLT4	-1.76	0.29	5e-05	0.000214
XRCC6BP1	-1.77	0.29	0.006	0.015224
BLM	-1.77	0.29	5e-05	0.000214
ASRGL1	-1.78	0.29	5e-05	0.000214
LEPREL1	-1.78	0.29	5e-05	0.000214
TRAIP	-1.78	0.29	0.003	0.007524
DHFR	-1.78	0.29	0.006	0.016644

Supplementary Table 4 - continued

Gene name	Log2 fold change	Fold change	p-value	q-value
PRIM1	-1.79	0.29	5e-05	0.000214
PGF	-1.79	0.29	5e-05	0.000214
APEH	-1.80	0.29	5e-05	0.000214
FGFR4	-1.80	0.29	0.001	0.003132
NLGN1	-1.80	0.29	5e-05	0.000214
CENPJ	-1.80	0.29	5e-05	0.000214
GCA	-1.80	0.29	0.001	0.003132
BMP4	-1.80	0.29	5e-05	0.000214
EFEMP1	-1.80	0.29	5e-05	0.000214
ATOH8	-1.81	0.29	5e-05	0.000214
CDCA7L	-1.81	0.29	5e-05	0.000214
CNTNAP3	-1.81	0.29	5e-05	0.000214
C13orf15	-1.82	0.28	5e-05	0.000214
SHMT1	-1.82	0.28	0.006	0.014902
FJX1	-1.83	0.28	5e-05	0.000214
CETP	-1.84	0.28	0.003	0.007524
GTF2H2	-1.84	0.28	0.005	0.013836
EPHB6	-1.84	0.28	5e-05	0.000214
B4GALNT4	-1.85	0.28	5e-05	0.000214
CENPI	-1.85	0.28	5e-05	0.000214
CDKN2C	-1.85	0.28	5e-05	0.000214
FEN1	-1.86	0.28	5e-05	0.000214
IL1A	-1.87	0.27	0.000	0.001605
RASD1	-1.87	0.27	5e-05	0.000214
LMNB1	-1.87	0.27	5e-05	0.000214
PNMA2	-1.87	0.27	5e-05	0.000214
FBLL1	-1.87	0.27	0.018	0.040680
ARHGAP18	-1.88	0.27	5e-05	0.000214
LPPR1	-1.89	0.27	5e-05	0.000214
KRT18	-1.89	0.27	5e-05	0.000214
GINS4	-1.89	0.27	5e-05	0.000214
SGOL2	-1.89	0.27	5e-05	0.000214
CNTNAP3B	-1.89	0.27	5e-05	0.000214
PDLIM1	-1.89	0.27	5e-05	0.000214
TMEM37	-1.90	0.27	0.002	0.005386
FBXL16	-1.91	0.27	5e-05	0.000214
C21orf58	-1.91	0.27	2e-04	0.000770
C17orf53	-1.91	0.27	0.000	0.000595
CHST15	-1.92	0.26	5e-05	0.000214
RECQL4	-1.92	0.26	5e-05	0.000214
PARPBP	-1.94	0.26	5e-05	0.000214
SH2D5	-1.95	0.26	5e-05	0.000214
CLMN	-1.95	0.26	5e-05	0.000214
ARHGAP11A	-1.95	0.26	5e-05	0.000214
PREX2	-1.96	0.26	5e-05	0.000214
NCAPG2	-1.96	0.26	5e-05	0.000214
CYP2S1	-1.96	0.26	0.005	0.012909
IGFLR1	-1.97	0.26	0.022	0.048697
MYBL1	-1.97	0.26	5e-05	0.000214
WDR62	-1.97	0.26	5e-05	0.000214
MLF1IP	-1.97	0.25	5e-05	0.000214
CIT,MIR1178	-1.98	0.25	0.022	0.048789
SMC4	-1.98	0.25	5e-05	0.000214
GBA2	-1.99	0.25	0.002	0.005256
RFC3	-1.99	0.25	5e-05	0.000214
TCF19	-1.99	0.25	0.002	0.004986
MDK	-1.99	0.25	5e-05	0.000214
FAHD2B	-2.00	0.25	0.004	0.010139
CORO1A,LOC606724	-2.00	0.25	5e-05	0.000214
C15orf42	-2.00	0.25	5e-05	0.000214
GPRC5B	-2.01	0.25	5e-05	0.000214
HJURP	-2.02	0.25	5e-05	0.000214
NRXN3	-2.02	0.25	5e-05	0.000214

Supplementary Table 4 - continued

Gene name	Log2 fold change	Fold change	p-value	q-value
MFSD2A	-2.02	0.25	0.000	0.000595
MT1L	-2.03	0.25	0.003	0.008166
TMEM163	-2.03	0.25	5e-05	0.000214
PAK1	-2.03	0.24	5e-05	0.000214
TACC3	-2.03	0.24	5e-05	0.000214
CDC6	-2.03	0.24	5e-05	0.000214
DNA2	-2.03	0.24	5e-05	0.000214
SOX21	-2.04	0.24	0.004	0.010022
CXCL1	-2.04	0.24	0.000	0.000946
MELK	-2.04	0.24	5e-05	0.000214
PPP1R16B	-2.05	0.24	5e-05	0.000214
C4orf52	-2.05	0.24	5e-05	0.000214
CDC45	-2.05	0.24	5e-05	0.000214
TYMS	-2.05	0.24	5e-05	0.000214
MPP4	-2.05	0.24	5e-05	0.000214
CENPF	-2.05	0.24	5e-05	0.000214
HIC1	-2.06	0.24	5e-05	0.000214
ESM1	-2.06	0.24	5e-05	0.000214
FAM83D	-2.07	0.24	5e-05	0.000214
ZNF655	-2.07	0.24	5e-05	0.000214
RAD51	-2.07	0.24	5e-05	0.000214
HTR1B	-2.08	0.24	5e-05	0.000214
CTHRC1	-2.08	0.24	5e-05	0.000214
LOC643733	-2.08	0.24	5e-05	0.000214
ASNS	-2.09	0.24	5e-05	0.000214
MCM6	-2.09	0.24	5e-05	0.000214
TNR	-2.10	0.23	5e-05	0.000214
CPXM1	-2.11	0.23	5e-05	0.000214
CENPH	-2.12	0.23	5e-05	0.000214
BRIP1	-2.12	0.23	5e-05	0.000214
CDCA2	-2.12	0.23	5e-05	0.000214
CDCA5	-2.12	0.23	5e-05	0.000214
ATAD2	-2.13	0.23	5e-05	0.000214
CDT1	-2.13	0.23	5e-05	0.000214
PDGFB	-2.14	0.23	5e-05	0.000214
CBS	-2.14	0.23	5e-05	0.000214
UBE2T	-2.14	0.23	5e-05	0.000214
GCAT	-2.14	0.23	5e-05	0.000214
TAGLN	-2.15	0.23	0.002	0.004846
ALDOC	-2.15	0.23	4e-04	0.001444
HDDC2	-2.15	0.22	5e-05	0.000214
PDGFRL	-2.16	0.22	5e-05	0.000214
TROAP	-2.16	0.22	2e-04	0.000770
C15orf54	-2.16	0.22	5e-05	0.000214
KIF14	-2.17	0.22	5e-05	0.000214
AURKA	-2.17	0.22	5e-05	0.000214
CHTF18	-2.17	0.22	5e-05	0.000214
ESPL1	-2.18	0.22	5e-05	0.000214
GPX7	-2.19	0.22	0.002	0.005650
TSPAN13	-2.19	0.22	5e-05	0.000214
CEP72	-2.19	0.22	0.008	0.019856
KIF22	-2.19	0.22	5e-05	0.000214
EXO1	-2.20	0.22	5e-05	0.000214
CENPW	-2.21	0.22	5e-05	0.000214
KIF18A	-2.21	0.22	5e-05	0.000214
KIAA1524	-2.22	0.21	5e-05	0.000214
CTGF	-2.23	0.21	5e-05	0.000214
CEP128	-2.24	0.21	5e-05	0.000214
MILR1	-2.25	0.21	0.020	0.045326
NEIL3	-2.25	0.21	0.000	0.001281
ORC6	-2.25	0.21	5e-05	0.000214
SHISA3	-2.25	0.21	0.002	0.004986
TPPP3	-2.26	0.21	0.003	0.009776

Supplementary Table 4 - continued

Gene name	Log2 fold change	Fold change	p-value	q-value
POLE2	-2.26	0.21	5e-05	0.000214
MKI67	-2.26	0.21	5e-05	0.000214
CENPE	-2.28	0.21	5e-05	0.000214
MT2A	-2.28	0.21	5e-05	0.000214
CHST1	-2.28	0.21	5e-05	0.000214
NEXN	-2.29	0.20	5e-05	0.000214
RAD54L	-2.29	0.20	0.018	0.041679
SLC38A5	-2.30	0.20	0.001	0.003841
ESCO2	-2.30	0.20	5e-05	0.000214
KIF11	-2.30	0.20	5e-05	0.000214
SOX18	-2.30	0.20	5e-05	0.000214
TMEM97	-2.31	0.20	1e-04	0.000410
CSF3	-2.31	0.20	6e-04	0.002086
ZWINT	-2.32	0.20	5e-05	0.000214
DACH1	-2.32	0.20	5e-05	0.000214
PLK4	-2.32	0.20	5e-05	0.000214
FGD4	-2.32	0.20	5e-05	0.000214
C11orf82	-2.33	0.20	5e-05	0.000214
ORC1	-2.33	0.20	5e-05	0.000214
CENPV	-2.34	0.20	5e-05	0.000214
CDCA8	-2.35	0.20	5e-05	0.000214
ERCC6L	-2.36	0.20	0.000	0.000946
GRIK4	-2.37	0.19	0.002	0.006997
RPL22L1	-2.37	0.19	5e-05	0.000214
IQGAP3	-2.37	0.19	5e-05	0.000214
GPC2	-2.38	0.19	5e-05	0.000214
UHRF1	-2.39	0.19	5e-05	0.000214
CASC5	-2.40	0.19	5e-05	0.000214
LYPD6	-2.41	0.19	0.001	0.002546
SKA1	-2.41	0.19	5e-05	0.000214
TMEM88	-2.42	0.19	0.022	0.048049
ASPM	-2.43	0.19	5e-05	0.000214
POLQ	-2.43	0.18	5e-05	0.000214
NUSAP1	-2.44	0.18	5e-05	0.000214
TTK	-2.45	0.18	5e-05	0.000214
CYR61	-2.45	0.18	5e-05	0.000214
CEP55	-2.46	0.18	5e-05	0.000214
CCNA2	-2.46	0.18	5e-05	0.000214
GINS1	-2.46	0.18	5e-05	0.000214
TPX2	-2.46	0.18	5e-05	0.000214
CLDN7	-2.46	0.18	0.000	0.000595
KIF20B	-2.46	0.18	5e-05	0.000214
LOC100128881	-2.46	0.18	0.006	0.015688
HLX	-2.48	0.18	5e-05	0.000214
MT1X	-2.48	0.18	0.019	0.043306
ZNF367	-2.48	0.18	5e-05	0.000214
KCNF1	-2.48	0.18	0.014	0.033861
TOP2A	-2.48	0.18	5e-05	0.000214
DEPDC1B	-2.48	0.18	5e-05	0.000214
MAD2L1	-2.48	0.18	5e-05	0.000214
KIF23	-2.49	0.18	5e-05	0.000214
PRR11	-2.50	0.18	5e-05	0.000214
CDCA3	-2.51	0.18	5e-05	0.000214
CCNB1	-2.51	0.18	5e-05	0.000214
SOCS3	-2.54	0.17	5e-05	0.000214
PKMYT1	-2.54	0.17	5e-05	0.000214
CDK1	-2.54	0.17	5e-05	0.000214
CCNB2	-2.56	0.17	5e-05	0.000214
TEAD3	-2.56	0.17	0.002	0.005256
SFXN2	-2.56	0.17	0.018	0.042028
PIM3	-2.56	0.17	5e-05	0.000214
GMFB	-2.56	0.17	5e-05	0.000214
C9orf47,S1PR3	-2.57	0.17	5e-05	0.000214

Supplementary Table 4 - continued

Gene name	Log2 fold change	Fold change	p-value	q-value
RAD51AP1	-2.57	0.17	5e-05	0.000214
SPC25	-2.57	0.17	5e-05	0.000214
FOXM1	-2.58	0.17	5e-05	0.000214
FABP5	-2.59	0.17	5e-05	0.000214
DPF3	-2.59	0.17	5e-05	0.000214
ANLN	-2.60	0.16	5e-05	0.000214
DLL4	-2.61	0.16	5e-05	0.000214
ASF1B	-2.61	0.16	5e-05	0.000214
KIF4A	-2.62	0.16	5e-05	0.000214
CKAP2L	-2.62	0.16	5e-05	0.000214
CENPA	-2.62	0.16	5e-05	0.000214
LOC646324	-2.62	0.16	5e-05	0.000214
FBLN5	-2.63	0.16	0.003	0.008038
PRC1	-2.63	0.16	5e-05	0.000214
DIAPH3	-2.65	0.16	5e-05	0.000214
SHCBP1	-2.65	0.16	5e-05	0.000214
CLSPN	-2.66	0.16	5e-05	0.000214
DTL	-2.67	0.16	5e-05	0.000214
N4BP3	-2.67	0.16	5e-05	0.000214
RNF144A	-2.68	0.16	5e-05	0.000214
TMSB15A	-2.68	0.16	0.015	0.034654
SPAG5	-2.68	0.16	5e-05	0.000214
KIF18B	-2.69	0.16	5e-05	0.000214
NUF2	-2.69	0.16	5e-05	0.000214
GTSE1	-2.69	0.15	5e-05	0.000214
RACGAP1	-2.70	0.15	5e-05	0.000214
TK1	-2.70	0.15	5e-05	0.000214
CST1	-2.71	0.15	5e-05	0.000214
SPC24	-2.71	0.15	0.002	0.006051
UBE2C	-2.71	0.15	5e-05	0.000214
APOBEC3A,APOBEC3B	-2.72	0.15	6e-04	0.002086
KIF15	-2.74	0.15	5e-05	0.000214
CXADR	-2.75	0.15	5e-05	0.000214
KIF2C	-2.75	0.15	5e-05	0.000214
BIRC5	-2.76	0.15	5e-05	0.000214
PLK1	-2.77	0.15	5e-05	0.000214
CENPM	-2.77	0.15	5e-05	0.000214
SGOL1	-2.78	0.15	5e-05	0.000214
KIAA0101	-2.78	0.15	5e-05	0.000214
TRIP13	-2.79	0.14	5e-05	0.000214
PBK	-2.80	0.14	5e-05	0.000214
AMPH	-2.80	0.14	5e-05	0.000214
FAM111B	-2.81	0.14	5e-05	0.000214
FANCB	-2.83	0.14	0.003	0.008290
DSCC1	-2.84	0.14	0.001	0.003278
NEGR1	-2.84	0.14	5e-05	0.000214
THBS1	-2.85	0.14	5e-05	0.000214
XRCC2	-2.85	0.14	5e-05	0.000214
BDNF	-2.86	0.14	5e-05	0.000214
ACSS1	-2.86	0.14	5e-05	0.000214
NEK2	-2.86	0.14	5e-05	0.000214
ACE	-2.88	0.14	5e-05	0.000214
FANCD2	-2.88	0.14	5e-05	0.000214
AURKB	-2.89	0.13	5e-05	0.000214
NDC80	-2.91	0.13	5e-05	0.000214
NCAPH	-2.91	0.13	5e-05	0.000214
BUB1B,PAK6	-2.93	0.13	5e-05	0.000214
KRT80	-2.94	0.13	5e-05	0.000214
PIF1	-2.94	0.13	0.007	0.019160
GINS2	-2.94	0.13	5e-05	0.000214
NCAPG	-2.95	0.13	5e-05	0.000214
NR5A2	-2.95	0.13	5e-05	0.000214
SNCAIP	-2.95	0.13	5e-05	0.000214

Supplementary Table 4 - continued

Gene name	Log2 fold change	Fold change	p-value	q-value
ABCA4	-2.97	0.13	1e-04	0.000410
NOTCH3	-2.99	0.13	5e-05	0.000214
CXCR4	-3.00	0.13	5e-05	0.000214
PRSS12	-3.00	0.12	0.001	0.002241
SAPCD2	-3.03	0.12	5e-05	0.000214
DLGAP5	-3.03	0.12	5e-05	0.000214
LPPR3	-3.05	0.12	0.007	0.017576
DEPDC1	-3.09	0.12	5e-05	0.000214
BUB1	-3.10	0.12	5e-05	0.000214
RRM2	-3.10	0.12	5e-05	0.000214
KIF20A	-3.10	0.12	5e-05	0.000214
CDH6	-3.12	0.12	5e-05	0.000214
SKA3	-3.12	0.11	5e-05	0.000214
LXN	-3.13	0.11	5e-05	0.000214
MCM10	-3.14	0.11	5e-05	0.000214
ITGBL1	-3.15	0.11	0.009	0.022413
SLC7A2	-3.15	0.11	5e-05	0.000214
CDCA7	-3.17	0.11	5e-05	0.000214
MSMP	-3.19	0.11	5e-05	0.000214
PTTG1	-3.22	0.11	5e-05	0.000214
GDF6	-3.24	0.11	5e-05	0.000214
CCNA1	-3.25	0.10	5e-05	0.000214
MYBL2	-3.27	0.10	5e-05	0.000214
HMMR	-3.27	0.10	5e-05	0.000214
MAP2K6	-3.28	0.10	5e-05	0.000214
GNG2	-3.29	0.10	5e-05	0.000214
MGC72080	-3.32	0.10	0.004	0.011006
CDKN3	-3.35	0.10	0.000	0.000595
COL12A1	-3.36	0.10	5e-05	0.000214
CLDN11	-3.37	0.10	5e-05	0.000214
WDR69	-3.38	0.10	0.003	0.009271
LRRC17	-3.39	0.10	5e-05	0.000214
EDN1	-3.42	0.09	5e-05	0.000214
MT1E	-3.43	0.09	5e-05	0.000214
PEG10	-3.46	0.09	5e-05	0.000214
PDE7B	-3.47	0.09	5e-05	0.000214
RARB	-3.47	0.09	5e-05	0.000214
FAM64A	-3.49	0.09	5e-05	0.000214
RASSF2	-3.50	0.09	5e-05	0.000214
CDC20	-3.50	0.09	5e-05	0.000214
AIM1	-3.50	0.09	5e-05	0.000214
GJA4	-3.63	0.08	0.004	0.010515
C4orf49	-3.69	0.08	5e-05	0.000214
EFNB3	-3.72	0.08	0.001	0.003841
DKK1	-3.76	0.07	5e-05	0.000214
GPRIN3	-3.86	0.07	0.000	0.000595
CCL2	-3.86	0.07	5e-05	0.000214
LINC00176	-3.87	0.07	5e-05	0.000214
PHGDH	-3.88	0.07	5e-05	0.000214
RBM24	-3.98	0.06	5e-05	0.000214
HSD17B2	-3.98	0.06	0.003	0.007781
MEST	-4.30	0.05	5e-05	0.000214
IL8	-4.57	0.04	5e-05	0.000214
C10orf10	-4.65	0.04	5e-05	0.000214
CD24	-4.71	0.04	5e-05	0.000214
DHRS3	-4.79	0.04	5e-05	0.000214
FILIP1	-4.89	0.03	5e-05	0.000214
FABP4	-5.14	0.03	5e-05	0.000214
KRT7	-5.27	0.03	5e-05	0.000214
SEMA3G	-5.59	0.02	5e-05	0.000214
ANGPT2	-5.70	0.02	5e-05	0.000214
KIT	-5.80	0.02	5e-05	0.000214
CCL14,CCL14-CCL15,CCL15	-5.99	0.02	5e-05	0.000214

Supplementary Table 4 - continued

Gene name	Log2 fold change	Fold change	p-value	q-value
FAM172A,MIR2277	-8.35	0.003	5e-05	0.000214

Supplementary Table 5. Biological Process Gene Ontology terms that were significantly overrepresented in the analysis of the list of genes upregulated 2 times or more by the exposure to FSS.

Biological Process	GO number:	Over- (+) or underrepresented (-)	p-value
cellular process	GO:0009987	+	5.64E-12
Unclassified	-	-	2.42E-11
developmental process	GO:0032502	+	4.16E-10
cell adhesion	GO:0007155	+	8.86E-09
biological adhesion	GO:0022610	+	8.86E-09
cell communication	GO:0007154	+	2.56E-07
system development	GO:0048731	+	8.40E-07
cellular component movement	GO:0006928	+	3.76E-06
immune system process	GO:0002376	+	4.07E-06
mesoderm development	GO:0007498	+	3.29E-05
phosphate-containing compound metabolic process	GO:0006796	+	1.13E-04
anatomical structure morphogenesis	GO:0009653	+	2.97E-04
cell-cell adhesion	GO:0016337	+	4.57E-04
transport	GO:0006810	+	5.06E-04
localization	GO:0051179	+	1.68E-03
angiogenesis	GO:0001525	+	2.18E-03
response to stimulus	GO:0050896	+	4.81E-03
nervous system development	GO:0007399	+	6.04E-03
cellular component morphogenesis	GO:0032989	+	1.07E-02
mRNA processing	GO:0006397	-	2.03E-02
heart development	GO:0007507	+	4.80E-02

Supplementary Table 6. Biological Process Gene Ontology terms that were significantly overrepresented in the analysis of the list of genes downregulated 2 times or more by the exposure to FSS.

Biological Process	GO number:	Over- (+) or underrepresented (-)	p-value
cell cycle	GO:0007049	+	1.92E-16
cellular process	GO:0009987	+	1.28E-13
DNA metabolic process	GO:0006259	+	2.20E-13
DNA replication	GO:0006260	+	1.86E-11
chromosome segregation	GO:0007059	+	1.39E-09
Unclassified	-	-	2.03E-08
mitosis	GO:0007067	+	1.21E-07
metabolic process	GO:0008152	+	1.96E-04
cellular component organization	GO:0016043	+	9.02E-04
cytokinesis	GO:0000910	+	9.31E-04
cellular component organization or biogenesis	GO:0071840	+	1.12E-03
nucleobase-containing compound metabolic process	GO:0006139	+	3.13E-03
DNA repair	GO:0006281	+	4.13E-03
developmental process	GO:0032502	+	1.83E-02

Supplementary Table 7. The 103 genes that were upregulated 2 times or more by both EZH2-depletion and the exposure to FSS.

Associated Gene Name	Status (gene)	Description
ABLIM3	KNOWN	actin binding LIM protein family, member 3 [Source:HGNC Symbol;Acc:HGNC:29132]
ACP5	KNOWN	acid phosphatase 5, tartrate resistant [Source:HGNC Symbol;Acc:HGNC:124]
ADAM12	KNOWN	ADAM metallopeptidase domain 12 [Source:HGNC Symbol;Acc:HGNC:190]
ADPRH	KNOWN	ADP-ribosylarginine hydrolase [Source:HGNC Symbol;Acc:HGNC:269]
ADRB2	KNOWN	adrenoceptor beta 2, surface [Source:HGNC Symbol;Acc:HGNC:286]
ANO4	KNOWN	anoctamin 4 [Source:HGNC Symbol;Acc:HGNC:23837]
ANO4	KNOWN	anoctamin 4 [Source:HGNC Symbol;Acc:HGNC:23837]
APBA1	KNOWN	amyloid beta (A4) precursor protein-binding, family A, member 1 [Source:HGNC Symbol;Acc:HGNC:578]
APBA1	KNOWN	amyloid beta (A4) precursor protein-binding, family A, member 1 [Source:HGNC Symbol;Acc:HGNC:578]
ARHGAP6	KNOWN	Rho GTPase activating protein 6 [Source:HGNC Symbol;Acc:HGNC:676]
BAALC	KNOWN	brain and acute leukemia, cytoplasmic [Source:HGNC Symbol;Acc:HGNC:14333]
C10orf128	KNOWN	chromosome 10 open reading frame 128 [Source:HGNC Symbol;Acc:HGNC:27274]
C22orf23	KNOWN	chromosome 22 open reading frame 23 [Source:HGNC Symbol;Acc:HGNC:18589]
C8orf4	KNOWN	chromosome 8 open reading frame 4 [Source:HGNC Symbol;Acc:HGNC:1357]
CD47	KNOWN	CD47 molecule [Source:HGNC Symbol;Acc:HGNC:1682]
CDCP1	KNOWN	CUB domain containing protein 1 [Source:HGNC Symbol;Acc:HGNC:24357]
CFP	KNOWN	complement factor properdin [Source:HGNC Symbol;Acc:HGNC:8864]
CFP	KNOWN	complement factor properdin [Source:HGNC Symbol;Acc:HGNC:8864]
CLIC2	KNOWN	chloride intracellular channel 2 [Source:HGNC Symbol;Acc:HGNC:2063]
CLIP4	KNOWN	CAP-GLY domain containing linker protein family, member 4 [Source:HGNC Symbol;Acc:HGNC:26108]
CNTNAP1	KNOWN	contactin associated protein 1 [Source:HGNC Symbol;Acc:HGNC:8011]
CPE	KNOWN	carboxypeptidase E [Source:HGNC Symbol;Acc:HGNC:2303]
CSF1	KNOWN	colony stimulating factor 1 (macrophage) [Source:HGNC Symbol;Acc:HGNC:2432]
CSMD2	KNOWN	CUB and Sushi multiple domains 2 [Source:HGNC Symbol;Acc:HGNC:19290]
CYBRD1	KNOWN	cytochrome b reductase 1 [Source:HGNC Symbol;Acc:HGNC:20797]
DNAH1	KNOWN	dynein, axonemal, heavy chain 1 [Source:HGNC Symbol;Acc:HGNC:2940]
DNM3OS	KNOWN	DNM3 opposite strand/antisense RNA [Source:HGNC Symbol;Acc:HGNC:41228]
DUSP3	KNOWN	dual specificity phosphatase 3 [Source:HGNC Symbol;Acc:HGNC:3069]
ENKUR	KNOWN	enkurin, TRPC channel interacting protein [Source:HGNC Symbol;Acc:HGNC:28388]
FAM189A2	KNOWN	family with sequence similarity 189, member A2 [Source:HGNC Symbol;Acc:HGNC:24820]
FAS	KNOWN	Fas cell surface death receptor [Source:HGNC Symbol;Acc:HGNC:11920]
FAS	KNOWN	Fas cell surface death receptor [Source:HGNC Symbol;Acc:HGNC:11920]
FAT1	KNOWN	FAT atypical cadherin 1 [Source:HGNC Symbol;Acc:HGNC:3595]
FBN1	KNOWN	fibrillin 1 [Source:HGNC Symbol;Acc:HGNC:3603]
FLRT3	KNOWN	fibronectin leucine rich transmembrane protein 3 [Source:HGNC Symbol;Acc:HGNC:3762]
FNIP2	KNOWN	folliculin interacting protein 2 [Source:HGNC Symbol;Acc:HGNC:29280]
FTL	KNOWN	ferritin, light polypeptide [Source:HGNC Symbol;Acc:HGNC:3999]
FUCA1	KNOWN	fucosidase, alpha-L- 1, tissue [Source:HGNC Symbol;Acc:HGNC:4006]
GEM	KNOWN	GTP binding protein overexpressed in skeletal muscle [Source:HGNC Symbol;Acc:HGNC:4234]
GPR68	KNOWN	G protein-coupled receptor 68 [Source:HGNC Symbol;Acc:HGNC:4519]
GPX3	KNOWN	glutathione peroxidase 3 (plasma) [Source:HGNC Symbol;Acc:HGNC:4555]
GRN	KNOWN	granulin [Source:HGNC Symbol;Acc:HGNC:4601]
GRN	KNOWN	granulin [Source:HGNC Symbol;Acc:HGNC:4601]
GSN	KNOWN	gelsolin [Source:HGNC Symbol;Acc:HGNC:4620]
HFE	KNOWN	hemochromatosis [Source:HGNC Symbol;Acc:HGNC:4886]
HMCN1	KNOWN	hemicentin 1 [Source:HGNC Symbol;Acc:HGNC:19194]
HMOX1	KNOWN	heme oxygenase (decycling) 1 [Source:HGNC Symbol;Acc:HGNC:5013]
HPSE	KNOWN	heparanase [Source:HGNC Symbol;Acc:HGNC:5164]
ICAM1	KNOWN	intercellular adhesion molecule 1 [Source:HGNC Symbol;Acc:HGNC:5344]
IFI27	KNOWN	interferon, alpha-inducible protein 27 [Source:HGNC Symbol;Acc:HGNC:5397]
IGIP	KNOWN	IgA-inducing protein [Source:HGNC Symbol;Acc:HGNC:33847]
IL1R1	KNOWN	interleukin 1 receptor, type I [Source:HGNC Symbol;Acc:HGNC:5993]
ITGA3	KNOWN	integrin, alpha 3 (antigen CD49C, alpha 3 subunit of VLA-3 receptor) [Source:HGNC Symbol;Acc:HGNC:6139]
ITGA8	KNOWN	integrin, alpha 8 [Source:HGNC Symbol;Acc:HGNC:6144]
KBTBD8	KNOWN	kelch repeat and BTB (POZ) domain containing 8 [Source:HGNC Symbol;Acc:HGNC:30691]
KCNK3	KNOWN	potassium channel, subfamily K, member 3 [Source:HGNC Symbol;Acc:HGNC:6278]
KCNMB1	KNOWN	potassium large conductance calcium-activated channel, subfamily M, beta member 1 [Source:HGNC Symbol;Acc:HGNC:6285]
LINC00520	NOVEL	long intergenic non-protein coding RNA 520 [Source:HGNC Symbol;Acc:HGNC:19843]
LRRC16A	KNOWN	leucine rich repeat containing 16A [Source:HGNC Symbol;Acc:HGNC:21581]
LYST	KNOWN	lysosomal trafficking regulator [Source:HGNC Symbol;Acc:HGNC:1968]
MAPK13	KNOWN	mitogen-activated protein kinase 13 [Source:HGNC Symbol;Acc:HGNC:6875]
MLF2	KNOWN	myeloid leukemia factor 2 [Source:HGNC Symbol;Acc:HGNC:7126]
MATN2	KNOWN	matrilin 2 [Source:HGNC Symbol;Acc:HGNC:6908]
MFI2	KNOWN	antigen p97 (melanoma associated) identified by monoclonal antibodies 133.2 and 96.5 [Source:HGNC Symbol;Acc:HGNC:7037]
MIR31HG	NOVEL	MIR31 host gene (non-protein coding) [Source:HGNC Symbol;Acc:HGNC:37187]
NAAA	KNOWN	N-acylethanolamine acid amidase [Source:HGNC Symbol;Acc:HGNC:736]
NID2	KNOWN	nidogen 2 (osteonidogen) [Source:HGNC Symbol;Acc:HGNC:13389]
NT5E	KNOWN	5'-nucleotidase, ecto (CD73) [Source:HGNC Symbol;Acc:HGNC:8021]
OLFML3	KNOWN	olfactomedin-like 3 [Source:HGNC Symbol;Acc:HGNC:24956]
OSGIN1	KNOWN	oxidative stress induced growth inhibitor 1 [Source:HGNC Symbol;Acc:HGNC:30093]
PAPPA	KNOWN	pregnancy-associated plasma protein A, pappalysin 1 [Source:HGNC Symbol;Acc:HGNC:8602]
PARP15	KNOWN	poly (ADP-ribose) polymerase family, member 15 [Source:HGNC Symbol;Acc:HGNC:26876]

Supplementary Table 7 - continued

Associated Gene Name	Status (gene)	Description
PFN1P2	KNOWN	profilin 1 pseudogene 2 [Source:HGNC Symbol;Acc:HGNC:24298]
PIGZ	KNOWN	phosphatidylinositol glycan anchor biosynthesis, class Z [Source:HGNC Symbol;Acc:HGNC:30596]
PPP1R3C	KNOWN	protein phosphatase 1, regulatory subunit 3C [Source:HGNC Symbol;Acc:HGNC:9293]
PTGS1	KNOWN	prostaglandin-endoperoxide synthase 1 (prostaglandin G/H synthase and cyclooxygenase) [Source:HGNC Symbol;Acc:HGNC:9604]
PTK2B	KNOWN	protein tyrosine kinase 2 beta [Source:HGNC Symbol;Acc:HGNC:9612]
QPCT	KNOWN	glutaminyl-peptide cyclotransferase [Source:HGNC Symbol;Acc:HGNC:9753]
RAI2	KNOWN	retinoic acid induced 2 [Source:HGNC Symbol;Acc:HGNC:9835]
RASA4	KNOWN	RAS p21 protein activator 4 [Source:HGNC Symbol;Acc:HGNC:23181]
RENBP	KNOWN	renin binding protein [Source:HGNC Symbol;Acc:HGNC:9959]
RGS11	KNOWN	regulator of G-protein signaling 11 [Source:HGNC Symbol;Acc:HGNC:9993]
RRAS	KNOWN	related RAS viral (r-ras) oncogene homolog [Source:HGNC Symbol;Acc:HGNC:10447]
SELP	KNOWN	selectin P (granule membrane protein 140kDa, antigen CD62) [Source:HGNC Symbol;Acc:HGNC:10721]
TRMT1	KNOWN	tRNA methyltransferase 1 homolog (S. cerevisiae) [Source:HGNC Symbol;Acc:HGNC:25980]
SEMA4B	KNOWN	*sema domain, immunoglobulin domain (Ig), transmembrane domain (TM) and short cytoplasmic domain, (semaphorin) 4B [Source:HGNC Sym
SERPINB2	KNOWN	serpin peptidase inhibitor, clade B (ovalbumin), member 2 [Source:HGNC Symbol;Acc:HGNC:8584]
SERPINI1	KNOWN	serpin peptidase inhibitor, clade I (neuroserpin), member 1 [Source:HGNC Symbol;Acc:HGNC:8943]
SLC16A4	KNOWN	solute carrier family 16, member 4 [Source:HGNC Symbol;Acc:HGNC:10925]
SLC17A5	KNOWN	solute carrier family 17 (acidic sugar transporter), member 5 [Source:HGNC Symbol;Acc:HGNC:10933]
SLC2A13	KNOWN	solute carrier family 2 (facilitated glucose transporter), member 13 [Source:HGNC Symbol;Acc:HGNC:15956]
SLC41A2	KNOWN	solute carrier family 41 (magnesium transporter), member 2 [Source:HGNC Symbol;Acc:HGNC:31045]
SPTBN5	KNOWN	spectrin, beta, non-erythrocytic 5 [Source:HGNC Symbol;Acc:HGNC:15680]
ST8SIA6	KNOWN	ST8 alpha-N-acetyl-neuraminide alpha-2,8-sialyltransferase 6 [Source:HGNC Symbol;Acc:HGNC:23317]
STC1	KNOWN	stanniocalcin 1 [Source:HGNC Symbol;Acc:HGNC:11373]
SYNE1	KNOWN	spectrin repeat containing, nuclear envelope 1 [Source:HGNC Symbol;Acc:HGNC:17089]
TEK	KNOWN	TEK tyrosine kinase, endothelial [Source:HGNC Symbol;Acc:HGNC:11724]
TFEC	KNOWN	transcription factor EC [Source:HGNC Symbol;Acc:HGNC:11754]
TGFBR3	KNOWN	transforming growth factor, beta receptor III [Source:HGNC Symbol;Acc:HGNC:11774]
TIMP2	KNOWN	TIMP metallopeptidase inhibitor 2 [Source:HGNC Symbol;Acc:HGNC:11821]
TMEM38A	KNOWN	transmembrane protein 38A [Source:HGNC Symbol;Acc:HGNC:28462]
TNFRSF11A	KNOWN	tumor necrosis factor receptor superfamily, member 11a, NFKB activator [Source:HGNC Symbol;Acc:HGNC:11908]
TNFRSF11A	KNOWN	tumor necrosis factor receptor superfamily, member 11a, NFKB activator [Source:HGNC Symbol;Acc:HGNC:11908]
TRNP1	KNOWN	TMF1-regulated nuclear protein 1 [Source:HGNC Symbol;Acc:HGNC:34348]
TSPAN11	KNOWN	tetraspanin 11 [Source:HGNC Symbol;Acc:HGNC:30795]
TUBA4A	KNOWN	tubulin, alpha 4a [Source:HGNC Symbol;Acc:HGNC:12407]
TXNIP	KNOWN	thioredoxin interacting protein [Source:HGNC Symbol;Acc:HGNC:16952]
VCAN	KNOWN	versican [Source:HGNC Symbol;Acc:HGNC:2464]
WDR66	KNOWN	WD repeat domain 66 [Source:HGNC Symbol;Acc:HGNC:28506]

*sema domain, immunoglobulin domain (Ig), transmembrane domain (TM) and short cytoplasmic domain, (semaphorin) 4B [Source:HGNC Symbol;Acc:HGNC:10730]

Supplementary Table 8. The 355 genes that were downregulated 2 times or more by both EZH2-depletion and the exposure to FSS.

Associated Gene Name	Status (gene)	Description
ABCA4	KNOWN	ATP-binding cassette, sub-family A (ABC1), member 4 [Source:HGNC Symbol;Acc:HGNC:34]
ACE	KNOWN	angiotensin I converting enzyme [Source:HGNC Symbol;Acc:HGNC:2707]
ACSS1	KNOWN	acyl-CoA synthetase short-chain family member 1 [Source:HGNC Symbol;Acc:HGNC:16091]
AMPH	KNOWN	amphiphysin [Source:HGNC Symbol;Acc:HGNC:471]
ANLN	KNOWN	anillin, actin binding protein [Source:HGNC Symbol;Acc:HGNC:14082]
ARHGAP11A	KNOWN	Rho GTPase activating protein 11A [Source:HGNC Symbol;Acc:HGNC:15783]
ARHGAP18	KNOWN	Rho GTPase activating protein 18 [Source:HGNC Symbol;Acc:HGNC:21035]
ARSE	KNOWN	arylsulfatase E (chondrodysplasia punctata 1) [Source:HGNC Symbol;Acc:HGNC:719]
ASF1B	KNOWN	anti-silencing function 1B histone chaperone [Source:HGNC Symbol;Acc:HGNC:20996]
ASGR1	KNOWN	asialoglycoprotein receptor 1 [Source:HGNC Symbol;Acc:HGNC:742]
ASPM	KNOWN	asp (abnormal spindle) homolog, microcephaly associated (Drosophila) [Source:HGNC Symbol;Acc:HGNC:19048]
ATAD2	KNOWN	ATPase family, AAA domain containing 2 [Source:HGNC Symbol;Acc:HGNC:30123]
ATAD5	KNOWN	ATPase family, AAA domain containing 5 [Source:HGNC Symbol;Acc:HGNC:25752]
AURKA	KNOWN	aurora kinase A [Source:HGNC Symbol;Acc:HGNC:11393]
AURKB	KNOWN	aurora kinase B [Source:HGNC Symbol;Acc:HGNC:11390]
B4GALNT4	KNOWN	beta-1,4-N-acetyl-galactosaminyl transferase 4 [Source:HGNC Symbol;Acc:HGNC:26315]
BCL2L11	KNOWN	BCL2-like 11 (apoptosis facilitator) [Source:HGNC Symbol;Acc:HGNC:994]
BIRC5	KNOWN	baculoviral IAP repeat containing 5 [Source:HGNC Symbol;Acc:HGNC:593]
BLM	KNOWN	Bloom syndrome, RecQ helicase-like [Source:HGNC Symbol;Acc:HGNC:1058]
BLM	KNOWN	Bloom syndrome, RecQ helicase-like [Source:HGNC Symbol;Acc:HGNC:1058]
BMP4	KNOWN	bone morphogenetic protein 4 [Source:HGNC Symbol;Acc:HGNC:1071]
BRCA1	KNOWN	breast cancer 1, early onset [Source:HGNC Symbol;Acc:HGNC:1100]
BRCA1	KNOWN	breast cancer 1, early onset [Source:HGNC Symbol;Acc:HGNC:1100]
BRCA2	KNOWN	breast cancer 2, early onset [Source:HGNC Symbol;Acc:HGNC:1101]
BRCA2	KNOWN	breast cancer 2, early onset [Source:HGNC Symbol;Acc:HGNC:1101]
BRI3BP	KNOWN	BRI3 binding protein [Source:HGNC Symbol;Acc:HGNC:14251]
BRIP1	KNOWN	BRCA1 interacting protein C-terminal helicase 1 [Source:HGNC Symbol;Acc:HGNC:20473]
BRIP1	KNOWN	BRCA1 interacting protein C-terminal helicase 1 [Source:HGNC Symbol;Acc:HGNC:20473]
BUB1	KNOWN	BUB1 mitotic checkpoint serine/threonine kinase [Source:HGNC Symbol;Acc:HGNC:1148]
BUB1B	KNOWN	BUB1 mitotic checkpoint serine/threonine kinase B [Source:HGNC Symbol;Acc:HGNC:1149]
BUB1B	KNOWN	BUB1 mitotic checkpoint serine/threonine kinase B [Source:HGNC Symbol;Acc:HGNC:1149]
C10orf10	KNOWN	chromosome 10 open reading frame 10 [Source:HGNC Symbol;Acc:HGNC:23355]
C14orf80	KNOWN	chromosome 14 open reading frame 80 [Source:HGNC Symbol;Acc:HGNC:20127]
C16orf59	KNOWN	chromosome 16 open reading frame 59 [Source:HGNC Symbol;Acc:HGNC:25849]
C17orf53	KNOWN	chromosome 17 open reading frame 53 [Source:HGNC Symbol;Acc:HGNC:28460]
C1orf112	KNOWN	chromosome 1 open reading frame 112 [Source:HGNC Symbol;Acc:HGNC:25565]
C21orf58	KNOWN	chromosome 21 open reading frame 58 [Source:HGNC Symbol;Acc:HGNC:1300]
C4orf46	KNOWN	chromosome 4 open reading frame 46 [Source:HGNC Symbol;Acc:HGNC:27320]
CAMK4	KNOWN	calcium/calmodulin-dependent protein kinase IV [Source:HGNC Symbol;Acc:HGNC:1464]
CASC5	KNOWN	cancer susceptibility candidate 5 [Source:HGNC Symbol;Acc:HGNC:24054]
CCDC138	KNOWN	coiled-coil domain containing 138 [Source:HGNC Symbol;Acc:HGNC:26531]
CCDC34	KNOWN	coiled-coil domain containing 34 [Source:HGNC Symbol;Acc:HGNC:25079]
CCL14	KNOWN	chemokine (C-C motif) ligand 14 [Source:HGNC Symbol;Acc:HGNC:10612]
CCL15	KNOWN	chemokine (C-C motif) ligand 15 [Source:HGNC Symbol;Acc:HGNC:10613]
CCNA1	KNOWN	cyclin A1 [Source:HGNC Symbol;Acc:HGNC:1577]
CCNA2	KNOWN	cyclin A2 [Source:HGNC Symbol;Acc:HGNC:1578]
CCNB1	KNOWN	cyclin B1 [Source:HGNC Symbol;Acc:HGNC:1579]
CCNB2	KNOWN	cyclin B2 [Source:HGNC Symbol;Acc:HGNC:1580]
CCNE2	KNOWN	cyclin E2 [Source:HGNC Symbol;Acc:HGNC:1590]
CD24	KNOWN	CD24 molecule [Source:HGNC Symbol;Acc:HGNC:1645]
CDC20	KNOWN	cell division cycle 20 [Source:HGNC Symbol;Acc:HGNC:1723]
CDC25A	KNOWN	cell division cycle 25A [Source:HGNC Symbol;Acc:HGNC:1725]
CDC45	KNOWN	cell division cycle 45 [Source:HGNC Symbol;Acc:HGNC:1739]
CDC6	KNOWN	cell division cycle 6 [Source:HGNC Symbol;Acc:HGNC:1744]
CDC7	KNOWN	cell division cycle 7 [Source:HGNC Symbol;Acc:HGNC:1745]
CDCA2	KNOWN	cell division cycle associated 2 [Source:HGNC Symbol;Acc:HGNC:14623]
CDCA3	KNOWN	cell division cycle associated 3 [Source:HGNC Symbol;Acc:HGNC:14624]
CDCA5	KNOWN	cell division cycle associated 5 [Source:HGNC Symbol;Acc:HGNC:14626]
CDCA7	KNOWN	cell division cycle associated 7 [Source:HGNC Symbol;Acc:HGNC:14628]
CDCA8	KNOWN	cell division cycle associated 8 [Source:HGNC Symbol;Acc:HGNC:14629]
CDK1	KNOWN	cyclin-dependent kinase 1 [Source:HGNC Symbol;Acc:HGNC:1722]
CDKN2C	KNOWN	cyclin-dependent kinase inhibitor 2C (p18, inhibits CDK4) [Source:HGNC Symbol;Acc:HGNC:1789]
CDKN3	KNOWN	cyclin-dependent kinase inhibitor 3 [Source:HGNC Symbol;Acc:HGNC:1791]
CDT1	KNOWN	chromatin licensing and DNA replication factor 1 [Source:HGNC Symbol;Acc:HGNC:24576]
CENPA	KNOWN	centromere protein A [Source:HGNC Symbol;Acc:HGNC:1851]
CENPE	KNOWN	centromere protein E, 312kDa [Source:HGNC Symbol;Acc:HGNC:1856]
CENPF	KNOWN	centromere protein F, 350/400kDa [Source:HGNC Symbol;Acc:HGNC:1857]
CENPH	KNOWN	centromere protein H [Source:HGNC Symbol;Acc:HGNC:17268]
CENPI	KNOWN	centromere protein I [Source:HGNC Symbol;Acc:HGNC:3968]
CENPJ	KNOWN	centromere protein J [Source:HGNC Symbol;Acc:HGNC:17272]
CENPK	KNOWN	centromere protein K [Source:HGNC Symbol;Acc:HGNC:29479]
CENPL	KNOWN	centromere protein L [Source:HGNC Symbol;Acc:HGNC:17879]
CENPM	KNOWN	centromere protein M [Source:HGNC Symbol;Acc:HGNC:18352]
CENPV	KNOWN	centromere protein V [Source:HGNC Symbol;Acc:HGNC:29920]
CENPW	KNOWN	centromere protein W [Source:HGNC Symbol;Acc:HGNC:21488]

Supplementary Table 8 - continued

Associated Gene Name	Status (gene)	Description
CEP128	KNOWN	centrosomal protein 128kDa [Source:HGNC Symbol;Acc:HGNC:20359]
CEP55	KNOWN	centrosomal protein 55kDa [Source:HGNC Symbol;Acc:HGNC:1161]
CEP72	KNOWN	centrosomal protein 72kDa [Source:HGNC Symbol;Acc:HGNC:25547]
CHAF1A	KNOWN	chromatin assembly factor 1, subunit A (p150) [Source:HGNC Symbol;Acc:HGNC:1910]
CHEK1	KNOWN	checkpoint kinase 1 [Source:HGNC Symbol;Acc:HGNC:1925]
CHST1	KNOWN	carbohydrate (keratan sulfate Gal-6) sulfotransferase 1 [Source:HGNC Symbol;Acc:HGNC:1969]
CHST15	KNOWN	carbohydrate (N-acetylgalactosamine 4-sulfate 6-O) sulfotransferase 15 [Source:HGNC Symbol;Acc:HGNC:18137]
CHTF18	KNOWN	CTF18, chromosome transmission fidelity factor 18 homolog (S. cerevisiae) [Source:HGNC Symbol;Acc:HGNC:18435]
CIT	KNOWN	citron rho-interacting serine/threonine kinase [Source:HGNC Symbol;Acc:HGNC:1985]
CKAP2	KNOWN	cytoskeleton associated protein 2 [Source:HGNC Symbol;Acc:HGNC:1990]
CKAP2L	KNOWN	cytoskeleton associated protein 2-like [Source:HGNC Symbol;Acc:HGNC:26877]
CKS1B	KNOWN	CDC28 protein kinase regulatory subunit 1B [Source:HGNC Symbol;Acc:HGNC:19083]
CLDN7	KNOWN	claudin 7 [Source:HGNC Symbol;Acc:HGNC:2049]
CLSPN	KNOWN	claspin [Source:HGNC Symbol;Acc:HGNC:19715]
COL12A1	KNOWN	collagen, type XII, alpha 1 [Source:HGNC Symbol;Acc:HGNC:2188]
CORO1A	KNOWN	coronin, actin binding protein, 1A [Source:HGNC Symbol;Acc:HGNC:2252]
CORO1A	KNOWN	coronin, actin binding protein, 1A [Source:HGNC Symbol;Acc:HGNC:2252]
CREG2	KNOWN	cellular repressor of E1A-stimulated genes 2 [Source:HGNC Symbol;Acc:HGNC:14272]
CTNNAL1	KNOWN	catenin (cadherin-associated protein), alpha-like 1 [Source:HGNC Symbol;Acc:HGNC:2512]
CXADR	KNOWN	coxsackie virus and adenovirus receptor [Source:HGNC Symbol;Acc:HGNC:2559]
DBF4	KNOWN	DBF4 zinc finger [Source:HGNC Symbol;Acc:HGNC:17364]
DBF4B	KNOWN	DBF4 zinc finger B [Source:HGNC Symbol;Acc:HGNC:17883]
DCXR	KNOWN	dicarbonyl/L-xylulose reductase [Source:HGNC Symbol;Acc:HGNC:18985]
DEPDC1	KNOWN	DEP domain containing 1 [Source:HGNC Symbol;Acc:HGNC:22949]
DEPDC1B	KNOWN	DEP domain containing 1B [Source:HGNC Symbol;Acc:HGNC:24902]
DHFR	KNOWN	dihydrofolate reductase [Source:HGNC Symbol;Acc:HGNC:2861]
DHRS3	KNOWN	dehydrogenase/reductase (SDR family) member 3 [Source:HGNC Symbol;Acc:HGNC:17693]
DIAPH3	KNOWN	diaphanous-related formin 3 [Source:HGNC Symbol;Acc:HGNC:15480]
DKK1	KNOWN	dickkopf WNT signaling pathway inhibitor 1 [Source:HGNC Symbol;Acc:HGNC:2891]
DLGAP5	KNOWN	discs, large (Drosophila) homolog-associated protein 5 [Source:HGNC Symbol;Acc:HGNC:16864]
DLL4	KNOWN	delta-like 4 (Drosophila) [Source:HGNC Symbol;Acc:HGNC:2910]
DNA2	KNOWN	DNA replication helicase/nuclease 2 [Source:HGNC Symbol;Acc:HGNC:2939]
DSCC1	KNOWN	DNA replication and sister chromatid cohesion 1 [Source:HGNC Symbol;Acc:HGNC:24453]
DTL	KNOWN	denticleless E3 ubiquitin protein ligase homolog (Drosophila) [Source:HGNC Symbol;Acc:HGNC:30288]
DUT	KNOWN	deoxyuridine triphosphatase [Source:HGNC Symbol;Acc:HGNC:3078]
E2F1	KNOWN	E2F transcription factor 1 [Source:HGNC Symbol;Acc:HGNC:3113]
EFNA2	KNOWN	ephrin-A2 [Source:HGNC Symbol;Acc:HGNC:3222]
EIF4EBP1	KNOWN	eukaryotic translation initiation factor 4E binding protein 1 [Source:HGNC Symbol;Acc:HGNC:3288]
ELMOD1	KNOWN	ELMO/CED-12 domain containing 1 [Source:HGNC Symbol;Acc:HGNC:25334]
ENOSF1	KNOWN	enolase superfamily member 1 [Source:HGNC Symbol;Acc:HGNC:30365]
EPHB6	KNOWN	EPH receptor B6 [Source:HGNC Symbol;Acc:HGNC:3396]
ERCC6L	KNOWN	excision repair cross-complementation group 6-like [Source:HGNC Symbol;Acc:HGNC:20794]
ESCO2	KNOWN	establishment of sister chromatid cohesion N-acetyltransferase 2 [Source:HGNC Symbol;Acc:HGNC:27230]
ESPL1	KNOWN	extra spindle pole bodies homolog 1 (S. cerevisiae) [Source:HGNC Symbol;Acc:HGNC:16856]
ETV4	KNOWN	ets variant 4 [Source:HGNC Symbol;Acc:HGNC:3493]
EXO1	KNOWN	exonuclease 1 [Source:HGNC Symbol;Acc:HGNC:3511]
EZH2	KNOWN	enhancer of zeste 2 polycomb repressive complex 2 subunit [Source:HGNC Symbol;Acc:HGNC:3527]
EZH2	KNOWN	enhancer of zeste 2 polycomb repressive complex 2 subunit [Source:HGNC Symbol;Acc:HGNC:3527]
FABP4	KNOWN	fatty acid binding protein 4, adipocyte [Source:HGNC Symbol;Acc:HGNC:3559]
FAM111B	KNOWN	family with sequence similarity 111, member B [Source:HGNC Symbol;Acc:HGNC:24200]
FAM172A	KNOWN	family with sequence similarity 172, member A [Source:HGNC Symbol;Acc:HGNC:25365]
FAM173A	KNOWN	family with sequence similarity 173, member A [Source:HGNC Symbol;Acc:HGNC:14152]
FAM216A	KNOWN	family with sequence similarity 216, member A [Source:HGNC Symbol;Acc:HGNC:30180]
FAM64A	KNOWN	family with sequence similarity 64, member A [Source:HGNC Symbol;Acc:HGNC:25483]
FAM83D	KNOWN	family with sequence similarity 83, member D [Source:HGNC Symbol;Acc:HGNC:16122]
FANCA	KNOWN	Fanconi anemia, complementation group A [Source:HGNC Symbol;Acc:HGNC:3582]
FANCA	KNOWN	Fanconi anemia, complementation group A [Source:HGNC Symbol;Acc:HGNC:3582]
FANCB	KNOWN	Fanconi anemia, complementation group B [Source:HGNC Symbol;Acc:HGNC:3583]
FANCB	KNOWN	Fanconi anemia, complementation group B [Source:HGNC Symbol;Acc:HGNC:3583]
FANCD2	KNOWN	Fanconi anemia, complementation group D2 [Source:HGNC Symbol;Acc:HGNC:3585]
FANCE	KNOWN	Fanconi anemia, complementation group E [Source:HGNC Symbol;Acc:HGNC:3586]
FANCE	KNOWN	Fanconi anemia, complementation group E [Source:HGNC Symbol;Acc:HGNC:3586]
FANCI	KNOWN	Fanconi anemia, complementation group I [Source:HGNC Symbol;Acc:HGNC:25568]
FANCI	KNOWN	Fanconi anemia, complementation group I [Source:HGNC Symbol;Acc:HGNC:25568]
FBL	KNOWN	fibrillarin [Source:HGNC Symbol;Acc:HGNC:3599]
FBXL16	KNOWN	F-box and leucine-rich repeat protein 16 [Source:HGNC Symbol;Acc:HGNC:14150]
FBXO5	KNOWN	F-box protein 5 [Source:HGNC Symbol;Acc:HGNC:13584]
FEN1	KNOWN	flap structure-specific endonuclease 1 [Source:HGNC Symbol;Acc:HGNC:3650]
FGD4	KNOWN	FYVE, RhoGEF and PH domain containing 4 [Source:HGNC Symbol;Acc:HGNC:19125]
FKBP11	KNOWN	FK506 binding protein 11, 19 kDa [Source:HGNC Symbol;Acc:HGNC:18624]
FOXM1	KNOWN	forkhead box M1 [Source:HGNC Symbol;Acc:HGNC:3818]
FUS	KNOWN	FUS RNA binding protein [Source:HGNC Symbol;Acc:HGNC:4010]
GDF6	KNOWN	growth differentiation factor 6 [Source:HGNC Symbol;Acc:HGNC:4221]
GINS1	KNOWN	GINS complex subunit 1 (Psf1 homolog) [Source:HGNC Symbol;Acc:HGNC:28980]
GINS2	KNOWN	GINS complex subunit 2 (Psf2 homolog) [Source:HGNC Symbol;Acc:HGNC:24575]

Supplementary Table 8 - continued

Associated Gene Name	Status (gene)	Description
GINS4	KNOWN	GINS complex subunit 4 (Sld5 homolog) [Source:HGNC Symbol;Acc:HGNC:28226]
GJA4	KNOWN	gap junction protein, alpha 4, 37kDa [Source:HGNC Symbol;Acc:HGNC:4278]
GMNN	KNOWN	geminin, DNA replication inhibitor [Source:HGNC Symbol;Acc:HGNC:17493]
GPC2	KNOWN	glypican 2 [Source:HGNC Symbol;Acc:HGNC:4450]
GPRC5B	KNOWN	G protein-coupled receptor, class C, group 5, member B [Source:HGNC Symbol;Acc:HGNC:13308]
GPX7	KNOWN	glutathione peroxidase 7 [Source:HGNC Symbol;Acc:HGNC:4559]
GTSE1	KNOWN	G-2 and S-phase expressed 1 [Source:HGNC Symbol;Acc:HGNC:13698]
HOMER3	KNOWN	homer homolog 3 (Drosophila) [Source:HGNC Symbol;Acc:HGNC:17514]
H2AFX	KNOWN	H2A histone family, member X [Source:HGNC Symbol;Acc:HGNC:4739]
H2AFZ	KNOWN	H2A histone family, member Z [Source:HGNC Symbol;Acc:HGNC:4741]
HAUS8	KNOWN	HAUS augmin-like complex, subunit 8 [Source:HGNC Symbol;Acc:HGNC:30532]
HELLS	KNOWN	helicase, lymphoid-specific [Source:HGNC Symbol;Acc:HGNC:4861]
HIC1	KNOWN	hypermethylated in cancer 1 [Source:HGNC Symbol;Acc:HGNC:4909]
HJURP	KNOWN	Holliday junction recognition protein [Source:HGNC Symbol;Acc:HGNC:25444]
HMGB1	KNOWN	high mobility group box 1 [Source:HGNC Symbol;Acc:HGNC:4983]
HMGB2	KNOWN	high mobility group box 2 [Source:HGNC Symbol;Acc:HGNC:5000]
HMGN2	KNOWN	high mobility group nucleosomal binding domain 2 [Source:HGNC Symbol;Acc:HGNC:4986]
HMMR	KNOWN	hyaluronan-mediated motility receptor (RHAMM) [Source:HGNC Symbol;Acc:HGNC:5012]
HNRNPA1	KNOWN	heterogeneous nuclear ribonucleoprotein A1 [Source:HGNC Symbol;Acc:HGNC:5031]
HNRNPA1P10	KNOWN	heterogeneous nuclear ribonucleoprotein A1 pseudogene 10 [Source:HGNC Symbol;Acc:HGNC:39128]
HNRNPD	KNOWN	heterogeneous nuclear ribonucleoprotein D (AU-rich element RNA binding protein 1, 37kDa) [Source:HGNC Symbol;Acc:HGNC:5036]
HNRNPM	KNOWN	heterogeneous nuclear ribonucleoprotein M [Source:HGNC Symbol;Acc:HGNC:5046]
HOXA3	KNOWN	homeobox A3 [Source:HGNC Symbol;Acc:HGNC:5104]
HTR1B	KNOWN	5-hydroxytryptamine (serotonin) receptor 1B, G protein-coupled [Source:HGNC Symbol;Acc:HGNC:5287]
IQGAP3	KNOWN	IQ motif containing GTPase activating protein 3 [Source:HGNC Symbol;Acc:HGNC:20669]
ISOC1	KNOWN	isochorismatase domain containing 1 [Source:HGNC Symbol;Acc:HGNC:24254]
KIAA0101	KNOWN	KIAA0101 [Source:HGNC Symbol;Acc:HGNC:28961]
KIAA1524	KNOWN	KIAA1524 [Source:HGNC Symbol;Acc:HGNC:29302]
KIF11	KNOWN	kinesin family member 11 [Source:HGNC Symbol;Acc:HGNC:6388]
KIF14	KNOWN	kinesin family member 14 [Source:HGNC Symbol;Acc:HGNC:19181]
KIF18A	KNOWN	kinesin family member 18A [Source:HGNC Symbol;Acc:HGNC:29441]
CAMKK2	KNOWN	calcium/calmodulin-dependent protein kinase kinase 2, beta [Source:HGNC Symbol;Acc:HGNC:1470]
CXCL3	KNOWN	chemokine (C-X-C motif) ligand 3 [Source:HGNC Symbol;Acc:HGNC:4604]
KIF18B	KNOWN	kinesin family member 18B [Source:HGNC Symbol;Acc:HGNC:27102]
KIF20A	KNOWN	kinesin family member 20A [Source:HGNC Symbol;Acc:HGNC:9787]
KIF20B	KNOWN	kinesin family member 20B [Source:HGNC Symbol;Acc:HGNC:7212]
KIF22	KNOWN	kinesin family member 22 [Source:HGNC Symbol;Acc:HGNC:6391]
KIF23	KNOWN	kinesin family member 23 [Source:HGNC Symbol;Acc:HGNC:6392]
KIF2C	KNOWN	kinesin family member 2C [Source:HGNC Symbol;Acc:HGNC:6393]
KIF4A	KNOWN	kinesin family member 4A [Source:HGNC Symbol;Acc:HGNC:13339]
KIT	KNOWN	v-kit Hardy-Zuckerman 4 feline sarcoma viral oncogene homolog [Source:HGNC Symbol;Acc:HGNC:6342]
KIT	KNOWN	v-kit Hardy-Zuckerman 4 feline sarcoma viral oncogene homolog [Source:HGNC Symbol;Acc:HGNC:6342]
KLHL23	KNOWN	kelch-like family member 23 [Source:HGNC Symbol;Acc:HGNC:27506]
KNTC1	KNOWN	kinetochore associated 1 [Source:HGNC Symbol;Acc:HGNC:17255]
KRT18	KNOWN	keratin 18 [Source:HGNC Symbol;Acc:HGNC:6430]
KRT7	KNOWN	keratin 7 [Source:HGNC Symbol;Acc:HGNC:6445]
KRT80	KNOWN	keratin 80 [Source:HGNC Symbol;Acc:HGNC:27056]
LINC00176	KNOWN	long intergenic non-protein coding RNA 176 [Source:HGNC Symbol;Acc:HGNC:27655]
LMNB1	KNOWN	lamin B1 [Source:HGNC Symbol;Acc:HGNC:6637]
LOXL2	KNOWN	lysyl oxidase-like 2 [Source:HGNC Symbol;Acc:HGNC:6666]
LRRC17	KNOWN	leucine rich repeat containing 17 [Source:HGNC Symbol;Acc:HGNC:16895]
LXN	KNOWN	latexin [Source:HGNC Symbol;Acc:HGNC:13347]
MAD2L1	KNOWN	MAD2 mitotic arrest deficient-like 1 (yeast) [Source:HGNC Symbol;Acc:HGNC:6763]
MDM1	KNOWN	Mdm1 nuclear protein homolog (mouse) [Source:HGNC Symbol;Acc:HGNC:29917]
MAP2K6	KNOWN	mitogen-activated protein kinase kinase 6 [Source:HGNC Symbol;Acc:HGNC:6846]
MCM2	KNOWN	minichromosome maintenance complex component 2 [Source:HGNC Symbol;Acc:HGNC:6944]
MCM4	KNOWN	minichromosome maintenance complex component 4 [Source:HGNC Symbol;Acc:HGNC:6947]
MCM5	KNOWN	minichromosome maintenance complex component 5 [Source:HGNC Symbol;Acc:HGNC:6948]
MCM6	KNOWN	minichromosome maintenance complex component 6 [Source:HGNC Symbol;Acc:HGNC:6949]
MCM7	KNOWN	minichromosome maintenance complex component 7 [Source:HGNC Symbol;Acc:HGNC:6950]
MCM8	KNOWN	minichromosome maintenance complex component 8 [Source:HGNC Symbol;Acc:HGNC:16147]
MDK	KNOWN	midkine (neurite growth-promoting factor 2) [Source:HGNC Symbol;Acc:HGNC:6972]
MELK	KNOWN	maternal embryonic leucine zipper kinase [Source:HGNC Symbol;Acc:HGNC:16870]
MGP	KNOWN	matrix Gla protein [Source:HGNC Symbol;Acc:HGNC:7060]
MIF	KNOWN	macrophage migration inhibitory factor (glycosylation-inhibiting factor) [Source:HGNC Symbol;Acc:HGNC:7097]
MIR1178	KNOWN	microRNA 1178 [Source:HGNC Symbol;Acc:HGNC:35259]
MIR2277	KNOWN	microRNA 2277 [Source:HGNC Symbol;Acc:HGNC:37314]
MIR3917	KNOWN	microRNA 3917 [Source:HGNC Symbol;Acc:HGNC:38911]
MIS18A	KNOWN	MIS18 kinetochore protein A [Source:HGNC Symbol;Acc:HGNC:1286]
MKI67	KNOWN	marker of proliferation Ki-67 [Source:HGNC Symbol;Acc:HGNC:7107]
MOV10L1	KNOWN	Mov10l1, Moloney leukemia virus 10-like 1, homolog (mouse) [Source:HGNC Symbol;Acc:HGNC:7201]
MRPL3	KNOWN	mitochondrial ribosomal protein L3 [Source:HGNC Symbol;Acc:HGNC:10379]
MSMP	KNOWN	microseminoprotein, prostate associated [Source:HGNC Symbol;Acc:HGNC:29663]
MT1E	KNOWN	metallothionein 1E [Source:HGNC Symbol;Acc:HGNC:7397]
MT1L	KNOWN	metallothionein 1L (gene/pseudogene) [Source:HGNC Symbol;Acc:HGNC:7404]

Supplementary Table 8 - continued

Associated Gene Name	Status (gene)	Description
MT1X	KNOWN	metallothionein 1X [Source:HGNC Symbol;Acc:HGNC:7405]
MTBP	KNOWN	MDM2 binding protein [Source:HGNC Symbol;Acc:HGNC:7417]
MTHFD1	KNOWN	*methylenetetrahydrofolate dehydrogenase (NADP+ dependent) 1, methenyltetrahydrofolate cyclohydrolase, formyltetrahydrofolate syn
MYBL1	KNOWN	v-myb avian myeloblastosis viral oncogene homolog-like 1 [Source:HGNC Symbol;Acc:HGNC:7547]
MYBL2	KNOWN	v-myb avian myeloblastosis viral oncogene homolog-like 2 [Source:HGNC Symbol;Acc:HGNC:7548]
MYCN	KNOWN	v-myc avian myelocytomatosis viral oncogene neuroblastoma derived homolog [Source:HGNC Symbol;Acc:HGNC:7559]
MYH10	KNOWN	myosin, heavy chain 10, non-muscle [Source:HGNC Symbol;Acc:HGNC:7568]
MYLK2	KNOWN	myosin light chain kinase 2 [Source:HGNC Symbol;Acc:HGNC:16243]
MYLK2	KNOWN	myosin light chain kinase 2 [Source:HGNC Symbol;Acc:HGNC:16243]
MYO1B	KNOWN	myosin IB [Source:HGNC Symbol;Acc:HGNC:7596]
NASP	KNOWN	nuclear autoantigenic sperm protein (histone-binding) [Source:HGNC Symbol;Acc:HGNC:7644]
NCAPD2	KNOWN	non-SMC condensin I complex, subunit D2 [Source:HGNC Symbol;Acc:HGNC:24305]
NCAPD3	KNOWN	non-SMC condensin II complex, subunit D3 [Source:HGNC Symbol;Acc:HGNC:28952]
NCAPG	KNOWN	non-SMC condensin I complex, subunit G [Source:HGNC Symbol;Acc:HGNC:24304]
NCAPG2	KNOWN	non-SMC condensin II complex, subunit G2 [Source:HGNC Symbol;Acc:HGNC:21904]
NCAPH	KNOWN	non-SMC condensin I complex, subunit H [Source:HGNC Symbol;Acc:HGNC:1112]
NDC80	KNOWN	NDC80 kinetochore complex component [Source:HGNC Symbol;Acc:HGNC:16909]
NEIL3	KNOWN	nei endonuclease VIII-like 3 (E. coli) [Source:HGNC Symbol;Acc:HGNC:24573]
NEK2	KNOWN	NIMA-related kinase 2 [Source:HGNC Symbol;Acc:HGNC:7745]
NPM1	KNOWN	nucleophosmin (nucleolar phosphoprotein B23, numatrin) [Source:HGNC Symbol;Acc:HGNC:7910]
NPM1	KNOWN	nucleophosmin (nucleolar phosphoprotein B23, numatrin) [Source:HGNC Symbol;Acc:HGNC:7910]
NRARP	KNOWN	NOTCH-regulated ankyrin repeat protein [Source:HGNC Symbol;Acc:HGNC:33843]
NUDT11	KNOWN	nudix (nucleoside diphosphate linked moiety X)-type motif 11 [Source:HGNC Symbol;Acc:HGNC:18011]
NUF2	KNOWN	NUF2, NDC80 kinetochore complex component [Source:HGNC Symbol;Acc:HGNC:14621]
NUP35	KNOWN	nucleoporin 35kDa [Source:HGNC Symbol;Acc:HGNC:29797]
NUSAP1	KNOWN	nucleolar and spindle associated protein 1 [Source:HGNC Symbol;Acc:HGNC:18538]
OBSL1	KNOWN	obscurin-like 1 [Source:HGNC Symbol;Acc:HGNC:29092]
OIP5	KNOWN	Opa interacting protein 5 [Source:HGNC Symbol;Acc:HGNC:20300]
ORC1	KNOWN	origin recognition complex, subunit 1 [Source:HGNC Symbol;Acc:HGNC:8487]
ORC6	KNOWN	origin recognition complex, subunit 6 [Source:HGNC Symbol;Acc:HGNC:17151]
PACSIN3	KNOWN	protein kinase C and casein kinase substrate in neurons 3 [Source:HGNC Symbol;Acc:HGNC:8572]
PAFAH1B3	KNOWN	platelet-activating factor acetylhydrolase 1b, catalytic subunit 3 (29kDa) [Source:HGNC Symbol;Acc:HGNC:8576]
PAICS	KNOWN	**phosphoribosylaminoimidazole carboxylase, phosphoribosylaminoimidazole succinocarboxamide synthetase [Source:HGNC Symbol;Acc
PAK6	KNOWN	p21 protein (Cdc42/Rac)-activated kinase 6 [Source:HGNC Symbol;Acc:HGNC:16061]
PARD6G	KNOWN	par-6 family cell polarity regulator gamma [Source:HGNC Symbol;Acc:HGNC:16076]
PARPBP	KNOWN	PARP1 binding protein [Source:HGNC Symbol;Acc:HGNC:26074]
PBK	KNOWN	PDZ binding kinase [Source:HGNC Symbol;Acc:HGNC:18282]
PCNA	KNOWN	proliferating cell nuclear antigen [Source:HGNC Symbol;Acc:HGNC:8729]
PDGFB	KNOWN	platelet-derived growth factor beta polypeptide [Source:HGNC Symbol;Acc:HGNC:8800]
PDLIM1	KNOWN	PDZ and LIM domain 1 [Source:HGNC Symbol;Acc:HGNC:2067]
PEG10	KNOWN	paternally expressed 10 [Source:HGNC Symbol;Acc:HGNC:14005]
PGF	KNOWN	placental growth factor [Source:HGNC Symbol;Acc:HGNC:8893]
PHGDH	KNOWN	phosphoglycerate dehydrogenase [Source:HGNC Symbol;Acc:HGNC:8923]
PHOSPHO2	KNOWN	phosphatase, orphan 2 [Source:HGNC Symbol;Acc:HGNC:28316]
PIM3	KNOWN	pim-3 oncogene [Source:HGNC Symbol;Acc:HGNC:19310]
PKMYT1	KNOWN	protein kinase, membrane associated tyrosine/threonine 1 [Source:HGNC Symbol;Acc:HGNC:29650]
PLK1	KNOWN	polo-like kinase 1 [Source:HGNC Symbol;Acc:HGNC:9077]
PLK4	KNOWN	polo-like kinase 4 [Source:HGNC Symbol;Acc:HGNC:11397]
POC1A	KNOWN	POC1 centriolar protein A [Source:HGNC Symbol;Acc:HGNC:24488]
POLA1	KNOWN	polymerase (DNA directed), alpha 1, catalytic subunit [Source:HGNC Symbol;Acc:HGNC:9173]
POLD1	KNOWN	polymerase (DNA directed), delta 1, catalytic subunit [Source:HGNC Symbol;Acc:HGNC:9175]
POLE	KNOWN	polymerase (DNA directed), epsilon, catalytic subunit [Source:HGNC Symbol;Acc:HGNC:9177]
POLE2	KNOWN	polymerase (DNA directed), epsilon 2, accessory subunit [Source:HGNC Symbol;Acc:HGNC:9178]
POLQ	KNOWN	polymerase (DNA directed), theta [Source:HGNC Symbol;Acc:HGNC:9186]
POLR3G	KNOWN	polymerase (RNA) III (DNA directed) polypeptide G (32kD) [Source:HGNC Symbol;Acc:HGNC:30075]
PRC1	KNOWN	protein regulator of cytokinesis 1 [Source:HGNC Symbol;Acc:HGNC:9341]
PRIM1	KNOWN	primase, DNA, polypeptide 1 (49kDa) [Source:HGNC Symbol;Acc:HGNC:9369]
PRIM2	KNOWN	primase, DNA, polypeptide 2 (58kDa) [Source:HGNC Symbol;Acc:HGNC:9370]
PRMT1	KNOWN	protein arginine methyltransferase 1 [Source:HGNC Symbol;Acc:HGNC:5187]
PRR11	KNOWN	proline rich 11 [Source:HGNC Symbol;Acc:HGNC:25619]
PRSS12	KNOWN	protease, serine, 12 (neurotrypsin, motopsin) [Source:HGNC Symbol;Acc:HGNC:9477]
PSMC3IP	KNOWN	PSMC3 interacting protein [Source:HGNC Symbol;Acc:HGNC:17928]
PTTG1	KNOWN	pituitary tumor-transforming 1 [Source:HGNC Symbol;Acc:HGNC:9690]
RACGAP1	KNOWN	Rac GTPase activating protein 1 [Source:HGNC Symbol;Acc:HGNC:9804]
RAD51	KNOWN	RAD51 recombinase [Source:HGNC Symbol;Acc:HGNC:9817]
RAD51AP1	KNOWN	RAD51 associated protein 1 [Source:HGNC Symbol;Acc:HGNC:16956]
RANBP1	KNOWN	RAN binding protein 1 [Source:HGNC Symbol;Acc:HGNC:9847]
RBL1	KNOWN	retinoblastoma-like 1 [Source:HGNC Symbol;Acc:HGNC:9893]
RCC1	KNOWN	regulator of chromosome condensation 1 [Source:HGNC Symbol;Acc:HGNC:1913]
RECQL4	KNOWN	RecQ protein-like 4 [Source:HGNC Symbol;Acc:HGNC:9949]
RFC3	KNOWN	replication factor C (activator 1) 3, 38kDa [Source:HGNC Symbol;Acc:HGNC:9971]
RFC5	KNOWN	replication factor C (activator 1) 5, 36.5kDa [Source:HGNC Symbol;Acc:HGNC:9973]
RGS4	KNOWN	regulator of G-protein signaling 4 [Source:HGNC Symbol;Acc:HGNC:10000]
RMI2	KNOWN	RecQ mediated genome instability 2 [Source:HGNC Symbol;Acc:HGNC:28349]
RNASEH2A	KNOWN	ribonuclease H2, subunit A [Source:HGNC Symbol;Acc:HGNC:18518]

Supplementary Table 8 - continued

Associated Gene Name	Status (gene)	Description
RNASEH2A	KNOWN	ribonuclease H2, subunit A [Source:HGNC Symbol;Acc:HGNC:18518]
RPL22L1	KNOWN	ribosomal protein L22-like 1 [Source:HGNC Symbol;Acc:HGNC:27610]
RPS6KA1	KNOWN	ribosomal protein S6 kinase, 90kDa, polypeptide 1 [Source:HGNC Symbol;Acc:HGNC:10430]
RRM1	KNOWN	ribonucleotide reductase M1 [Source:HGNC Symbol;Acc:HGNC:10451]
RRM2	KNOWN	ribonucleotide reductase M2 [Source:HGNC Symbol;Acc:HGNC:10452]
SAPCD2	KNOWN	suppressor APC domain containing 2 [Source:HGNC Symbol;Acc:HGNC:28055]
SEMA6C	KNOWN	sema domain, transmembrane domain (TM), and cytoplasmic domain, (semaphorin) 6C [Source:HGNC Symbol;Acc:HGNC:10740]
SGOL1	KNOWN	shugoshin-like 1 (S. pombe) [Source:HGNC Symbol;Acc:HGNC:25088]
SGOL2	KNOWN	shugoshin-like 2 (S. pombe) [Source:HGNC Symbol;Acc:HGNC:30812]
SHCBP1	KNOWN	SHC SH2-domain binding protein 1 [Source:HGNC Symbol;Acc:HGNC:29547]
SHF	KNOWN	Src homology 2 domain containing F [Source:HGNC Symbol;Acc:HGNC:25116]
SHMT1	KNOWN	serine hydroxymethyltransferase 1 (soluble) [Source:HGNC Symbol;Acc:HGNC:10850]
SIK1	KNOWN	salt-inducible kinase 1 [Source:HGNC Symbol;Acc:HGNC:11142]
SKA1	KNOWN	spindle and kinetochore associated complex subunit 1 [Source:HGNC Symbol;Acc:HGNC:28109]
SKA1	KNOWN	spindle and kinetochore associated complex subunit 1 [Source:HGNC Symbol;Acc:HGNC:28109]
SKA3	KNOWN	spindle and kinetochore associated complex subunit 3 [Source:HGNC Symbol;Acc:HGNC:20262]
SLC17A9	KNOWN	solute carrier family 17 (vesicular nucleotide transporter), member 9 [Source:HGNC Symbol;Acc:HGNC:16192]
SLC19A1	KNOWN	solute carrier family 19 (folate transporter), member 1 [Source:HGNC Symbol;Acc:HGNC:10937]
SLC25A10	KNOWN	solute carrier family 25 (mitochondrial carrier; dicarboxylate transporter), member 10 [Source:HGNC Symbol;Acc:HGNC:10980]
SLC29A4	KNOWN	solute carrier family 29 (equilibrative nucleoside transporter), member 4 [Source:HGNC Symbol;Acc:HGNC:23097]
SLC7A2	KNOWN	solute carrier family 7 (cationic amino acid transporter, y+ system), member 2 [Source:HGNC Symbol;Acc:HGNC:11060]
SMC4	KNOWN	structural maintenance of chromosomes 4 [Source:HGNC Symbol;Acc:HGNC:14013]
SMO	KNOWN	smoothened, frizzled class receptor [Source:HGNC Symbol;Acc:HGNC:11119]
SNHG3	KNOWN	small nucleolar RNA host gene 3 (non-protein coding) [Source:HGNC Symbol;Acc:HGNC:10118]
SNRPA	KNOWN	small nuclear ribonucleoprotein polypeptide A [Source:HGNC Symbol;Acc:HGNC:11151]
SNTB1	KNOWN	syntrophin, beta 1 (dystrophin-associated protein A1, 59kDa, basic component 1) [Source:HGNC Symbol;Acc:HGNC:11168]
EPGN	KNOWN	epithelial mitogen [Source:HGNC Symbol;Acc:HGNC:17470]
SOCS3	KNOWN	suppressor of cytokine signaling 3 [Source:HGNC Symbol;Acc:HGNC:19391]
SOX18	KNOWN	SRY (sex determining region Y)-box 18 [Source:HGNC Symbol;Acc:HGNC:11194]
SPAG5	KNOWN	sperm associated antigen 5 [Source:HGNC Symbol;Acc:HGNC:13452]
SPC24	KNOWN	SPC24, NDC80 kinetochore complex component [Source:HGNC Symbol;Acc:HGNC:26913]
SPC25	KNOWN	SPC25, NDC80 kinetochore complex component [Source:HGNC Symbol;Acc:HGNC:24031]
SPC25	KNOWN	SPC25, NDC80 kinetochore complex component [Source:HGNC Symbol;Acc:HGNC:24031]
STIL	KNOWN	SCL/TAL1 interrupting locus [Source:HGNC Symbol;Acc:HGNC:10879]
STMN1	KNOWN	stathmin 1 [Source:HGNC Symbol;Acc:HGNC:6510]
SUPT3H	KNOWN	suppressor of Ty 3 homolog (S. cerevisiae) [Source:HGNC Symbol;Acc:HGNC:11466]
TACC3	KNOWN	transforming, acidic coiled-coil containing protein 3 [Source:HGNC Symbol;Acc:HGNC:11524]
TCF19	KNOWN	transcription factor 19 [Source:HGNC Symbol;Acc:HGNC:11629]
TCF19	KNOWN	transcription factor 19 [Source:HGNC Symbol;Acc:HGNC:11629]
TCF19	KNOWN	transcription factor 19 [Source:HGNC Symbol;Acc:HGNC:11629]
TCF19	KNOWN	transcription factor 19 [Source:HGNC Symbol;Acc:HGNC:11629]
TCF19	KNOWN	transcription factor 19 [Source:HGNC Symbol;Acc:HGNC:11629]
TCF19	KNOWN	transcription factor 19 [Source:HGNC Symbol;Acc:HGNC:11629]
TCF19	KNOWN	transcription factor 19 [Source:HGNC Symbol;Acc:HGNC:11629]
TCF7	KNOWN	transcription factor 7 (T-cell specific, HMG-box) [Source:HGNC Symbol;Acc:HGNC:11639]
THSD7A	KNOWN	thrombospondin, type I, domain containing 7A [Source:HGNC Symbol;Acc:HGNC:22207]
TK1	KNOWN	thymidine kinase 1, soluble [Source:HGNC Symbol;Acc:HGNC:11830]
TMEM71	KNOWN	transmembrane protein 71 [Source:HGNC Symbol;Acc:HGNC:26572]
TMEM97	KNOWN	transmembrane protein 97 [Source:HGNC Symbol;Acc:HGNC:28106]
TMPO	KNOWN	thymopoietin [Source:HGNC Symbol;Acc:HGNC:11875]
TNR	KNOWN	tenascin R [Source:HGNC Symbol;Acc:HGNC:11953]
TOP2A	KNOWN	topoisomerase (DNA) II alpha 170kDa [Source:HGNC Symbol;Acc:HGNC:11989]
TOX	KNOWN	thymocyte selection-associated high mobility group box [Source:HGNC Symbol;Acc:HGNC:18988]
TPX2	KNOWN	TPX2, microtubule-associated [Source:HGNC Symbol;Acc:HGNC:1249]
TRAIP	KNOWN	TRAF interacting protein [Source:HGNC Symbol;Acc:HGNC:30764]
TRIP13	KNOWN	thyroid hormone receptor interactor 13 [Source:HGNC Symbol;Acc:HGNC:12307]
TROAP	KNOWN	trophinin associated protein [Source:HGNC Symbol;Acc:HGNC:12327]
TSPAN13	KNOWN	tetraspanin 13 [Source:HGNC Symbol;Acc:HGNC:21643]
TTF2	KNOWN	transcription termination factor, RNA polymerase II [Source:HGNC Symbol;Acc:HGNC:12398]
TTK	KNOWN	TTK protein kinase [Source:HGNC Symbol;Acc:HGNC:12401]
TTYH2	KNOWN	tweety family member 2 [Source:HGNC Symbol;Acc:HGNC:13877]
TUBA1B	KNOWN	tubulin, alpha 1b [Source:HGNC Symbol;Acc:HGNC:18809]
TYMS	KNOWN	thymidylate synthetase [Source:HGNC Symbol;Acc:HGNC:12441]
UBE2C	KNOWN	ubiquitin-conjugating enzyme E2C [Source:HGNC Symbol;Acc:HGNC:15937]
UBE2S	KNOWN	ubiquitin-conjugating enzyme E2S [Source:HGNC Symbol;Acc:HGNC:17895]
UBE2T	KNOWN	ubiquitin-conjugating enzyme E2T (putative) [Source:HGNC Symbol;Acc:HGNC:25009]
UHRF1	KNOWN	ubiquitin-like with PHD and ring finger domains 1 [Source:HGNC Symbol;Acc:HGNC:12556]
VASH1	KNOWN	vasohibin 1 [Source:HGNC Symbol;Acc:HGNC:19964]
VRK1	KNOWN	vaccinia related kinase 1 [Source:HGNC Symbol;Acc:HGNC:12718]
WDR4	KNOWN	WD repeat domain 4 [Source:HGNC Symbol;Acc:HGNC:12756]
WDR62	KNOWN	WD repeat domain 62 [Source:HGNC Symbol;Acc:HGNC:24502]
WDR76	KNOWN	WD repeat domain 76 [Source:HGNC Symbol;Acc:HGNC:25773]
WNK4	KNOWN	WNK lysine deficient protein kinase 4 [Source:HGNC Symbol;Acc:HGNC:14544]
XRCC2	KNOWN	X-ray repair complementing defective repair in Chinese hamster cells 2 [Source:HGNC Symbol;Acc:HGNC:12829]
ZNF367	KNOWN	zinc finger protein 367 [Source:HGNC Symbol;Acc:HGNC:18320]

Supplementary Table 8 - continued

Associated Gene Name	Status (gene)	Description
ZNF655	KNOWN	zinc finger protein 655 [Source:HGNC Symbol;Acc:HGNC:30899]
ZNF714	KNOWN	zinc finger protein 714 [Source:HGNC Symbol;Acc:HGNC:27124]
ZWINT	KNOWN	ZW10 interacting kinetochore protein [Source:HGNC Symbol;Acc:HGNC:13195]

*methylenetetrahydrofolate dehydrogenase (NADP+ dependent) 1, methenyltetrahydrofolate cyclohydrolase, formyltetrahydrofolate synthetase [Source:HGNC Symbol;Acc:HGNC:7432]

**phosphoribosylaminoimidazole carboxylase, phosphoribosylaminoimidazole succinocarboxamide synthetase [Source:HGNC Symbol;Acc:HGNC:8587]

Supplementary Table 9. Biological Process Gene Ontology terms that were significantly overrepresented in the analysis of the list of the 103 genes upregulated 2 times or more by both EZH2-depletion and the exposure to FSS.

Biological Process	GO number:	Over- (+) or underrepresented (-)	p-value
cell adhesion	GO:0007155	+	8.17E-05
biological adhesion	GO:0022610	+	8.17E-05
blood coagulation	GO:0007596	+	1.16E-02
cellular component movement	GO:0006928	+	1.40E-02
Unclassified	-	-	1.59E-02
RNA metabolic process	GO:0016070	-	1.78E-02
cellular process	GO:0009987	+	2.47E-02
response to stimulus	GO:0050896	+	2.61E-02
response to external stimulus	GO:0009605	+	4.62E-02

Supplementary Table 10. Biological Process Gene Ontology terms that were significantly overrepresented in the analysis of the list of the 355 genes upregulated 2 times or more by both EZH2-depletion and the exposure to FSS.

Biological Process	GO number:	Over- (+) or underrepresented (-)	p-value
cell cycle	GO:0007049	+	1.77E-31
DNA metabolic process	GO:0006259	+	1.79E-22
DNA replication	GO:0006260	+	4.70E-18
mitosis	GO:0007067	+	4.01E-15
chromosome segregation	GO:0007059	+	3.32E-13
cellular process	GO:0009987	+	8.75E-12
cytokinesis	GO:0000910	+	2.58E-08
DNA repair	GO:0006281	+	2.54E-07
cellular component organization	GO:0016043	+	3.43E-06
cellular component organization or biogenesis	GO:0071840	+	2.04E-05
nucleobase-containing compound metabolic process	GO:0006139	+	2.24E-05
chromatin organization	GO:0006325	+	2.12E-03
meiosis	GO:0007126	+	4.82E-03
organelle organization	GO:0006996	+	6.49E-03
DNA recombination	GO:0006310	+	1.16E-02
metabolic process	GO:0008152	+	2.96E-02
