# Supplementary material for: The decrease in histone methyltransferase EZH2 in response to fluid shear stress alters endothelial gene expression and promotes quiescence
Source: Angiogenesis. 2015 Sep 28;19:9–24. doi: 10.1007/s10456-015-9485-2 (PMC4700080; doi:10.1007/s10456-015-9485-2)
Supplement: Supplementary file 2 — Supplementary material 2 (DOCX 4374 kb) [file 10456_2015_9485_MOESM2_ESM.docx]

**Supplementary Material**

**Online Figures and Figure Legends**

**
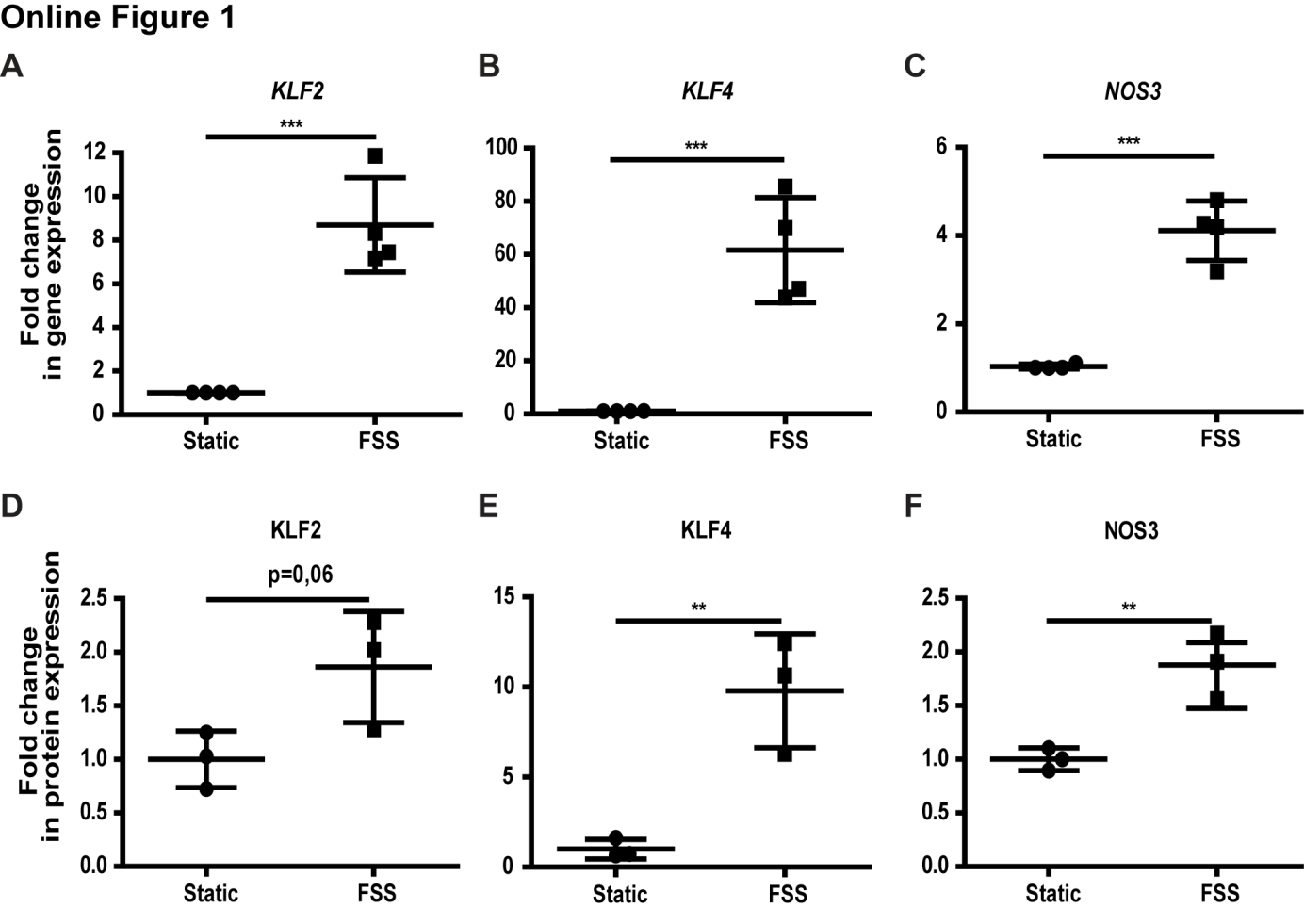
**

**Online Figure 1. FSS upregulates gene and protein expression of KLF2, KLF4 and NOS3.** Cells were exposed to laminar flow with FSS of 20 dyne/cm^2^ for 72h. **A – C** Gene expression of KLF2, KLF4 and NOS3, respectively, n=4, ***p<0.001, t-test. **D – F** Protein expression of KLF2, KLF4 and NOS3, respectively, n=3, **p<0.01, t-test.


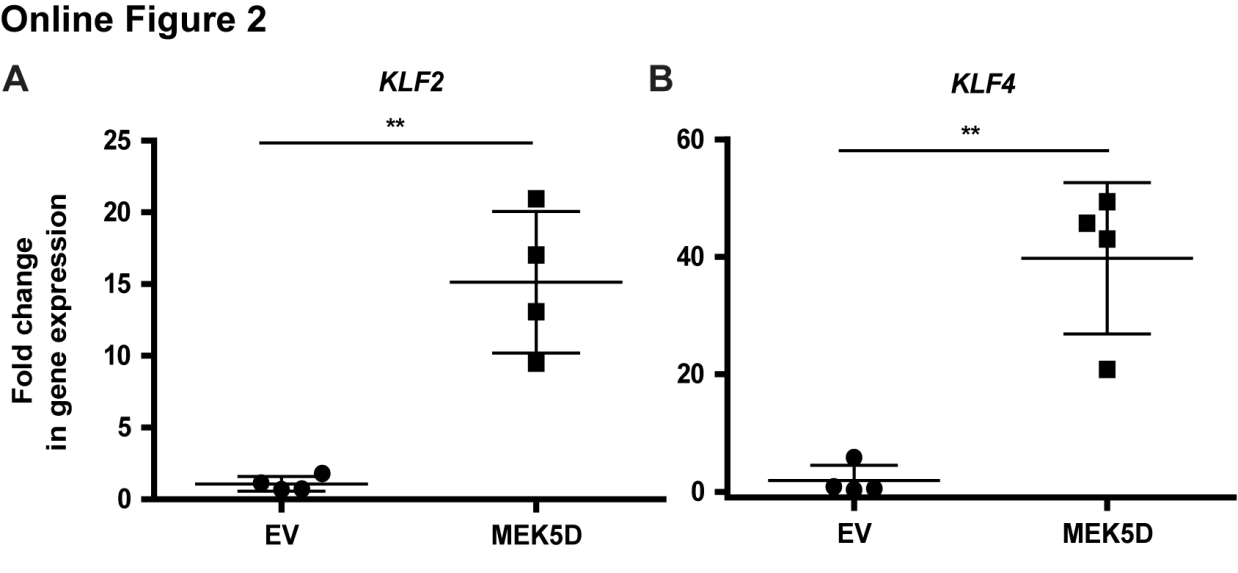


**Online Figure 2. Expression of the constitutively active MAP2K5 (MAP2K5D/MEK5D) leads to upregulation of MAPK7 target genes, confirming the increase in MAPK7 activity.** **A and B –** Gene expression of KLF2 and KLF4, respectively, n=4, **p<0.01, t-test.


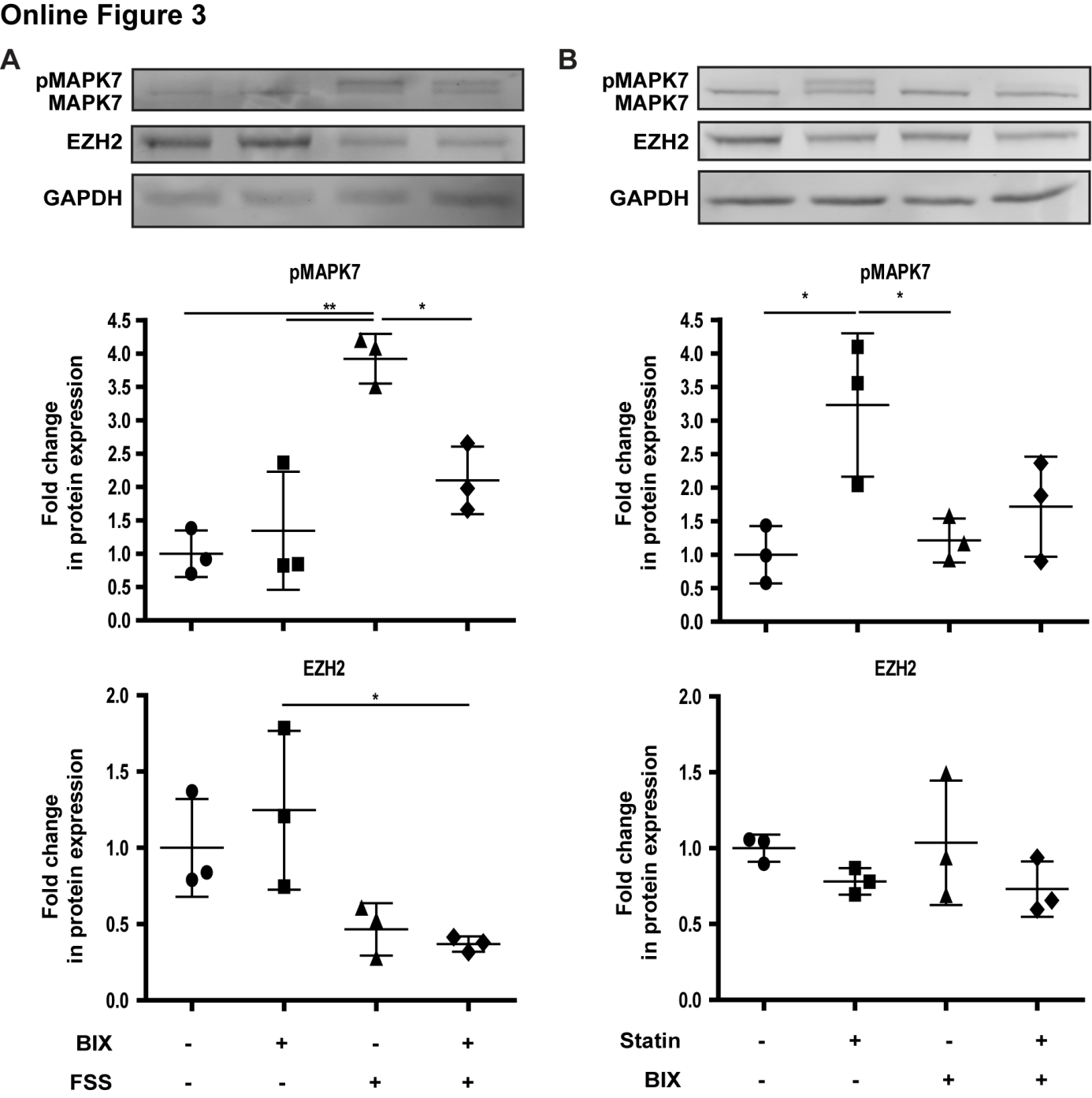


**Online Figure 3.**  **Inhibition of MAP2K5-MAPK7 (MEK5-Erk5) with BIX02189 does not rescue the decrease in EZH2 expression.** **A –** Representative Western blotting images (upper panel). Phosphorylated-MAPK7 (pMAPK7) and EZH2 protein expression derived through densitometry and normalized to GAPDH (lower panel). Cells were cultured under FSS for 72h, with or without 5 μM BIX02189 (BIX), n=3, *p<0.05, **p<0.001, 1-way ANOVA with Tukey *post hoc* comparisons between all pairs of means. **B –** Representative Western blotting images (upper panel). Phosphorylated-MAPK7 (pMAPK7) and EZH2 protein expression derived through densitometry and normalized to GAPDH (lower panel). Cells were treated for 24h with 1 μM simvastatin (statin) and/or 5 μM MAP2K5-MAPK7 inhibitor BIX02189 (BIX). *p<0.05, 1-way ANOVA with Tukey *post hoc* comparisons between all pairs of means.

**
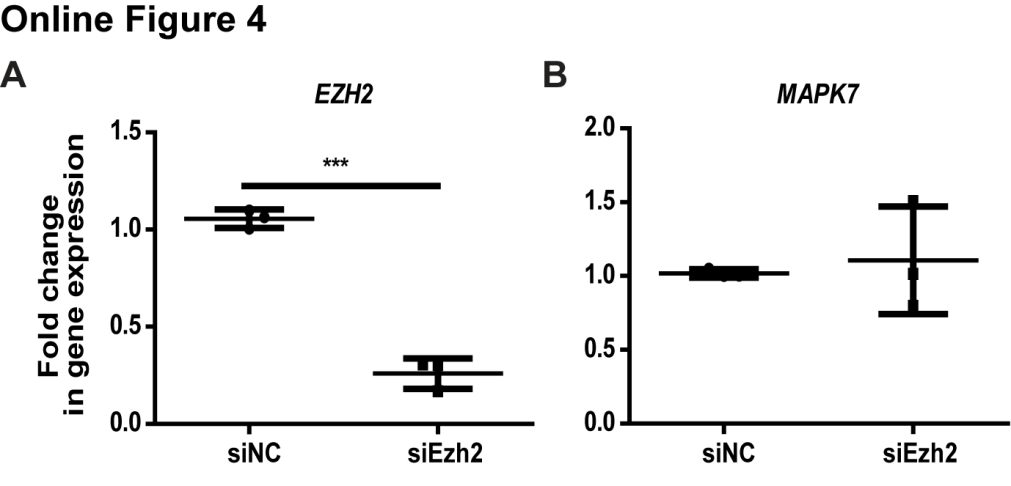
**

**Online Figure 4. The gene expression of MAPK7 is preserved upon knock-down of EZH2.** Cells were transfected with siRNA against EZH2 and analysed after 72h post-transfection. **A and B –** Gene expression of EZH2 and MAPK7, respectively, n=3, ***p<0.001, t-test.


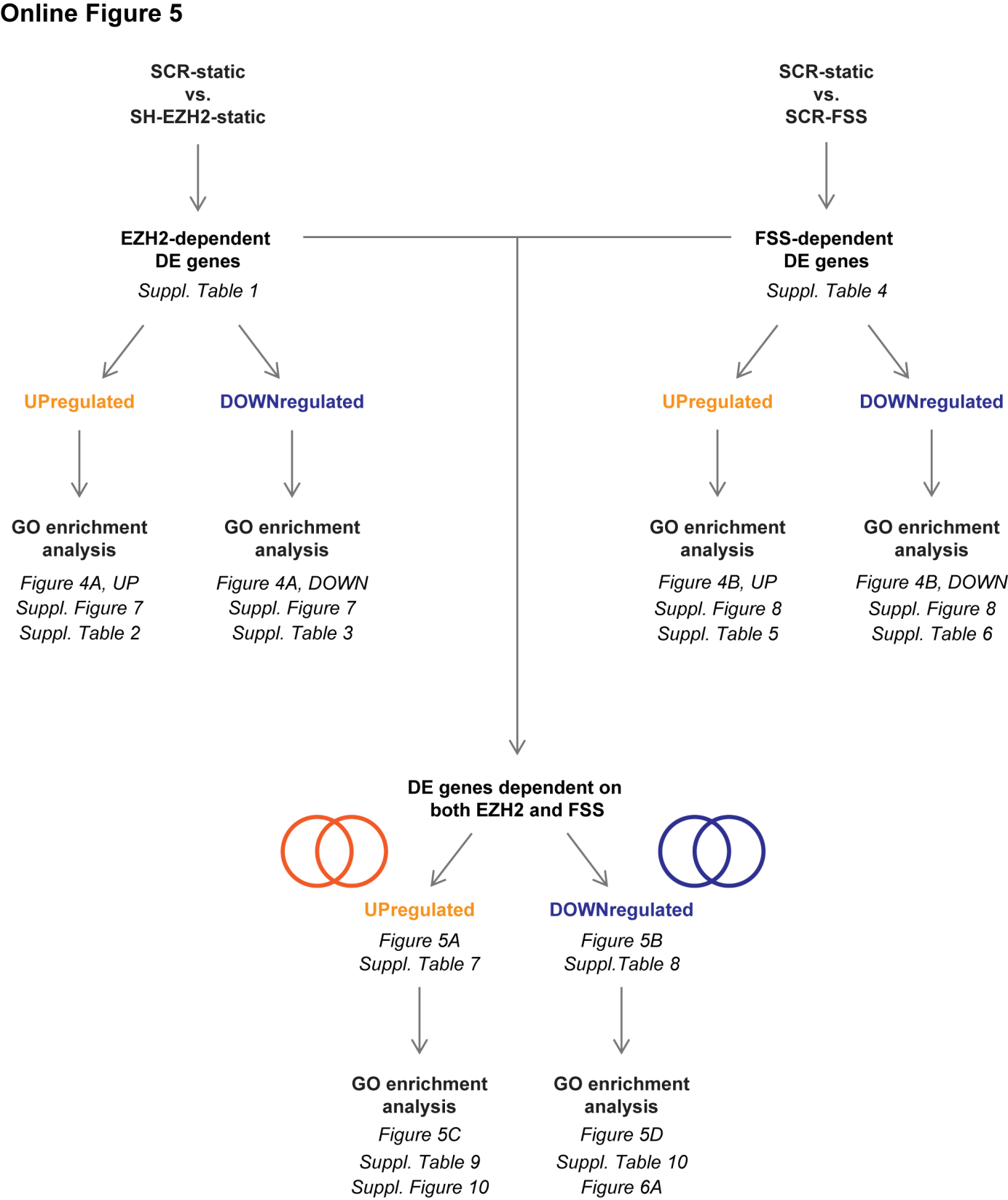


**Online Figure 5. Schematic representation of data analysis. DE – differential expression, GO – Gene Ontology.**


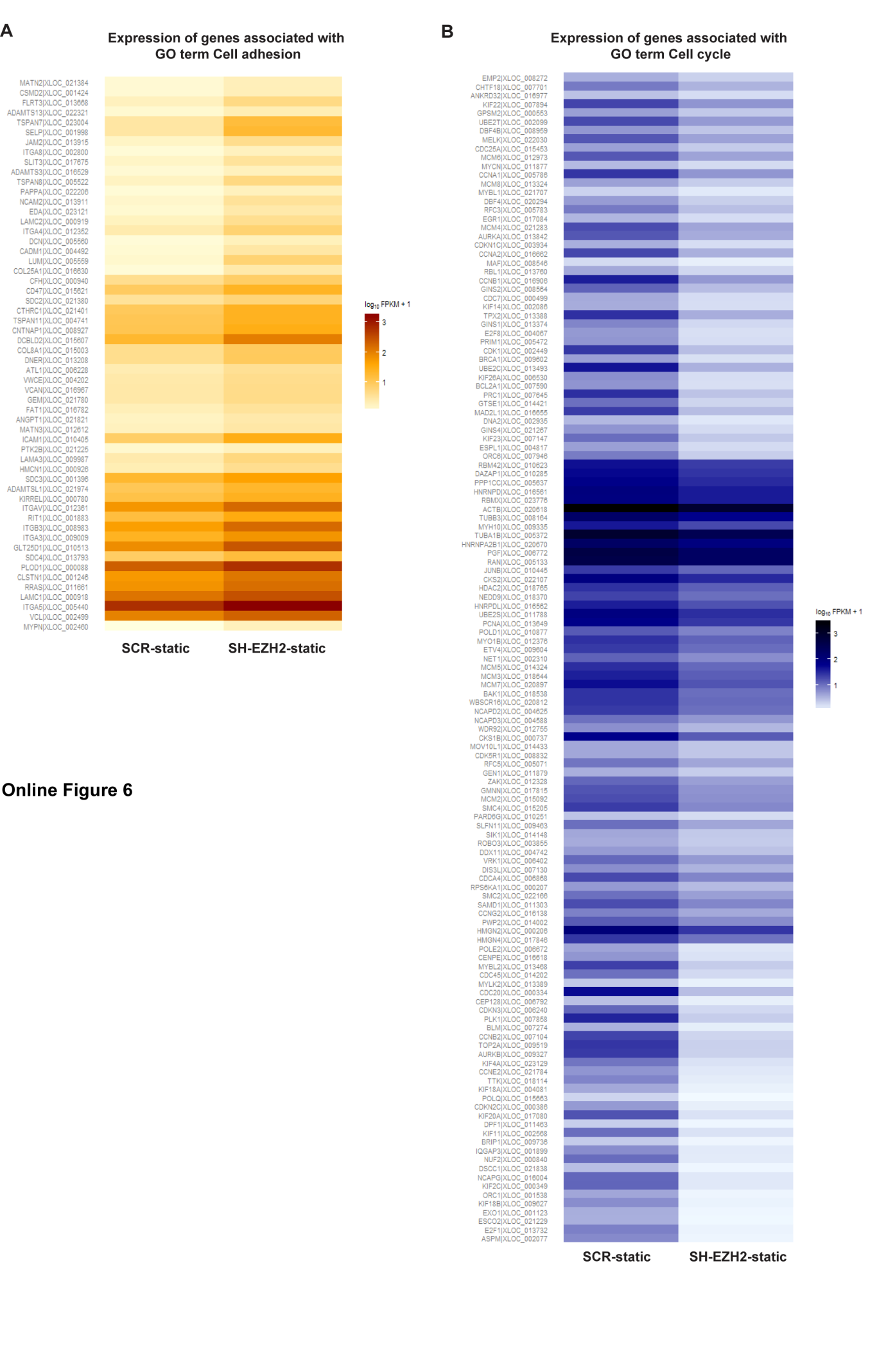


**Online Figure 6. Relative expression of genes regulated by EZH2, belonging to the most significantly enriched GO terms. A –** Heatmap representation of the relative expression of the genes upregulated by the EZH2-depletion that belong to the BP GO term Cell adhesion. **B –** Heatmap representation of the relative expression of the genes downregulated by the EZH2-depletion that belong to the BP GO term Cell cycle.


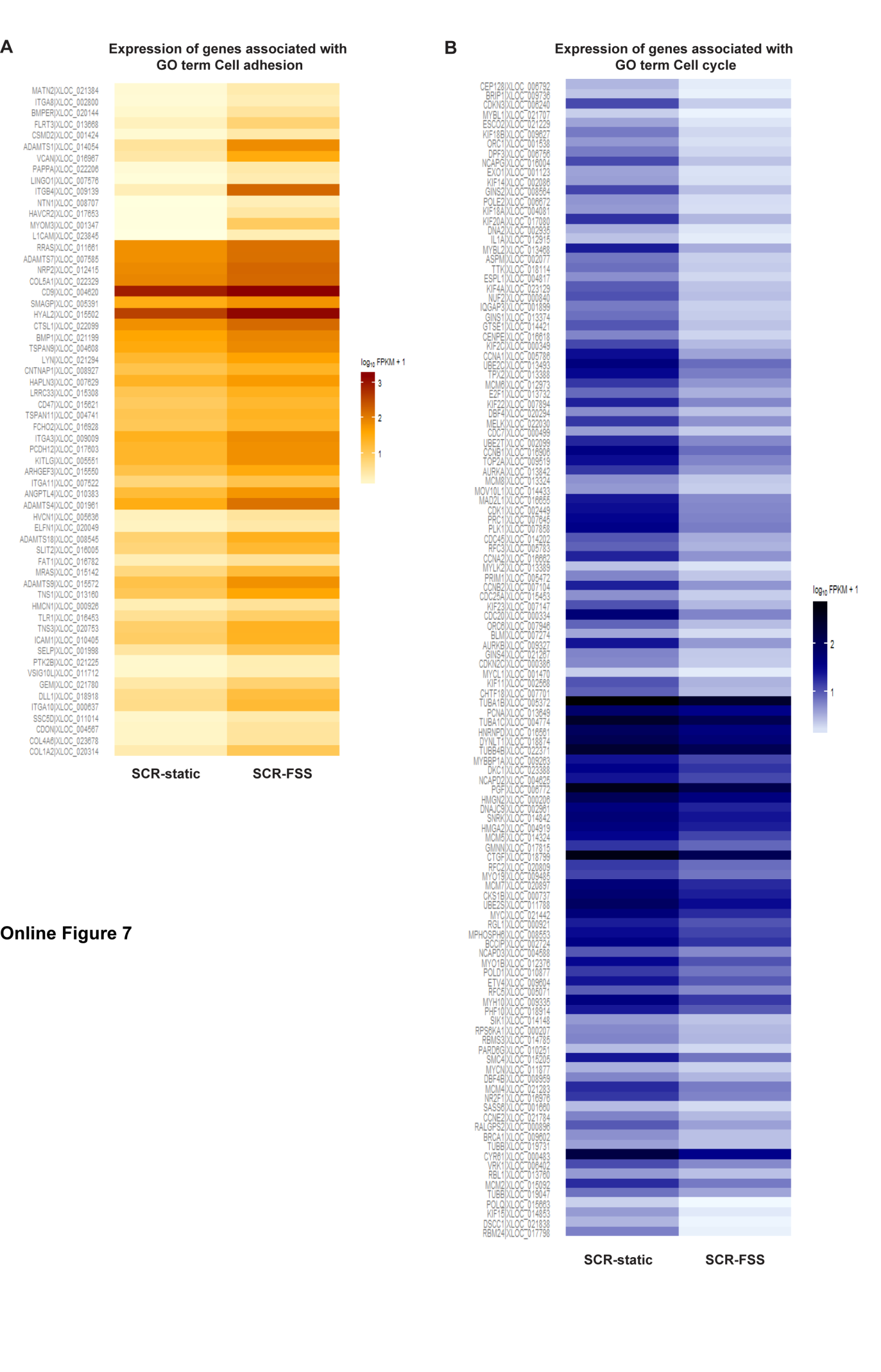


**Online Figure 7. Relative expression of genes regulated by FSS, belonging to the most significantly enriched GO terms. A –** Heatmap representation of the relative expression of the genes upregulated by the exposure to FSS, that belong to the BP GO term Cell adhesion. **B –** Heatmap representation of the relative expression of the genes downregulated by the exposure to FSS, that belong to the BP GO term Cell cycle.

**
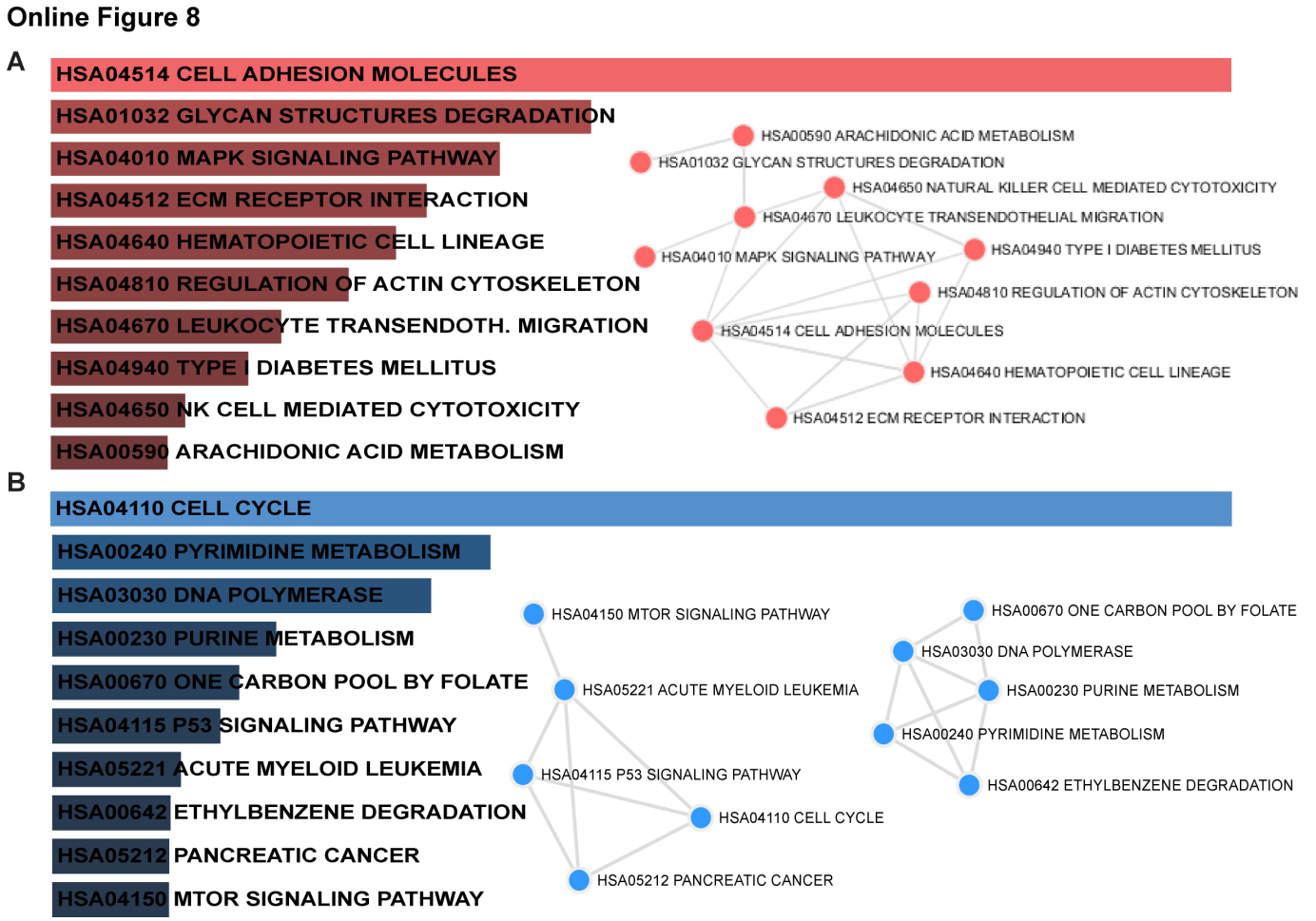
**

**Online Figure 8. Pathway enrichment analysis of the genes upregulated or downregulated by both EZH2-depletion and FSS-exposure.** **A –** Pathway enrichment analysis of the 103 genes upregulated by both EZH2-depletion and FSS-exposure. **B –** Pathway enrichment analysis of the list of the 355 genes downregulated by both EZH2-depletion and FSS-exposure. The lists of genes were analysed using Enrichr and KEGG database, using the combined score ranking. The brighter the colour, the more significantly enriched the term is. The inserts represent the network of dependencies between the enriched terms (the force field has been neglected).

**
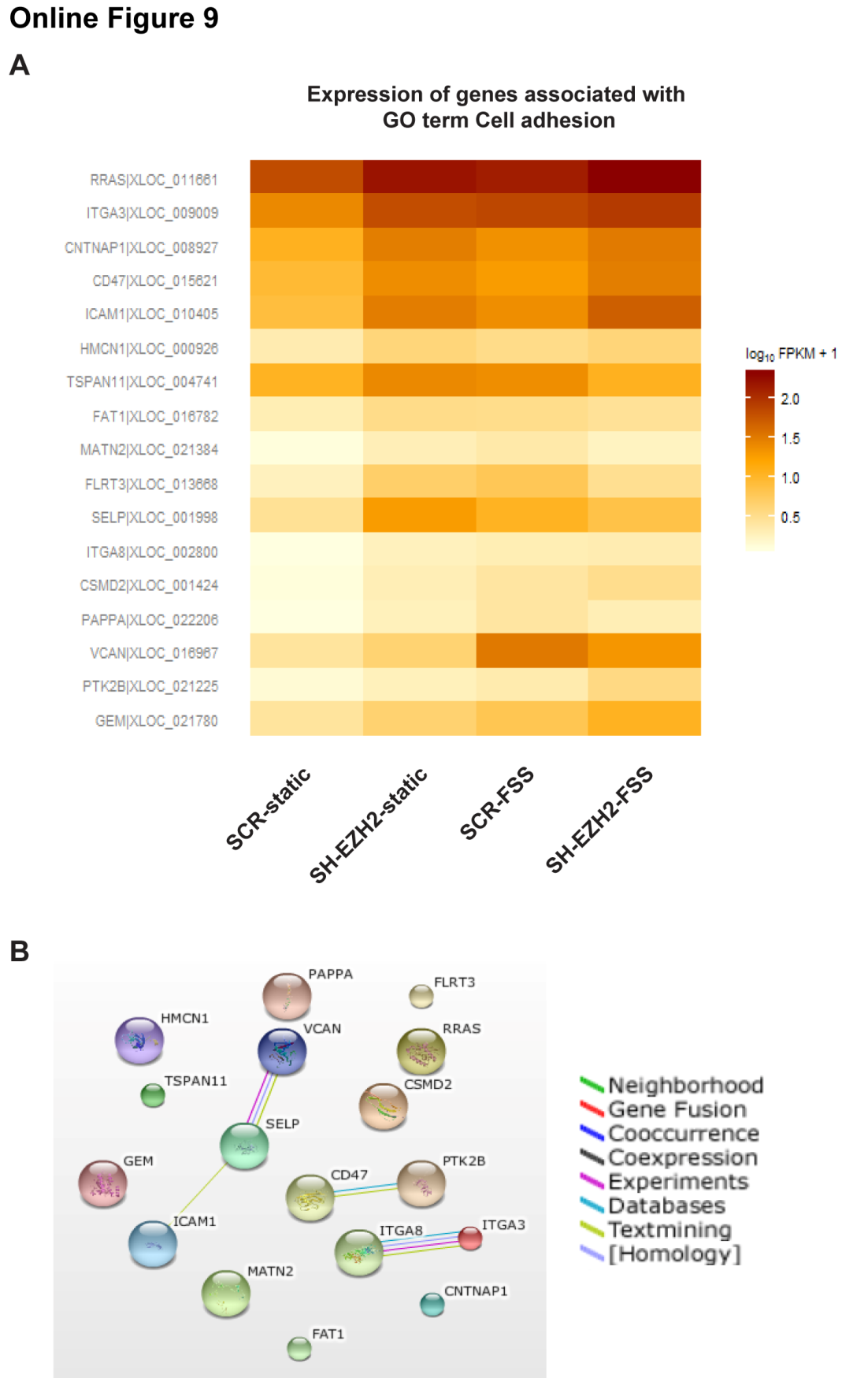
**

**Online Figure 9. Exploration of the cell adhesion-related genes upregulated by both EZH2 and FSS, identified based on the GO enrichment analysis.** **A –** A heatmap representation of the relative expression of the cell adhesion-related genes upregulated by both EZH2-depletion and FSS-exposure. **B –** Network representation of the mutual relationships of the products of the genes associated with the GO term cell adhesion, String 9.1, evidence view.

**Supplementary Tables Legends**

**Supplementary Table 1.** Genes up- or down-regulated 2 times or more by the depletion of EZH2 in HUVEC.

**Supplementary Table 2.** Biological Process Gene Ontology terms that were significantly overrepresented in the analysis of the list of genes upregulated 2 times or more by EZH-depletion.

**Supplementary Table 3.** Biological Process Gene Ontology terms that were significantly overrepresented in the analysis of the list of genes downregulated 2 times or more by EZH-depletion.

**Supplementary Table 4.** Genes up- or down-regulated 2 times or more by the exposure of HUVEC to FSS.

**Supplementary Table 5.** Biological Process Gene Ontology terms that were significantly overrepresented in the analysis of the list of genes upregulated 2 times or more by the exposure to FSS.

**Supplementary Table 6.** Biological Process Gene Ontology terms that were significantly overrepresented in the analysis of the list of genes downregulated 2 times or more by the exposure to FSS.

**Supplementary Table 7.** The 103 genes that were upregulated 2 times or more by both EZH2-depletion and the exposure to FSS.

**Supplementary Table 8.** The 355 genes that were downregulated 2 times or more by both EZH2-depletion and the exposure to FSS.

**Supplementary Table 9.** Biological Process Gene Ontology terms that were significantly overrepresented in the analysis of the list of the 103 genes upregulated 2 times or more by both EZH2-depletion and the exposure to FSS.

**Supplementary Table 10.** Biological Process Gene Ontology terms that were significantly overrepresented in the analysis of the list of the 355 genes upregulated 2 times or more by both EZH2-depletion and the exposure to FSS.
